# Unraveling the Mechanism of Hydrogen Atom Transfer by a Nickel-Hypochlorite Species and the Influence of Electronic Effects

**DOI:** 10.1021/acs.inorgchem.4c00360

**Published:** 2024-07-23

**Authors:** Adrià Juvanteny, Charafa Souilah, Raquel Quintero, Carlos García-Bellido, Neus Pagès-Vilà, Teresa Corona, Pedro Salvador, Anna Company

**Affiliations:** † Institut de Química Computacional i Catàlisi (IQCC), Departament de Química, Universitat de Girona, C/Maria Aurèlia Capmany 69, Girona 17003, Spain; ‡ Fachbereich Chemie, Philipps-Universitat Marburg, Hans-Meerwein-Str. 4, Marburg DE 35032, Germany; § Departamento de Química Inorgánica, CSIC and Universidad de Sevilla, Instituto de Investigaciones Químicas (IIQ), Sevilla 41092, Spain

## Abstract

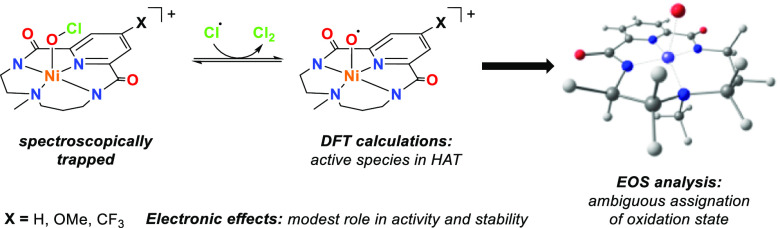

The oxidation of hydrocarbons is an important chemical transformation with relevance to biology and industry. Ni-catalyzed transformations are more scarce compared to Mn or Fe but have gained attention in recent years, affording efficient oxidations. Understanding the mechanism of action of these catalysts, including the detection and characterization of the active nickel–oxygen species, is of interest to design better catalysts. In this work, we undertake a theoretical study to unravel the mechanism of formation of the previously reported [Ni(OCl)(^H^L)]^+^ (^**H**^**2**) and how it activates C–H bonds. We disclose that the active species is indeed compound [Ni(O)(^H^L)]^+^, formed after homolytic cleavage of the O–Cl bond in ^**H**^**2** assisted by a chlorine radical. [Ni(O)(^H^L)]^+^ mediates C–H activation through an asynchronous concerted mechanism, in which the transition state is given by hydrogen atom transfer. Moreover, the electronic tuning of the ligand has a very modest impact on the stability and reactivity of the corresponding ^**X**^**2** species. Effective oxidation state analysis reveals an intriguing electronic structure of ^**H**^**2** and [Ni(O)(^H^L)]^+^, in which both the macrocycic ^H^L ligand and the OCl and O ligands behave as redox noninnocent. Such redox activity leads to a fully ambiguous oxidation state assignation.

## Abstract 2

Theoretical calculations show that the active species in C−H hydrogen atom abstraction corresponds to [Ni(O)(^H^L)]^+^, formed after homolytic cleavage of the O−Cl bond of the experimentally trapped [Ni(OCl)(^H^L)]^+^. Electronic tuning of the macrocyclic ligand has a very modest impact on the reactivity and stability of the corresponding active species, which show a controversial oxidation state assignment due to the redox noninnocence of the ligands, as ascertained by the effective oxidation state analysis.

## Introduction

Oxidation of aliphatic C–H and C=C bonds is important chemical transformations that have significant implications in biology where metalloenzymes are able to carry out such processes in a very efficient manner.^1^ In synthetic chemistry, several transition metals have been used to perform these processes with significant success. Although reports on nickel-catalyzed oxidation reactions are relatively scarce compared to other metals such as iron or manganese, in recent years, nickel-mediated C–H and C=C oxidation reactions affording high turnover numbers have been reported. Most commonly, these nickel catalysts contain nitrogen-based ligands that, in combination with *meta*-chloroperbenzoic acid (*m*CPBA) as an oxidant, afford high turnover numbers.^2−9^ The use of sodium hypochlorite as an oxidant in nickel-catalyzed oxidations has also been described albeit affording less efficient systems.^10,11^

Despite these published reports, details about the mechanism operating behind these transformations are unclear. Quite often, the involvement of (high-valent) nickel-oxygen species has been postulated,^12^ but examples of well-identified species with oxidizing abilities have been scarcely described. In this line, finding appropriate systems for the entrapment of these nickel-oxygen species and studying their reactivity are especially appealing to design better catalysts. By using carefully designed ligands bearing donating nitrogen donors, the characterization of several nickel-oxygen species has been achieved, and their oxidizing power has been evaluated. These compounds include nickel-acylperoxo species,^13^ nickel-oxyl,^14,15^ or nickel-hypochlorite species.^10,11^ Moreover, the oxidizing abilities of a series of well-characterized terminal nickel(III)-oxygen adducts or a nickel(IV)-nitrate species in hydrogen atom transfer reactions have been disclosed.^16−19^ Interestingly, the role of nickel as a simple radical initiator has also been proposed in alkane oxidation reactions using *m*CPBA as the oxidant.^20^

**Scheme 1:**
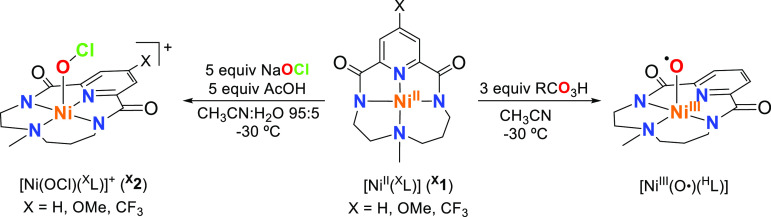
Schematic Representation of the Nickel(II) Precursors [Ni^II^(^X^L)] (^**X**^**1**) and the Nickel-Hypochlorite Species [Ni(OCl)(^X^L)]^+^ (^**X**^**2**) Studied in This Work, along with a Scheme of the Nickel-Oxyl Species [Ni(O^•^)(^H^L)] Previously Reported by Our Group^15^

In our group, we have used a tetradentate dianionic macrocyclic ligand with two amidates, one pyridine, and one aliphatic amine to stabilize two high-valent nickel-oxygen species. The first one was assigned as a nickel-oxyl species, [Ni(O^•^)(^H^L)] (Scheme 1), which is formed upon reaction of the nickel(II) precursor [Ni^II^(^H^L)] (^**H**^**1**, Scheme 1) with *m*CPBA.^15^ The second one corresponds to a nickel-OCl species with the general formula [Ni(OCl)(^H^L)]^+^ (^**H**^**2**, Scheme 1)^11^ that turned out to be roughly 5 times more reactive than [Ni(O^•^)(^H^L)]. Interestingly, kinetic studies suggest that ^**H**^**2** can even oxidize the strong C–H bonds of cyclohexane, and it is among the most reactive, well-defined nickel-oxygen species that have been reported to date. The electronic structure of ^**H**^**2** turned out to be particularly interesting. Even though the formal oxidation state of the nickel center in ^**H**^**2** is +4, our preliminary studies indicate that the compound is best defined as a nickel(III) center with spin density distributed over the bis-amidate and the hypochlorite ligands, which behave as redox noninnocent ligands.

The use of noninnocent ligands is a relatively common strategy to promote the oxidation of metal complexes while keeping the oxidation state of the metal center relatively low, as redox-active ligands serve as the pool of electrons. In nature, noninnocent ligands are also commonplace, and they have been identified in copper- and iron-based oxidase and oxygenase enzymes such as cytochrome P450, galactose oxidase, or intradiol cleaving dioxygenases.^21,22^ In the same line, the use of noninnocent ligands has ample precedents in the design of metal catalysts for reduction, oxidation, couplings, or polymerization reactions.^23^

In this work, we study by computational methods the mechanism of formation of ^**H**^**2** (by reaction of ^**H**^**1** with NaOCl) and its reaction with C–H bonds, unraveling the nature of the key oxidizing species in the process, which corresponds to a nickel-oxyl compound. Special attention is paid to the characterization of its intricate electronic structure. Given the noninnocent character of the macrocyclic bis-amidate ^H^L ligand, we also aim at experimentally tuning its electronic properties by adding an electron-donating methoxy group (^OMe^L) and an electron-withdrawing trifluoromethyl group (^CF3^L) in the γ position of the pyridine ring, which should affect the electronics of the bound nickel center without perturbing its steric properties, as previously reported for other systems.^24−26^ Thus, we also study the influence of electronics on the reactivity and stability of the resulting [Ni(OCl)(^X^L)]^+^ (^**X**^**2**) species.

## Results and Discussion

### DFT Calculations on the Formation of ^**H**^**2** from ^**H**^**1**

The oxidation of ^**H**^**1** with sodium hypochlorite in the presence of acetic acid has been modeled as an external sphere process by DFT methods, as described in Section 5.1 in the Supporting Information. Experimental results indicate that the addition of 3 equiv of ClO^–^ under acidic conditions leads to the formation of the Ni^III^ species [Ni(Cl)(^H^L)], as ascertained by UV–vis, EPR, and spectroelectrochemistry.^11^ This compound can be readily formed by oxidation of ^**H**^**1** with 1 equiv HOCl with the following reaction:
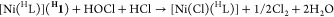
1where species HCl and HOCl are considered the predominant forms under acidic conditions. The reaction is highly exergonic with a Δ*G*_r_° of −27.6 kcal·mol^–1^.

According to our experimental evidence, further addition of up to 5 equiv of ClO^–^ results in the formation of ^**H**^**2**. In this case, DFT calculations indicate that ^**H**^**2** can be readily formed from [Ni(Cl)(^H^L)] by reaction with 2 equiv of HOCl, where the hypochlorite acts both as an oxidant and a ligand.
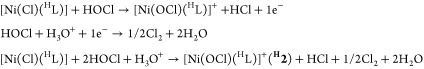
2

The reaction is again exergonic (Δ*G*_r_° = −19.9 kcal·mol^–1^) and accounts for the formation of ^**H**^**2** from [Ni(Cl)(^H^L)] under acidic conditions and in the presence of hypochlorite. The overall balance of the formation of ^**H**^**2** from ^**H**^**1** is the following:
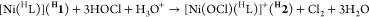
3

The process is highly exergonic (Δ*G*_r_° = −47.5 kcal·mol^–1^) and requires at least 3 equiv of HOCl to proceed. The previously reported calculations regarding the generation of ^**H**^**2** suggested its formation using only 2 equiv of HOCl,^11^ affording Δ*G*_r_° = −12.8 kcal·mol^–1^. Our calculations here indicate much more favorable thermodynamics considering 3 equiv of HOCl. This agrees fairly well with 5 equiv ClO^–^ that are experimentally required to maximize the formation of ^**H**^**2**, since one expects HOCl to undergo other side reactions.^27^

### DFT Calculations on the Mechanism of C–H Oxidation by ^**H**^**2**

We have previously described that ^**H**^**2** is kinetically competent to react with alkane substrates bearing C–H bonds with bond dissociation energies (BDE) ranging from ca. 75.5 to 99 kcal·mol^–1^. Experimental evidence suggested that the rate-determining step for this reaction corresponds to a hydrogen-atom transfer (HAT).^11^ To get more information about the reaction mechanism, the direct activation of C–H bonds by ^**H**^**2** was studied by using cyclohexane as a model substrate. Remarkably, all our attempts to find a reaction path that involved hydrogen abstraction were unsuccessful. The inclusion of other reactants such as a chlorine radical (Cl^•^) to assist O–Cl cleavage concomitant with C–H cleavage did not lead to any viable pathway. From this exploratory study, we concluded that ^**H**^**2** cannot directly perform the C–H oxidation reaction.

**Figure 1:**
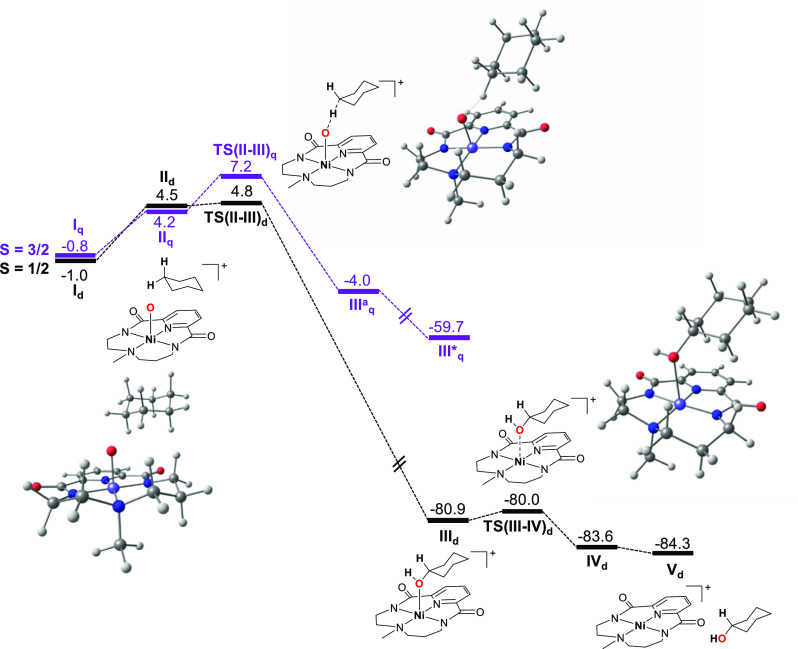
Reaction profile for the oxidation of cyclohexane carried out by the [Ni(O)(^H^L)]^+^ species computed at the B3LYP-D3(BJ)/def2-tzvp// B3LYP-D3(BJ)/def2-svp level of theory. Free energies are given in kcal·mol^–1^. The drawn structures correspond to intermediates and transition states of the *S* = 1/2 energy surface. Subscripts d and q represent spin states *S* = 1/2 and *S* = 3/2, respectively.

As an alternative to ^**H**^**2**, we studied if [Ni(O)(^H^L)]^+^ could be the active species responsible for the C–H cleavage of cyclohexane. In fact, experimental evidence indicates that under mass spectrometry conditions, the O–Cl bond of ^**H**^**2** breaks, and the only fragment observed corresponds to [Ni(O)(^H^L)]^+^.^11^ According to our calculations, the oxidation of cyclohexane by [Ni(O)(^H^L)]^+^ is thermodynamically feasible as shown in the reaction profile illustrated in Figure 1. First of all, several orientations of the incoming cyclohexane molecule with respect to [Ni(O)(^H^L)]^+^ were explored because of the asymmetry of the macrocyclic ligand and the axial/equatorial character of the C–H bond involved in the HAT process. All obtained prereactant complexes and transition states were within 0.5 kcal·mol^–1^. In the most favorable reaction pathway (Figure 1), an equatorial hydrogen of cyclohexane is abstracted by [Ni(O)(^H^L)]^+^, so that the corresponding axial C–H bond points outward in the reactant complex (**II**). This arrangement places the cyclohexane unit somewhat parallel to the macrocyclic ligand.

At the transition state corresponding to hydrogen abstraction (**TS(II–III)**_**d**_), the O···H distance is 1.53 Å. In the first steps of the intrinsic reaction coordinate (IRC), we observed a quick H atom transfer to form the transient [Ni(OH)(^H^L)]^+^ species, while the C_cyclohexane_···O distance remained essentially constant at around 2.6 Å. Then, as the Ni–OH bond rotates away from the substrate, the C···O distance shortens, directly forming a C–O bond and leading to **III**_**d**_, almost 80 kcal·mol^–1^ below **TS(II–III)**_**d**_. The IRC (see Figure S34) was carefully scrutinized, reducing the stepsize and searching for minimum energy structures along the pathway, but no additional intermediate was located. Thus, the oxidation of the C–H bond is best described as an *asynchronous concerted mechanism* in which the transition state is given by the HAT, which is followed by C–O bond formation without the generation of any intermediate along the process. The final release of the oxidized species (cyclohexanol) proceeds easily via an additional transition state **TS(III–IV)**_**d**_ with a very small barrier (∼1 kcal·mol^–1^).

Concerning the particular type of C–H bond cleavage, Figure S35 gathers the evolution of the partial charge and condensed spin density of the nickel macrocyclic ligand, OH ligand, and substrate along the first steps of the IRC. The evolution of the projected dipole moment is depicted in Figure S36. Once the new O–H bond is formed the substrate remains cationic during the IRC, thus indicating a formal hydride transfer. The mechanism of this transfer is, however, difficult to discern because of the delocalized nature of the electronic state of the species. For further details refer to the Supporting Information.

The above-mentioned reaction profile was obtained for an overall *S* = 1/2 spin state. Since the doublet and quartet electronic states of [Ni(O)(^H^L)]^+^ are almost degenerate (vide infra), we explored also the potential energy surface (PES) for *S* = 3/2. The *S* = 3/2 energetic profile is also shown in Figure 1. The **TS(II–III)**_**q**_ corresponding to the C–H cleavage is about 2 kcal·mol^–1^ higher in energy compared to the *S* = 1/2 profile. We have performed single-point triple-ζ energy calculations at the optimized TS structures using other DFT functionals such as wB97X-D and MN15. The open-shell singlet state is still favored by 2.4 and 4.0 kcal·mol^–1^, and most importantly, the electron distribution for both spin states is very similar to that originally obtained with B3LYP.

In the *S* = 3/2 PES, an intermediate **III**^**a**^_**q**_ is located 11.2 kcal·mol^–1^ below the TS before the rebound of the OH to the substrate. However, no TS for the rebound step was found. All efforts led to the high-energy species III^*^_q_ indicated in Figure 1 which is highly distorted. A possible explanation is that this path leaves the [Ni(^H^L)]^+^ species in a *S* = 3/2 state, which is more than 25 kcal·mol^–1^ over its ground *S* = 1/2 state, and it is also strongly geometrically distorted. Contrary to the rather complex situation of the *S* = 1/2 path, in the *S* = 3/2 state the reaction follows a clear HAT mechanism with no formal involvement of the Ni center. Further information is given in Figures S37 and S38 and accompanying discussion.

The question now is how active species [Ni(O)(^H^L)]^+^ can be formed under the reaction conditions before the oxidation of the substrate. The cleavage of the O–Cl bond from ^**H**^**2** to form [Ni(O)(^H^L)]^+^ was studied in detail. Direct homolytic cleavage of this bond turned out to be thermodynamically inaccessible in agreement with previous reports (see eq 4).^11^ In contrast, reaction with chlorine radical Cl^•^ to assist the O–Cl breakage makes this process thermodynamically feasible as the O–Cl and Cl–Cl bonds have very similar bond strengths (see eq 5). The presence of a chlorine radical in the reaction medium is plausible. Indeed, it is well established that hypochlorous acid is present in equilibrium with Cl_2_O and water to form chlorine oxide and chlorine radicals.^27,28^
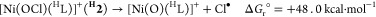
4
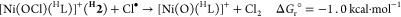
5

**Figure 2:**
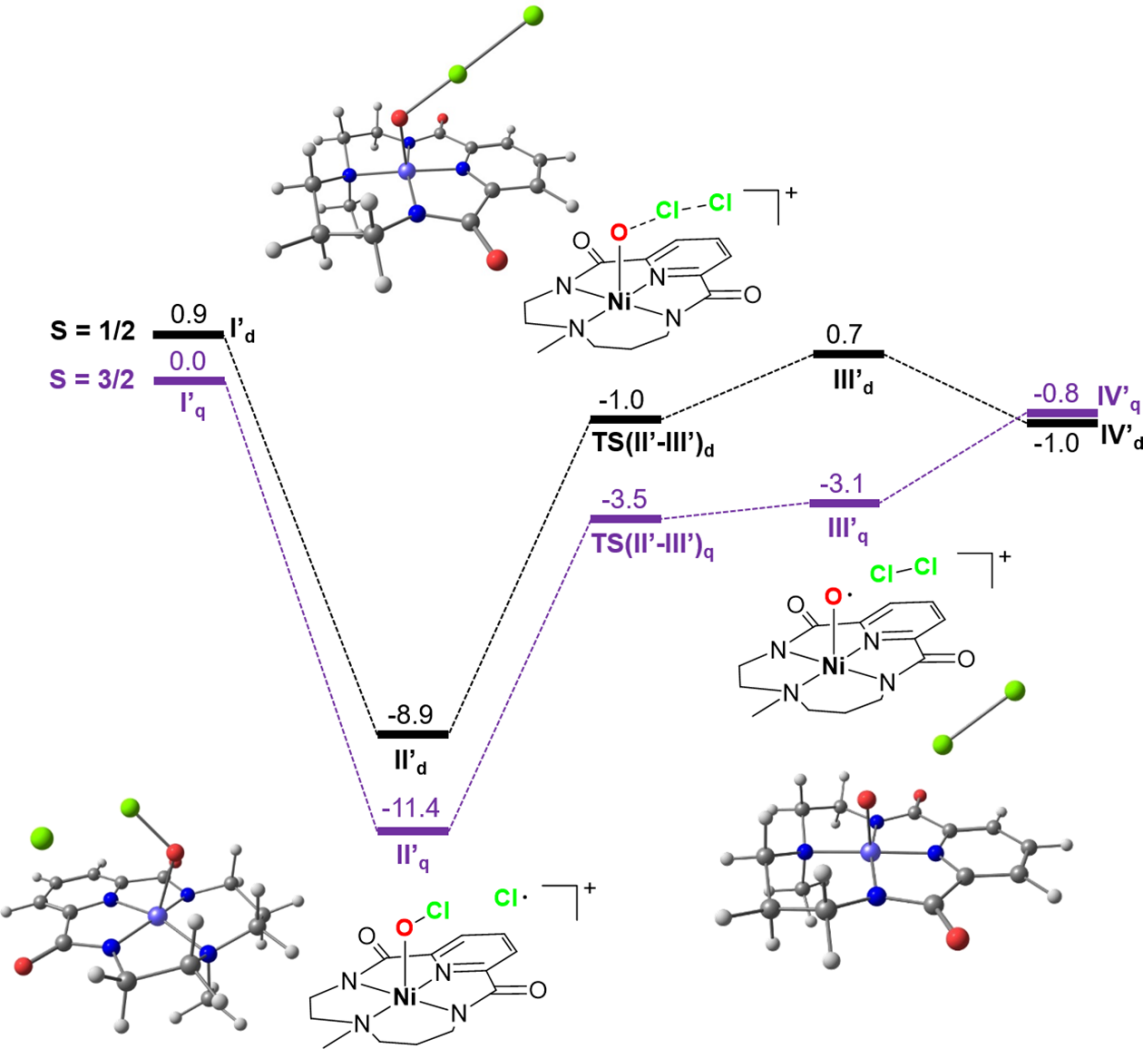
Reaction profile for the cleavage of the O−Cl group in [Ni(OCl)(^H^L)]^+^ assisted by a chlorine radical to afford [Ni(O)(^H^L)]^+^ computed at the B3LYP-D3(BJ)/def2-tzvp//B3LYP-D3(BJ)/def2-svp level of theory. Free energies are given in kcal·mol^–1^. Subscripts d and q represent spin states *S* = 1/2 and *S* = 3/2, respectively.

Figure 2 shows the reaction profile corresponding to the O–Cl cleavage in ^**H**^**2** assisted by Cl^•^. The most stable spin state for isolated ^**H**^**2** is the triplet (*S* = 1) albeit an open-shell singlet (broken symmetry) state is almost isoenergetic (within 1 kcal·mol^–1^). The closed-shell (*S* = 0) singlet state lies 9.1 kcal·mol^–1^ above the triplet ground state (vide infra). Together with the radical chlorine reactant overall *S* = 3/2 and *S* = 1/2 states are obtained and both PES were considered for this reaction. They lead to the formation of [Ni(O)(^H^L)]^+^ for which incidentally both spin states are also almost degenerate. In both PES as the Cl^•^ approaches ^**H**^**2** a stable reactant complex is found (**II’**_**q**_ and **II’**_**d**_) ca. 9–11 kcal·mol^–1^ below reactants. The basis set superposition error associated with the formation of the complex at the present level of theory was estimated to be 0.8 kcal·mol^–1^. The transition state for the O–Cl cleavage (**TS(II’–III’)**_**q**_ and **TS(II’–III’)**_**d**_) lies ca. 8 kcal·mol^–1^ above the reactant complex for both spin states and slightly below reactants and products (after Gibbs corrections). This indicates that the formation of [Ni(O)(^H^L)]^+^ from species ^**H**^**2** should be plausible.

We also considered the possibility that the homolytic cleavage of the Ni–OCl bond in [Ni(OCl)(^H^L)]^+^ could occur under our reaction conditions to form [Ni^III^(^H^L)]^+^ and ClO^•^ with the latter being the oxidizing species. In spite of being an exergonic process (Δ*G*_r_° = −4 kcal·mol^–1^), it is thermodynamically less favorable than the formation of the reactant complex proposed above (Figure 2, Δ*G*_r_° ∼ −10 kcal·mol^–1^), suggesting that our proposed NiO–Cl cleavage assisted by Cl^•^ is energetically more plausible. Moreover the experimental observation that the decay rate of [Ni(OCl)(^H^L)]^+^ is dependent on the type of substrate added and its concentration is not compatible with ClO^•^ being the oxidizing species. However, we do not discard that the homolytic cleavage of the Ni–OCl could be a background reaction in our system. Similarly, the direct involvement of the chlorine radical in the oxidation of the substrate cannot be ruled out, and its participation in background processes might also be possible.

**Scheme 2:**
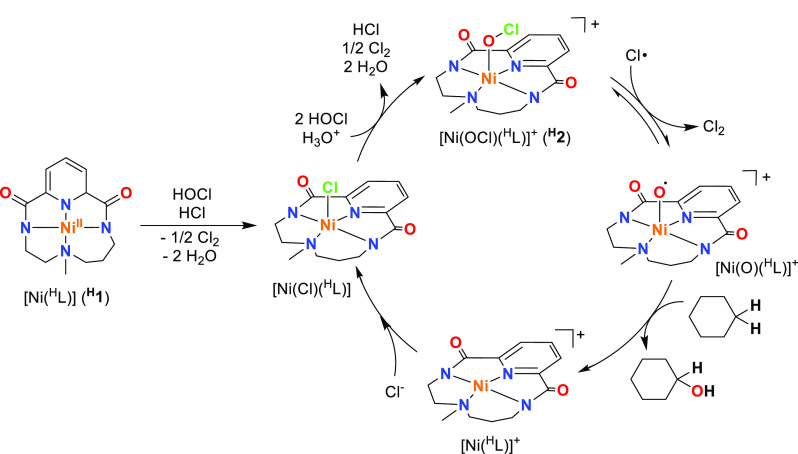
Overall Catalytic Process for the Oxidation of Cyclohexane Carried Out by ^**H**^**1** in Combination with HOCl in Acidic Conditions

The overall C–H oxidation pathway is summarized in Scheme 2. ^**H**^**1** species is oxidized by one equiv of HOCl to form the nickel(III)-chloride complex [Ni(Cl)(^H^L)]. Two more equivalents of HOCl are needed to form ^**H**^**2** species. The cleavage of the O–Cl bond would be assisted by radical chlorine atoms present in the media to form the active [Ni(O)(^H^L)]^+^ species for catalysis that performs activation of the C–H bond. After oxidation of the substrate, the resulting [Ni(^H^L)]^+^ species can readily capture a chlorine anion from the reaction medium to regenerate the aforementioned compound [Ni(Cl)(^H^L)] with an additional energy lowering of Δ*G*_r_° = −10.7 kcal·mol^–1^ to close the catalytic cycle.

The rate-determining *states* (as discussed by Kozuch and Shaik)^29^ of the catalytic cycle correspond to the reactant complex **II’** in Figure 2 (rate-determining intermediate) and the transition state involving the cleavage of the C–H bond (**TS(II–III)** in Figure 1, rate-determining transition state). The overall energetic spans of the cycle are 13.7 kcal·mol^–1^ and 18.6 kcal·mol^–1^ in the low- and high-spin surfaces, respectively. Due to the near degeneracy of the open-shell singlet and triplet states of [Ni(OCl)(^H^L)]^+^, the overall *S* = 3/2 pathway could still be plausible provided that a spin-crossing occurs after the rebound step.

### Synthesis and Characterization of [Ni^II^(^X^L)] (^**X**^**1**) Species

In order to evaluate how the electronic properties of the nickel center can influence the reactivity of the resulting nickel-oxygen species, we synthesized two modified versions of ^H^L containing a methoxy (^OMe^L) or a trifluoromethyl (^CF3^L) group in the *para* position of the pyridine ring. Synthesis of the protonated bis(amidate) macrocyclic ligands (H_2_^X^L) entailed the preparation of the corresponding 4-substituted pyridine-2,6-dicarbonyl dichlorides, which was carried out following slight modifications of previously reported procedures (see Supporting Information for more details). The key step in the preparation of the ligands was the last cyclization reaction between the acid chlorides and the triamine backbone. To favor the formation of the desired 1 + 1 macrocycles, reactions were carried out under high dilution conditions with slow addition of the triamine over the dicarbonyl dichloride. A solvent mixture consisting of toluene and dichloromethane was employed to ensure the solubilization of all of the reagents. Despite these considerations, the yield of H_2_^OMe^L and H_2_^CF3^L in the last cyclization step remained very low with yields of 21% and 8%, respectively.

**Figure 3:**
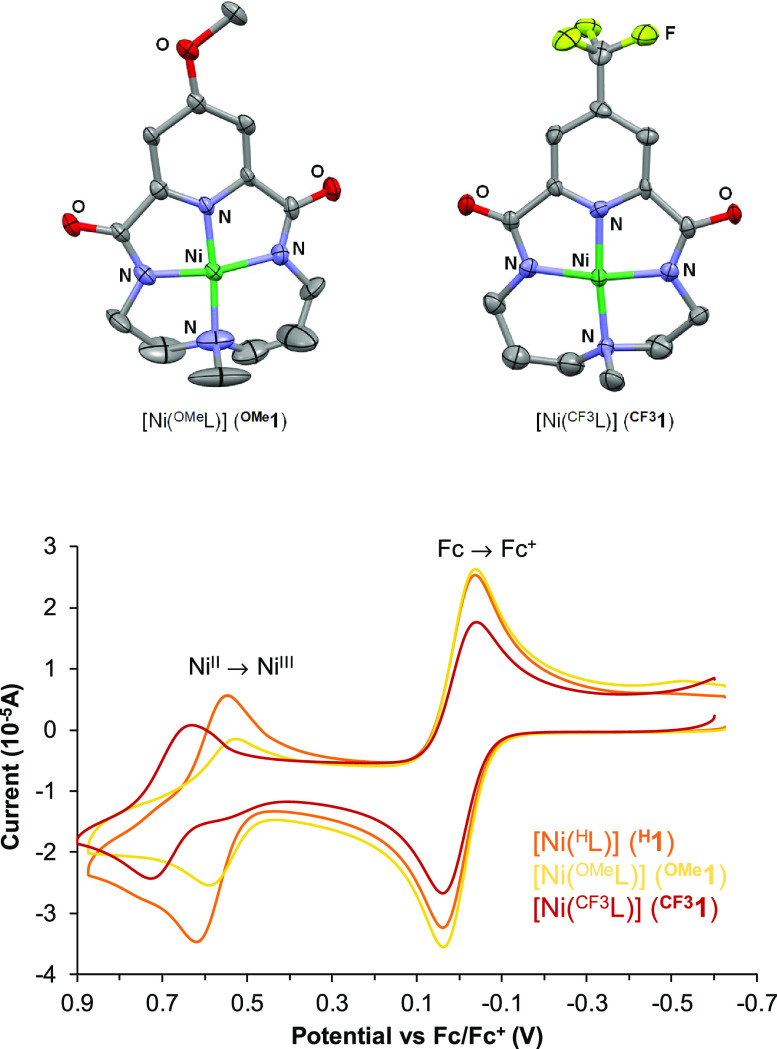
Top: X-ray structures of compounds [Ni(^OMe^L)] (^**OMe**^**1**) and [Ni(^CF3^L)] (^**CF3**^**1**). Hydrogen atoms and cocrystallized NaCF_3_SO_3_ have been omitted for clarity. Bottom: cyclic voltammograms of ^**H**^**1**, ^**OMe**^**1**, and ^**CF3**^**1** in CH_3_CN using ferrocene as an internal standard.

Synthesis of the corresponding nickel(II) complexes was carried out following a procedure similar to that previously reported for [Ni(^H^L)] (^**H**^**1**).^11^ Thus, equimolar amounts of [Ni(CF_3_SO_3_)_2_(CH_3_CN)_2_] and H_2_^X^L were mixed together with 2 equiv NaH under N_2_ in anhydrous acetonitrile for a few hours at room temperature. Solvent evaporation and crystallization of the resulting solid by slow diethyl ether diffusion into a methanol solution afforded the desired [Ni(^OMe^L)] (^**OMe**^**1**) and [Ni(^CF3^L)] (^**CF3**^**1**) complexes as yellow and deep red crystals, respectively. As ascertained by X-ray crystallography (Figure 3 top) and as previously observed for ^**H**^**1**, ^**OMe**^**1**, and ^**CF3**^**1** cocrystallized with NaCF_3_SO_3_. The nickel center presents a square planar geometry, and it is bound to the pyridine, the two amidate groups and the methylated amine of the ligand, exhibiting Ni–N distances typical of square planar Ni^II^ complexes, being the Ni–N_py_ distance the shortest (∼ 1.80 Å) followed by Ni–N_amide_ (∼ 1.85 Å) and Ni–N_CH3_ (∼ 1.88 Å). Due to the square planar geometry of the d^8^ nickel(II) center, ^**OMe**^**1** and ^**CF3**^**1** are diamagnetic species that could be further characterized by ^1^H NMR. Spectra are particularly complicated due to the lack of *C*_2_ symmetry of the system. This way, pyridine β protons appear as two separate signals, and all the aliphatic methylenic protons are nonequivalent so that each of them corresponds to a separate signal with complex multiplicities (Figures S3 and S4). High-resolution QTOF-MS analyses further confirmed the identity of the complexes in solution with the presence of characteristic peaks corresponding to [M + Na]^+^ at *m*/*z* 371.0636 and 409.0393 for ^**OMe**^**1** and ^**CF3**^**1**, respectively. The influence of the electronic properties of the pyridine ring on the nickel center was clearly evidenced by measurement of the redox potential of the Ni^III^/Ni^II^ redox pairs, which shows that this value is higher as the pyridine ring becomes more electron-poor. Thus, the measured *E*_1/2_ values (vs Fc/Fc^+^) were 0.56, 0.58, and 0.68 V for ^**OMe**^**1**, ^**H**^**1**, and ^**CF3**^**1**, respectively (Figure 3 bottom).

### Synthesis and Reactivity of [Ni(OCl)(^X^L)]^+^ (^**X**^**2**)

Following a methodology analogous to that previously reported for the ^**H**^**1**,^11^ reaction of compounds ^**OMe**^**1** and ^**CF3**^**1** with sodium hypochlorite in the presence of acetic acid was monitored by UV–vis spectroscopy at −30 °C using a CH_3_CN:H_2_O 95:5 solvent mixture. In both cases, the addition of 5 equiv sodium hypochlorite to a solution containing ^**OMe**^**1** or ^**CF3**^**1** in the presence of 5 equiv acetic acid afforded the formation of an intense band centered at 467 nm (ε = 9200 M^–1^cm^–1^) and 472 nm (ε = 8200 M^–1^cm^–1^), respectively (Figures S24 and S25). By analogy to the results previously reported for ^**H**^**1**, these chromophores correspond to the corresponding [Ni(OCl)(^X^L)]^+^ species (^**OMe**^**2** or ^**CF3**^**2**).

Similarly to the results observed for [Ni(OCl)(^H^L)]^+^ (^**H**^**2**), the chromophoric species ^**OMe**^**2** or ^**CF3**^**2** are not stable at −30 °C, and the intensity of the ∼475 nm band decreases over time. The measured half-life times (*t*_1/2_) are 4.5 h for ^**OMe**^**2** and 1.1 h for ^**CF3**^**2**. The higher stability of ^**OMe**^**2** could be rationalized by the electron-donating character of the methoxy group, which should stabilize the positive charge on the metal. The electron-withdrawing properties of the CF_3_ group should have the opposite effect, thus destabilizing ^**CF3**^**2**. Accordingly, the stability of ^**H**^**2** (*t*_1/2_ = 3.8 h) falls between the values measured for ^**OMe**^**2** or ^**CF3**^**2**.

In order to evaluate if the electronic properties play a significant role in the reactivity, the kinetics of the reaction of compound ^**X**^**2** was studied in oxygen atom transfer (OAT) and hydrogen atom transfer (HAT). 1-Octene was used as the substrate to evaluate their OAT abilities. Thus, under conditions of excess substrate, the absorption band of ^**OMe**^**2** and ^**CF3**^**2** (λ ∼ 470 nm) showed a pseudo-first-order decay and could be fitted to a monoexponential function from which observed rate constants (*k*_obs_) were extracted. The linear variation of *k*_obs_ with substrate concentration enabled the calculation of the corresponding second-order rate constants (Figure S33). The experimentally determined values were 0.31, 0.22, and 0.69 M^–1^s^–1^ for ^**OMe**^**2**, ^**H**^**2**, and ^**CF3**^**2**, respectively (Table 1). The trifluoromethyl substituent in ^**CF3**^**2** causes a slight acceleration of the reaction rate. Instead, the reaction rates for ^**OMe**^**2** and ^**H**^**2** are very close to one another with a slightly faster reactivity for the methoxy-substituted system. The HAT reactivity was measured using 1,4-cyclohexadiene as a substrate. In this case, the experimentally determined reaction rates were 6.7, 6.9, and 12.9 M^–1^s^–1^ for ^**OMe**^**2**, ^**H**^**2**, and ^**CF3**^**2**, respectively (Figure S29). Like the results obtained in the OAT, the electron-withdrawing group causes a 2-fold acceleration in the reaction rate, while minimal differences are found between the nonsubstituted and the methoxy-substituted systems. Overall, these kinetic results suggest that electronic effects can slightly tune the electrophilicity of the nickel center in ^**X**^**2** albeit their impact is rather modest (Table 1).

**Table 1:** Second-Order Rate Constants for the Reaction of [Ni(OCl)(^X^L)]^+^ (^**X**^**2**) with 1-Octene and 1,4-Cyclohexadiene in CH_3_CN:H_2_O 95:5 at −30 °C

compound	1-octene, *k*_2_, M^–1^s^–1^	1,4-cyclohexadiene, *k*_2_, M^–1^s^–1^
[Ni(OCl)(^OMe^L)]^+^ (^**OMe**^**2**)	0.31 ± 0.02	6.7 ± 0.3
[Ni(OCl)(^H^L)]^+^ (^**H**^**2**)	0.22 ± 0.02	6.9 ± 0.4
[Ni(OCl)(^CF3^L)]^+^ (^**CF3**^**2**)	0.69 ± 0.01	12.9 ± 0.8

### DFT Calculations on the Mechanism of C–H Oxidation by ^**x**^**2**

We repeated the DFT calculations for the mechanism of C–H oxidation using substituted catalysts ^**OMe**^**2** and ^**CF3**^**2.** The key aspects of the mechanism remain unaltered by the substitution. The energy profiles for the formation of the active species are shown in the Supporting Information (Figures S39 and S40). The triplet and open-shell singlet states of ^**OMe**^**2** and ^**CF3**^**2** are again almost degenerate. Similarly, the doublet and quartet states of the active [Ni(O)(^OMe^L)]^+^ and [Ni(O)(^CF3^L)]^+^ species are also almost degenerate, and the O–Cl cleavage is essentially isoenergetic. The barrier for the cleavage of the O–Cl from the reactant complex with Cl^•^ is slightly smaller for the ^**CF3**^**2** species. This can explain the experimental observation that ^**CF3**^**2** is somewhat less stable than ^**OMe**^**2** or ^**H**^**2**. The difference in the measured half-life times (see above) roughly corresponds to a difference of ca. 0.5 kcal·mol^–1^ in the activation barrier.

As far as the C–H activation process is concerned, the reaction proceeds via essentially the same low-spin mechanism described for ^**H**^**2** (Figures S41 and S42). The energetic span of the reaction given by the rate-determining transition state, equivalent to **TS(II–III)**_**d**_ in Figure 1, and the reactant complex [Ni(OC)(^X^L)]^+^···Cl^•^ (rate-determining intermediate, equivalent to **II’**_**d**_ in Figure 2) for the reaction of cyclohexane with [Ni(O)(^OMe^L)]^+^ or [Ni(O)(^CF3^L)]^+^ are 14.3 and 13.4 kcal·mol^–1^, respectively. The equivalent value for the [Ni(O)(^H^L)]^+^ profile is 13.7 kcal·mol^–1^. The experimentally determined 2-fold increase in the reaction rate for the C–H activation carried out by ^**CF3**^**2** with respect to ^**H**^**2** and ^**OMe**^**2** corresponds at the given working temperature to a 0.34 kcal·mol^–1^ difference in the activation barrier. Such subtle energy differences are well below the accuracy that one can expect for the DFT modeling at the current level of theory, and they have been previously observed in the reactivity of other metal–oxygen species, such as the nickel(III)-hydroxo species reported by Tolman and coworkers.^30^

As observed in the energy profile for [Ni(O)(^H^L)]^+^ after the HAT transition state for the reaction of cyclohexane with [Ni(O)(^OMe^L)]^+^ or [Ni(O)(^CF3^L)]^+^, the system directly evolves to an intermediate where the C–O bond is already formed following an asynchronous concerted mechanism. The energetics of the rest of the mechanism are barely affected by the ligand substitution.

### Electronic Structure Characterization of the Nickel Intermediates

We undertook a detailed analysis of the electronic structure of the most relevant species involved in the generation of ^**H**^**2** and in the catalytic cycle for the oxidation of C–H bonds. In particular, we applied the effective oxidation states (EOS) analysis^31^ to elucidate the most appropriate oxidation states of the metal and the ligands for these species. EOS relies on the so-called effective fragment orbitals (EFOs)^32^ and their occupations to assign electrons (electron pairs in the case of restricted closed-shell species) to the fragments (metals and ligands). The EFOs are obtained for all fragments (e.g., ligands and metals) and sorted by decreasing the occupation number. Individual electrons are then assigned to those with higher occupations from which OS or formal charges can be readily obtained. The last occupied and the first unoccupied EFOs constitute the frontier EFOs (in terms of occupation). The larger the difference in their occupations, the more unambiguous the OS assignation is. A reliability index *R*(%) can be introduced from these occupations to quantify the extent to which the actual electronic distribution of the molecule can be described by the formal ionic model. Based on past experience, it is typically possible to establish unambiguous oxidation state assignments with *R* = 100% for textbook examples of transition metal compounds. However, for systems with more complex electronic structures, it is anticipated that *R* values around 60–70% would be more common.^33^

**Figure 4:**
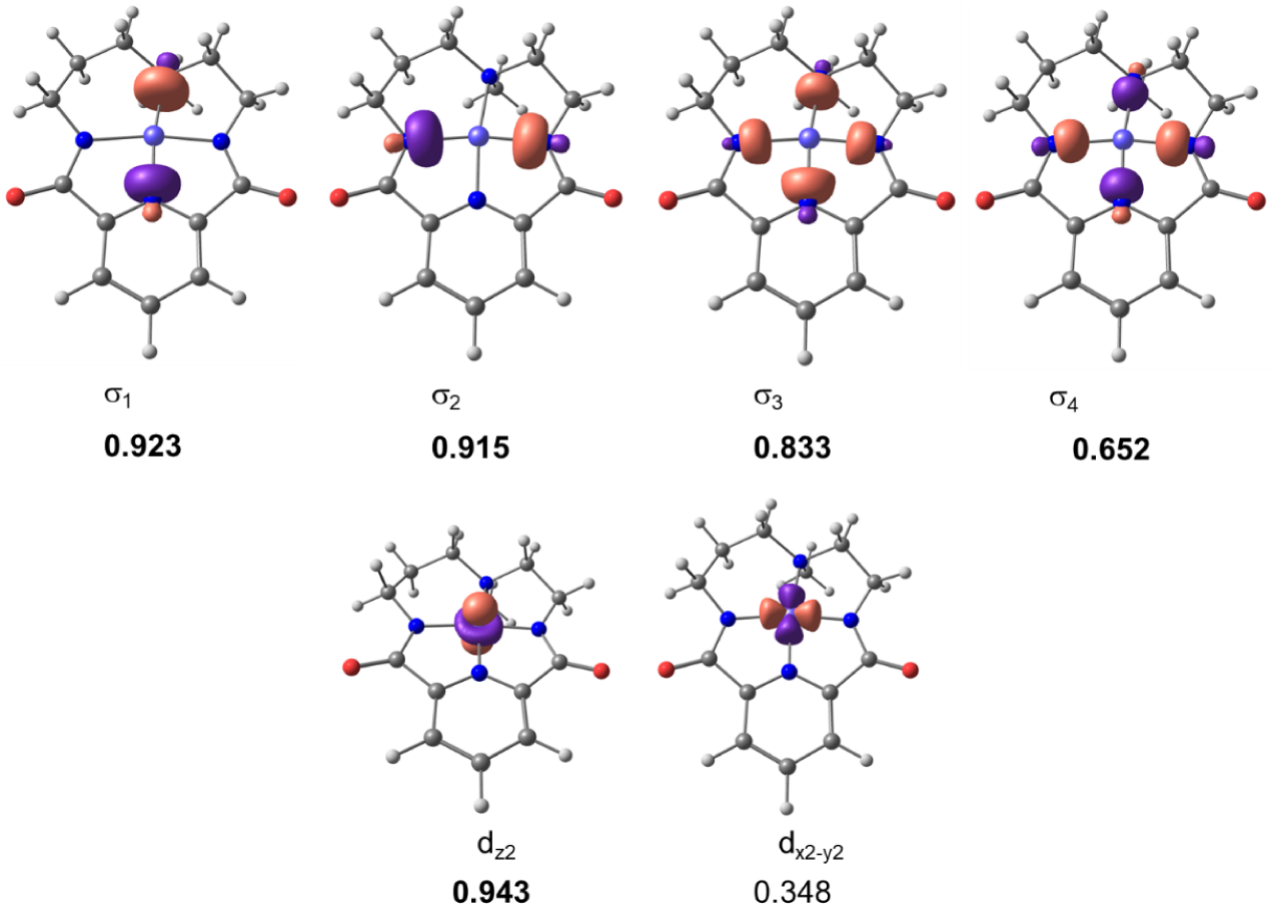
Relevant EFOs for the macrocyclic ligand (top) and Ni (bottom) and their occupation numbers for ^**H**^**1**. Occupations in bold indicate that the EFO is occupied in the EOS analysis. Frontier EFOs correspond to σ_4_ and the d_*x*2–*y*2_.

The starting nickel species ^**H**^**1** has a singlet ground state *S* = 0. The partial charge on Ni is +1.14. Because of the square planar coordination, the ligand–metal interaction bonding gives rise to the four valence EFOs of the ligand depicted at the top of Figure 4. In the case of the metal, the valence EFOs correspond to the 3d and 4s atomic orbitals polarized by the environment. Figure 4 shows the shape of the d_*x*2–y2_ and d_*z*2_ EFOs. Upon EOS analysis, all sigma-type EFOs of the ligand and four d-type EFOs of the metal are considered occupied, which leads to a Ni(+2) and a formal dianionic ligand (−2) assignation. The difference in occupation between the last occupied (0.652) and first unoccupied (0.348) EFOs, labeled σ_4_ and d_*x*2–_*_y_*_2_ in Figure 4, is large so the OS assignation is quite unambiguous (*R* = 80.4%). The same analysis of the substituted ^**OMe**^**1** and ^**CF3**^**1** species yielded virtually the same EFOs and occupations as those obtained for ^**H**^**1**. This suggests that no discernible electronic effects are affecting the ground state of these species upon ligand substitution.

The transient [Ni(Cl)(^H^L)] complex that is formed at the end of the catalytic cycle exhibits an *S* = 1/2 ground state. The shapes of the EFOs of the metal and macrocyclic ligand obtained for the alpha and beta part of the density are essentially the same as those depicted in Figure 4 except occupations differ. Upon oxidation, the beta occupation of the d_*z*2_ EFO of Ni decreases down to 0.325, thus becoming unoccupied, already indicating that the one-electron oxidation from ^**H**^**1** is metal-centered. Most of the spin density (0.74) is also located on Ni. Consequently EOS analysis indicates Ni(+3) and ^H^L(−2) situation. A concomitant enhancement of the donation from the σ_4_ EFO of the ligand to the d_*x*2–_*_y_*_2_ EFO of the metal compensates for the charge on Ni (+1.31), which is only slightly higher than that for ^**H**^**1**. As a result, the OS assignation has a much lower *R* value of 52.3%. The same mechanism operates in the case of the cationic [Ni(^H^L)]^+^ species, i.e., Ni(+3) assignation with a rather low *R* value of 55.4%. Again, no significant electronic effects are observed for the corresponding CF_3_ and OMe substituted species.

The electronic structure of ^**H**^**2** and the active species [Ni(O)(^H^L)]^+^ is much less clear because of important electron delocalization and spin polarization. As mentioned before, DFT calculations indicate that the triplet and open-shell singlet states of ^**H**^**2** are essentially degenerate upon thermal and entropic corrections. In the case of the *S* = 1 state, the partial charge on Ni is +1.35 very similar to that of the [Ni(Cl)(^H^L)] species but not far from that of ^**H**^**1**. The spin density, however, is completely delocalized in the complex. The spin populations are 0.75 for Ni, 0.44 for the OCl fragment, and 0.80 for the macrocyclic ligand.

In the case of the active species [Ni(O)(^H^L)]^+^, the *S* = 1/2 (doublet) and *S* = 3/2 (quartet) states are also virtually degenerate with an energy difference below 0.2 kcal·mol^–1^. The partial charges on Ni are +1.42 and +1.36, respectively. In the high-spin state, the spin density is again completely delocalized over Ni (0.81), oxygen (1.36), and the macrocyclic ligand (0.83). The *S* = 1/2 state shows a spin density mostly localized in the oxygen atom (0.98) with a non-negligible contribution from both Ni (−0.22) and the macrocyclic ligand (0.24).

**Figure 5:**
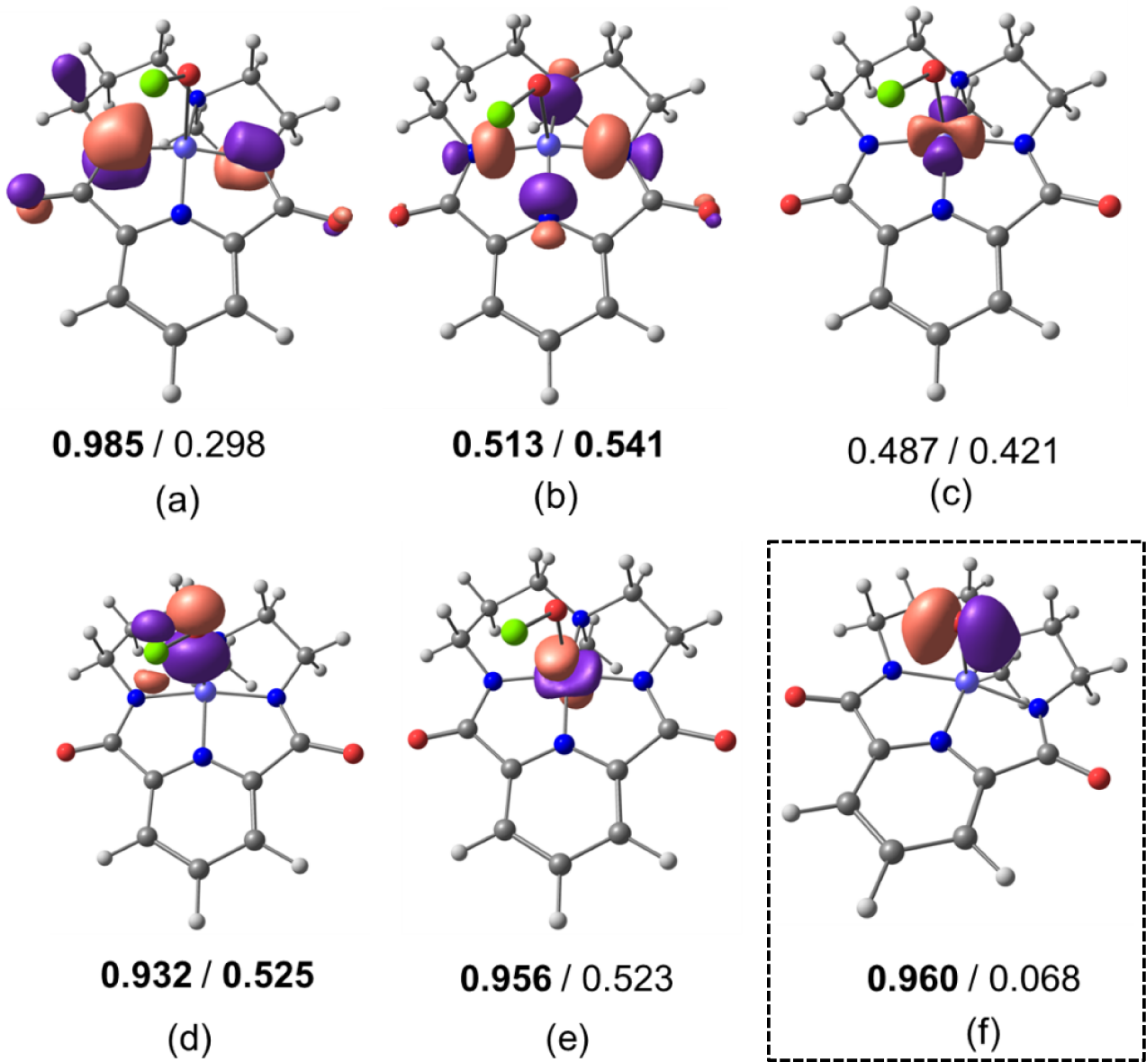
Frontier EFOs for the macrocyclic ligand (a, b), Ni (c, e), and the OCl ligand (d) along with their occupation numbers for ^**H**^**2** in the *S* = 1 state. In the dashed box, spin-polarized p-type EFO of oxygen in [Ni(O)(^H^L)]^+^ in the *S* = 3/2 state is given (f). Occupations in bold indicate that the EFO is occupied in the EOS analysis.

In all cases, EOS analysis results in completely ambiguous OS assignation, with several frontier EFOs exhibiting very similar occupations in both the alpha and beta parts of the density and therefore *R* values are close to 50%. The Ni center is best described as Ni(+3) and in any case very far from a formal Ni(+4) picture. In the high-spin states of ^**H**^**2** and [Ni(O)(^H^L)]^+^, the macrocyclic ligand exhibits π noninnocence. For instance for ^**H**^**2**, the EFO of the macrocyclic ligand depicted in Figure 5a shows a significant decrease in the beta occupation down to 0.298 and becomes formally unoccupied, providing L(−1) character. In addition, there is a large sigma donation from the σ_4_ EFO (Figure 5b) into the d_*x*2–_*_y_*_2_ EFO of Ni (Figure 5c), indicating sigma noninnocence. In the case of the low spin states, this donation is further enhanced and the respective σ_4_ and d_*x*2–_*_y_*_2_ occupations become almost equal, thus blurring the OS assignation. This analysis confirms the interpretation given in our previous study,^15^ where the ligand’s noninnocence was shown to be consistent with XAS data.

On the other hand, spin polarization is also manifested in the OCl and O ligands of ^**H**^**2** and [Ni(O)(^H^L)]^+^, respectively. In the high spin state of ^**H**^**2**, the beta occupation of the π*-type EFO on OCl associated with the σ bond with Ni is much lower than for the alpha counterpart (see Figure 5d). The same occurs for d_z2_ on Ni (Figure 5e). This is consistent with a 3e-2c Ni–O bond (the computed bond order is 0.57). The beta occupation of the π* OCl EFO (0.525) is barely larger than that of the d_*z*2_ EFO (0.523) on Ni, which becomes formally unoccupied giving Ni(3+) character. In the case of [Ni(O)(^H^L)]^+^, there is a decrease in the beta occupation of a p-type EFO on oxygen (see Figure 5f), which gives a marked oxyl (−1) character.

All in all, EOS analysis points to an extremely close-call situation for both compounds and in both spin-states, where the Ni center is still best described as Ni(+3), the macrocyclic ligand is formally oxidized to L(−1) and the OCl or O ligand is described as anionic (−1). The *R* ∼ 50% values, however, indicate that the OS assignation is fully ambiguous.

The substitution of the ligand to species ^**OMe**^**2** and ^**CF3**^**2** has no noticeable effect on the Ni partial charge or the spin density distribution. In the case of ^**CF3**^**2**, EOS analysis does indicate a significant increase in the occupation of the beta d_z2_ EFO of Ni going from 0.523 to 0.536 and a concomitant decrease of the π* OCl EFO going from 0.525 to 0.502. Thus, ^**CF3**^**2** exhibits somewhat less Ni(+3) character, but EOS analysis indicates that the electronic structure of these systems cannot be properly discussed from the point of view of the formal ionic model.

## Conclusions

In this work, we have disclosed by theoretical methods a possible mechanism of the formation of [Ni(OCl)(^H^L)]^+^ (^**H**^**2**) by reaction of the parent nickel(II) complex (^**H**^**1**) with sodium hypochlorite in the presence of acetic acid. Moreover, a mechanism for C–H bond oxidation carried out by ^**H**^**2** has been proposed. According to our calculations, this species is not directly responsible for the oxidation of the substrate but instead a homolytic O–Cl bond cleavage assisted by a chlorine radical occurs to form the active compound [Ni(O)(^H^L)]^+^. This compound is the one that abstracts the hydrogen atom from the targeted C–H bond, which eventually leads to its oxidation. The process is best described as an *asynchronous concerted mechanism* in which the transition state is given by the hydrogen atom transfer, which is followed by C–O bond formation without the generation of any intermediate along the process.

Moreover, we have synthesized two variants of ^**H**^**2** containing an electron-donating (OMe) and an electron-withdrawing (CF_3_) group in the pyridine ring of the macrocyclic ligand to evaluate how electronic effects influence the oxidizing abilities of the corresponding nickel-hypochlorite species ^**X**^**2**. Interestingly, the impact of the substituents is rather modest in both the hydrogen atom transfer and oxygen atom transfer reactions. This experimental result agrees well with theoretical calculations, which predict very small variations of the reaction barriers (less than 1 kcal·mol^–1^) upon the introduction of a CF_3_ or an OMe group in the macrocyclic ligand.

Finally, the electronic structure of the nickel species involved in the oxidation reactions has been evaluated using effective oxidation state (EOS) analysis. While the oxidation state assignment is rather clear for the starting ^**H**^**1** species or the nickel(III) compounds [Ni(Cl)(^H^L)] or [Ni(^H^L)]^+^, the situation becomes much more complex for the nickel-oxygen species ^**H**^**2** and [Ni(O)(^H^L)]^+^. In this case, the redox chemistry of the amidate groups of the ligand plays an important role so that ^H^L is oxidized by one electron. The oxidation state of the nickel is best described as +3, and the OCl and O ligands are best described as anionic ligands. However, the reliability index of this assignment is around 50%, which indicates that the oxidation state assignment is fully ambiguous due to both charge and spin delocalization among the metal and ligands.

## Supporting Information Available

Materials and methods, synthesis and characterization of ligands and complexes, general procedure for the generation of ^**X**^**2** and their reactivity and additional information on theoretical calculations (PDF)
The Cartesian coordinates of all species considered (XYZ)

## Supplementary Material

ic4c00360_si_001.pdf

ic4c00360_si_002.xyz
