# Supplementary material for: Unraveling the Mechanism of Hydrogen Atom Transfer by a Nickel-Hypochlorite Species and the Influence of Electronic Effects
Source: Inorg Chem. 2024 Jul 23;63(31):14325–34. doi: 10.1021/acs.inorgchem.4c00360 (PMC11304384; doi:10.1021/acs.inorgchem.4c00360)
Supplement: Supplementary file 1 — ic4c00360_si_001.pdf [file ic4c00360_si_001.pdf]

Supporting information for

Unraveling the mechanism of hydrogen atom transfer by a nickel-hypochlorite species and the influence of electronic effects

Adrià Juvanteny,[1] Charafa Souilah,[1,2] Raquel Quintero,[1] Carlos García-Bellido,[1,3] Neus Pagès-Vilà,[1] Teresa Corona,[1] Pedro Salvador,[1]* Anna Company[1]*

[1]Institut de Química Computacional i Catàlisi (IQCC), Departament de Química, Universitat de Girona, C/ Maria Aurèlia Capmany 69, 17003 Girona, Spain

[2]Philipps-Universitat Marburg, Fachbereich Chemie, Hans-Meerwein-Str. 4, DE 35032 Marburg, Germany

[3]Instituto de Investigaciones Químicas (IIQ), Departamento de Química Inorgánica, CSIC and Universidad de Sevilla, 41092 Sevilla, Spain

E-mail: pedro.salvador@udg.edu; anna.company@udg.edu

Outline

1. Materials and methods S3

2. Synthesis of ligands S4

2.1. Synthesis of $H_2{}^{OMe}L$ S4

2.2. Synthesis of $H_2{}^{CF3}L$ S7

3. Synthesis and characterization of nickel(II) complexes S11

3.1. Synthesis of [Ni(^{OMe}L)] and [Ni(^{CF3}L)] S11

3.2 ^{1}H-NMR and UV-vis spectra of [Ni(^{OMe}L)] and [Ni(^{CF3}L)] S12

3.3. Crystallographic data for [Ni(^{OMe}L)] and [Ni(^{CF3}L)] S23

4. Generation and reactivity of ^{X}2 .... S24

4.1. Generation of ^{X}2 .... S24

4.2. Kinetic analyses of the reaction of ^{X}2 with organic substrates. S25

5. Theoretical calculations S30

5.1. Computational details S30

5.2. Mechanism of the HAT S30

5.3. Additional figures for the mechanism of species ^{OMe}2 and ^{CF3}2 .... S34

5.4. Additional Figures for the Effective fragment orbitals (EFOs) S38

6. Bibliography S40

1. Materials and methods

All reagents and solvents used were commercially available and purchased from Merck, Panreac, Scharlau and Fluorochem. Preparation and handling of air-sensitive materials was carried out in a N_2 drybox (Jacomex) with O_2 and H_2O concentrations <1 ppm. (2-aminoethyl)(3-aminopropyl)methylamine was synthesized as previously reported.[1]

^{1}H-NMR, ^{13}C-NMR and ^{19}F-NMR spectra were recorded in a Bruker Ultrashield Avance III400 and Ultrashield DPX300 spectrometers. Mass spectra were performed by electrospray ionization source in a high-resolution mass spectrometer Bruker micrOTOF QII (Q-TOF) with a quadrupole analyzer with positive ionization mode or in an Esquire 600 mass spectrometer with an ionic trap with positive ionization mode coupled to an HPLC UV-Vis Agilent 1200. UV/Vis spectroscopy was performed in an Agilent 8453 UV/Vis spectrophotometer with 1 cm quartz cells. Low temperature control was achieved with a cryostat from Unisoku Scientific Instruments, Japan. The X-ray intensity data were measured on a Bruker D8 QUEST ECO three-circle diffractometer system equipped with a Ceramic X-ray tube (Mo Kα, λ = 0.71076 Å) and a doubly curved silicon crystal Bruker Triumph monochromato).

Cyclic voltammetries were performed with a CHI 620D Electrochemical Analyzer using a three electrode cell. The working electrode is a glassy carbon disk from BAS (0.07 cm^2), the reference electrode is a Ag/$AgNO_3$ (10 mM) and the auxiliary electrode is platinum wire. All voltammetries have been carried out with $n$$Bu_4NPF_6$ (TBAP) as supporting electrolyte (0.1 M ionic strength). All values are based on the Fc/Fc^+ redox couple as internal reference.

2. Synthesis of ligands

2.1. Synthesis of $H_2^{OMe}L$

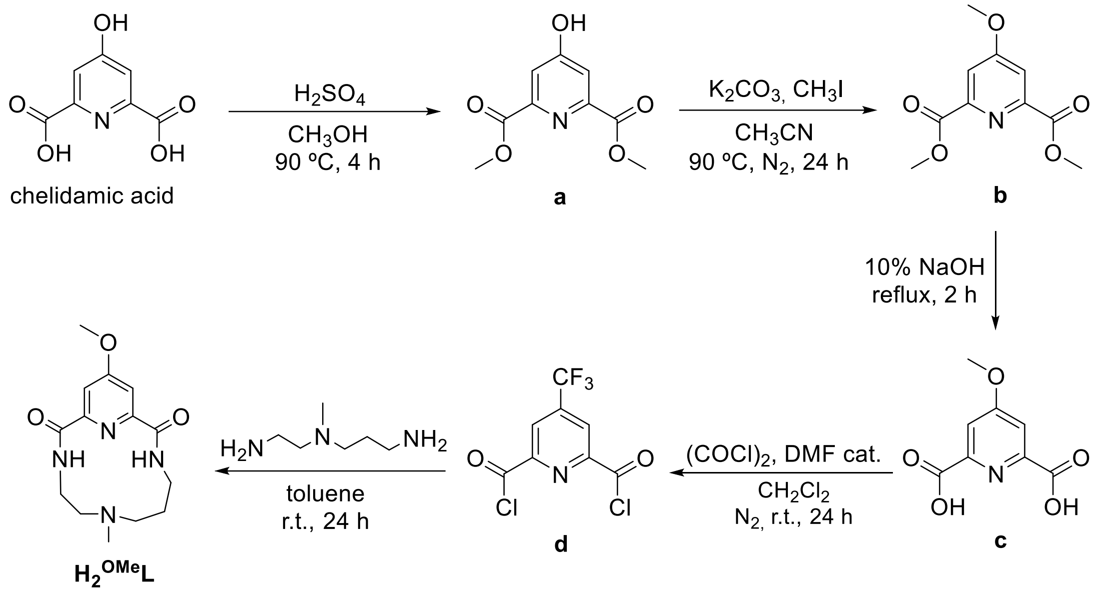

Scheme S1. Synthetic route for the synthesis of **$H_2^{OMe}L$**.

Synthesis of dimethyl 4-hydroxypyridine-2,6-dicarboxylate (a). This compound was prepared following a slight modification of a literature procedure.[2] Chelidamic acid hydrate (6.75 g, 33.5 mmol) was suspended in methanol (210 mL) and sulfuric acid (97%) (615 µL, 11.2 mmol) was added dropwise at room temperature with vigorous stirring. The yellow solution was refluxed at 90 °C for 4 hours. After this period the solvent was evaporated. Water (310 mL) was then added and the solution was extracted with ethyl acetate (4 x 200 mL). The organic phase was dried with anhydrous $MgSO_4$, filtered and the solvent was removed under reduced pressure to give compound **a** as a white solid (6.03 g, 28.6 mmol, 85% yield). ^{1}H-NMR (d_6-DMSO, 300 MHz, 298 K) δ, ppm: 7.56 (s, 2H, H_{Py}), 3.88 (s, 6H, OC**H3**). ^{13}C-NMR (d_6-DMSO, 75 MHz, 298 K) δ, ppm: 165.90 (**C**=O), 164.81 (**C**$_{Py}$-OH), 149.31 (**C**$_{Py}$-N), 115.27 (**C**$_{Py}$-H), 55.62 (O**C**H_3). ESI-HR-MS (m/z): 234.0372 $[M+Na]^+$.

Synthesis of dimethyl 4-methoxypyridine-2,6-dicarboxylate (b). This compound was prepared according to a literature procedure.[3] In a 100 mL flask, product **a** (5.02 g, 23.8 mmol) was dissolved in CH_3CN (50 mL) and potassium carbonate (4.93 g, 35.7 mmol) was added to the solution. Afterwards, methyl iodide (2.25 mL, 35.7 mmol) was carefully added to the reaction crude. The mixture was stirred and refluxed under N_2 for 24 hours. Then, the reaction mixture was cooled down to room temperature and water (50 mL) was added. The CH_3CN solvent was evaporated under reduced pressure and the aqueous phase was extracted with CH_2Cl_2 (4 x 50 mL). The organic phases were combined, dried over $MgSO_4$ and filtered. The solvent was

removed under reduced pressure to obtain compound **b** as a white solid (2.94 g, 13 mmol, 55% yield). ^{1}H-NMR ($CDCl_3$, 300 MHz, 298 K) δ, ppm: 7.80 (s, 2H, **H**$_{Py}$), 4.00 (s, 6H, CO-OC**H$_3$**), 3.96 (s, 3H, C$_{Py}$-OC**H$_3$**). ^{13}C-NMR ($CDCl_3$, 75 MHz, 298 K) δ, ppm: 167.51, 164.94, 149.46, 114.08, 55.73, 53.09. ESI-HR-MS (m/z): 248.0529 $[M+Na]^+$.

Synthesis of 4-methoxy-2,6-pyridinedicarboxylic acid (c). This compound was prepared according to a modified literature procedure.[4] In a two-necked flask, compound **b** (1.74 g, 7.73 mmol) was suspended in dry ethanol (100 mL) and stirred vigorously, followed by addition of NaOH (1.36 g, 100 mL, 34 mmol) in small portions over a period of 30 min. The mixture was stirred under N_2 for 2 hours at room temperature. After this period the solvent was removed under vacuum. The resulting residue was then brought to pH = 1 by carefully adding the necessary amount of HCl 37% and then the product was extracted with ethyl acetate (4 x 100 mL). The organic phases were combined, dried over $MgSO_4$ and concentrated until a white solid corresponding to **c** was obtained (1.49 g, 7.6 mmol, 98% yield). ^{1}H-NMR (CD_3OD, 300 MHz, 298 K) δ, ppm: 7.85 (s, 2H, **H**$_{Py}$), 3.99 (s, 3H, OC**H**$_3$). ^{13}C-NMR (CD_3OD, 75 MHz, 298 K) δ, ppm: 169.41, 163.65, 105.95, 66.24, 55.89. ESI-HR-MS (m/z): 198.0397 $[M+H]^+$, 220.0226 $[M+Na]^+$.

Synthesis of 4-methoxypyridine-2,6-dicarbonyl dichloride (d). The synthesis of this compound was performed according to a modified literature procedure.[5] A catalytic amount of DMF (ten drops) was added to compound **f** (1.98 g, 10.05 mol) followed by dropwise addition of thionyl chloride (25 mL, 341.7 mmol). After the addition, the solution was stirred and refluxed at 85 ºC under N_2 atmosphere for 6 hours. The volatile components were evaporated. Then, the resulting residue was suspended in dry toluene and the solvent was distilled so that excess $SOCl_2$ co-evaporates together with toluene. The process was repeated several times until a white-yellow crystalline solid corresponding to **d** was obtained (2.27 g, 9.7 mmol, 97% yield). ^{1}H-NMR ($CDCl_3$, 300 MHz, 298 K) δ, ppm: 7.82 (s, 2H, **H**$_{Py}$), 4.07 (s, 3H, OC**H$_3$**).

Synthesis of $H_2^{OMe}L$. On the one hand, compound **d** (855 mg, 3.67 mmol) was dissolved in toluene (83 mL) and dichloromethane (5 mL). On the other hand, (2-aminoethyl)(3-aminopropyl)methylamine (573 mg, 4.37 mmol) was also dissolved in toluene (146 mL) and dichloromethane (7 mL). Afterwards, these two solutions were transferred to two separate dropping funnels, which were both connected to a three-necked round-bottom flask containing pure toluene (25 mL). Then, both solutions were added dropwise to the flask over a period of 7 hours under a N_2 atmosphere, producing a yellow solution which was left stirring at room temperature overnight. The resulting solution was evaporated under reduced pressure, and NaOH 2 M (30 mL) was added. Finally, the solution was extracted with dichloromethane (4 x 30 mL). The organic layers were combined, dried over $MgSO_4$ and filtered. The organic solvent was removed under reduced pressure to obtain a white solid. The solid was purified by column chromatography over silica using a 90:10 dichloromethane/methanol solution as eluent to obtain a white solid corresponding to **$H_2^{OMe}L$** (220.8 mg, 0.76 mmol, 21% yield). ^{1}H-NMR ($CDCl_3$, 400

MHz, 298 K) δ, ppm: 10.20 (br s, 1H, N**H**), 9.73 (br s, 1H, N**H**), 7.59 (d, *J* = 2 Hz, 1H, **H**$_{Py}$), 7.53 (d, *J* = 2 Hz, 1H, **H**$_{Py}$), 3.96 (s, 3H, OC**H**$_3$), 3.88 (m, 1H, C**H**$_2$NH), 3.62 (m, 1H, C**H**$_2$NH), 3.52 (m, 1H, C**H**$_2$NH), 3.32 (m, 1H, C**H**$_2$NH), 2.97 (m, 1H, C**H**$_2$Me), 2.85 (m, 2H, C**H**$_2$NMe), 2.48 (m, 1H, C**H**$_2$NMe), 2.22 (s, 3H, C**H**$_3$), 2.13 (m, 1H, CH$_2$C**H**$_2$CH$_2$), 1.86 (m, 1H, CH$_2$C**H**$_2$CH$_2$). ^{13}C-NMR (CDCl$_3$, 75 MHz, 298 K) δ, ppm: 170.21, 165.78, 163.45, 150.34, 149.05, 108.71, 108.65, 58.86, 56.37, 40.80, 37.80, 37.30, 23.96. ESI-HR-MS (m/z): 293.1608 [M+H]$^+$, 315.1428 [M+Na]$^+$.

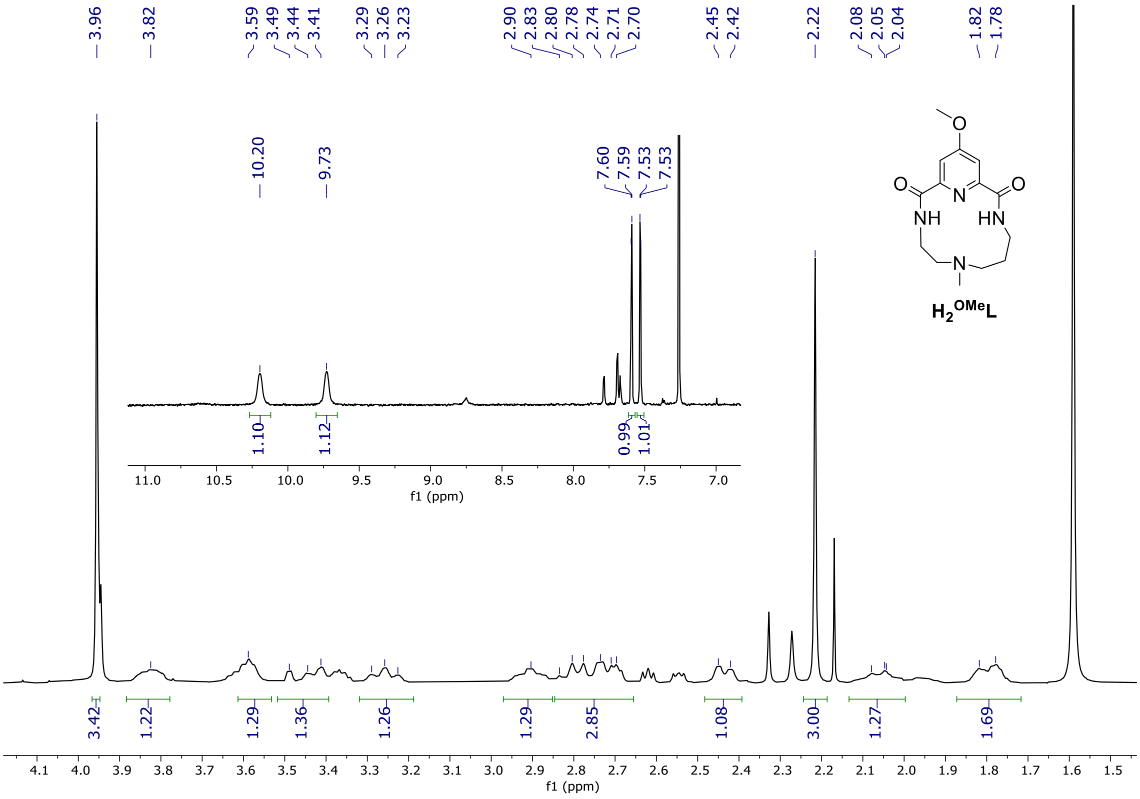

Figure S1. ^{1}H-NMR spectrum of **H$_2$^{OMe}L** in CDCl$_3$ at 298 K (400 MHz).

2.2. Synthesis of $H_2{}^{CF3}L$

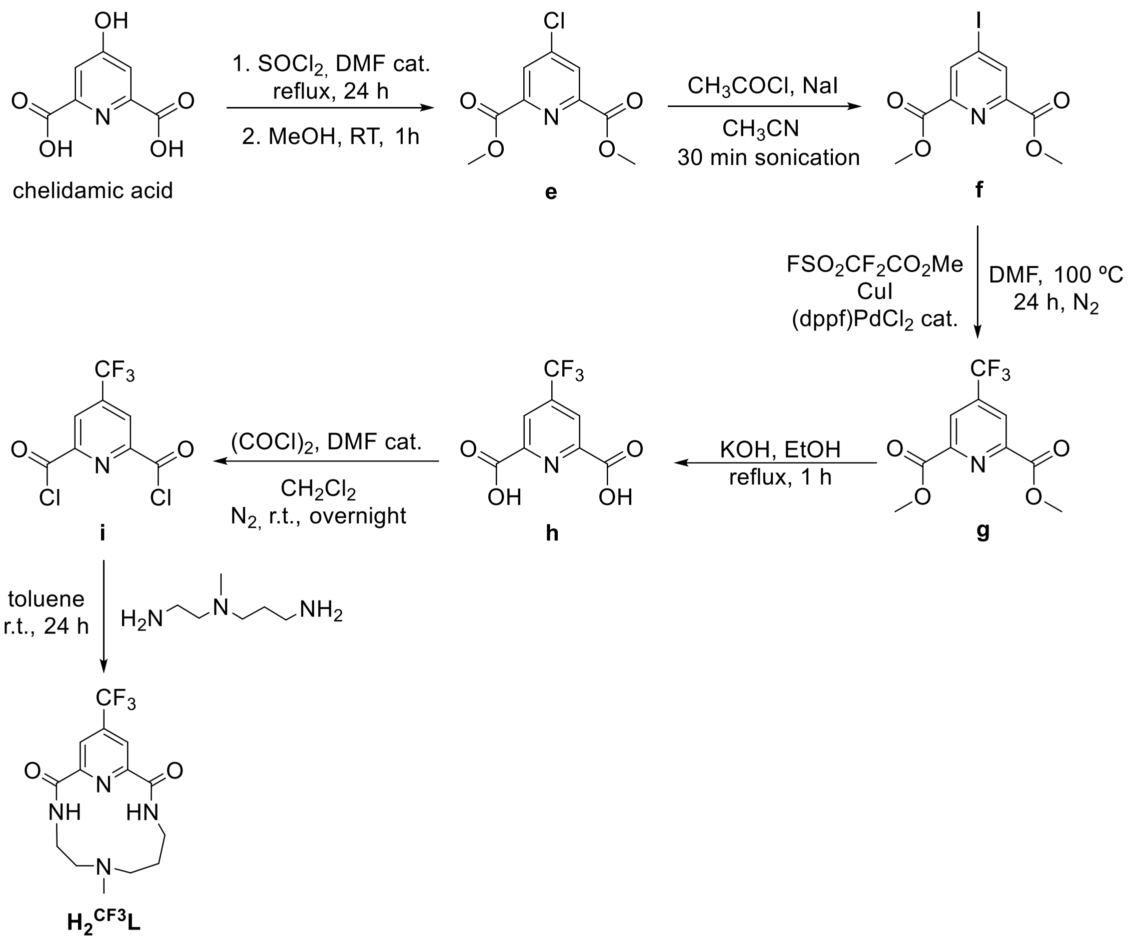

Scheme S2. Synthetic route for the synthesis of **$H_2{}^{CF3}L$**.

Synthesis of dimethyl 4-chloropyridine-2,6-dicarboxylate (e). This compound was synthesized following a modified literature procedure.[6] In a 100 mL round-bottomed flask chelidamic acid hydrate (6.2 g, 30.6 mmol), thionyl chloride (20 mL, 275.7 mmol) and one drop DMF were mixed. The resulting white solution was stirred at 100 °C overnight. Afterwards, the resulting red solution was distilled at 100 °C to remove the excess of thionyl chloride. Afterwards, methanol (24 mL) was slowly added at 0 °C under stirring, and the resulting mixture was further stirred for 1 hour at 0 °C. Then, methanol was evaporated under reduced pressure to dryness and a yellowish solid was obtained. The solid was dissolved in chloroform (40 mL) and extracted with water (2 x 40 mL) and brine (2 x 40 mL). The organic phase was dried with anhydrous $MgSO_4$, filtered and the solvent was removed under reduced pressure giving a yellowish solid corresponding to **e** (4.8 g, 21.0 mmol, 68%). ^{1}H-NMR ($CDCl_3$, 400 MHz, 298 K) δ, ppm: 8.32 (s, 2H, H_{Py}), 4.06 (s, 6H, OC**H**$_3$). ^{13}C-NMR ($CDCl_3$, 75 MHz, 298 K) δ, ppm: 164.11 (**C**=O), 149.39

($\mathbf{C}_{Py}$-N), 146.84 (C_{Py}-Cl), 128.33 (C_{Py}-H), 53.52 (O**C**H_3). ESI-MS (m/z): 230.0 $[M+H]^+$, 252.0 $[M+Na]^+$.

Synthesis of dimethyl 4-iodopyridine-2,6-dicarboxylate (f). Compound **f** was synthesized following a modified literature procedure.[7-8] Under an inert atmosphere, compound **e** (3.1 g, 13.5 mmol), NaI (40.4 g, 269.6 mmol) and anhydrous CH_3CN (120 mL) were mixed into a 500 mL two-necked Schlenk flask. Acetyl chloride (3 mL, 40.1 mmol) was then carefully added under a N_2 atmosphere and stirring at 0 °C. The resulting mixture was sonicated for 30 min under a N_2 atmosphere, checking that the bath temperature did not exceed 50 °C. Afterwards, the resulting crude was cooled at 0 °C and a red solution with a white precipitate was obtained. At this point, CH_2Cl_2 (120 mL) was added and the mixture was extracted with a saturated aqueous solution of Na_2CO_3 (60 mL). The organic phase was washed with a saturated solution of $Na_2S_2O_3$ (100 mL) and water (2 x 100 mL). The organic phase was dried with anhydrous $MgSO_4$, filtered and the solvent was evaporated under reduced pressure giving a yellowish-white solid. The solid was purified by recrystallization with hot methanol and pure product **f** was obtained as a white solid (3.4 g, 10.6 mmol, 78%). ^{1}H-NMR ($CDCl_3$, 400 MHz, 298 K) δ, ppm: 8.69 (s, 2H, $\mathbf{H}_{Py}$), 4.05 (s, 6H, OC$\mathbf{H}_3$). ^{13}C-NMR ($CDCl_3$, 75 MHz, 298 K) δ, ppm: 163.87 (**C**=O), 148.26 ($\mathbf{C}_{Py}$-N), 137.16 ($\mathbf{C}_{Py}$-H), 107.02 ($\mathbf{C}_{Py}$-I), 53.48 (O**C**H_3). ESI-MS (m/z): 321.90 $[M+H]^+$, 359.90 $[M+K]^+$.

Synthesis of dimethyl 4-trifluoromethylpyridine-2,6-dicarboxylate (g). Compound **g** was synthesized following a literature procedure.[9] A 250 mL two-necked Schlenk flask containing product **f** (2.5 g, 7.8 mmol) was equipped with a reflux condenser and connected to a Schlenk line. $[PdCl_2(dppf)]$ (0.28 g, 0.40 mmol) and CuI (1.4 g, 7.4 mmol) were added under a nitrogen atmosphere. Afterwards, anhydrous DMF (120 mL) and a solution of $FSO_2CF_2CO_2CH_3$ (5 mL, 39.3 mmol) in anhydrous DMF (16 mL) were added under stirring. The resulting mixture was stirred at 100 °C overnight, under a N_2 atmosphere. After cooling to room temperature, dichloromethane (250 mL) was added and the solution was filtered off. The resulting dark brown filtrate was extracted with water (2 x 250 mL), a 3.25 M solution of NaCl (2 x 250 mL) and brine (2 x 250 mL). The organic phase was dried with anhydrous $MgSO_4$, filtered and the solvent was evaporated under reduced pressure, affording a black solid. The residue was purified by column chromatography over silica using AcOEt:hexane 3:7 as eluent. Product **g** was obtained as a crystalline white solid (1.4 g, 5.3 mmol, 68%). ^{1}H-NMR ($CDCl_3$, 400 MHz, 298 K) δ, ppm: 8.55 (s, 2H, H_{Py}), 4.10 (s, 6H, OC$\mathbf{H}_3$). ^{13}C-NMR ($CDCl_3$, 75 MHz, 298 K) δ, ppm: 163.90 (**C**=O), 149.66 ($\mathbf{C}_{Py}$-CF_3), 141.12 ($\mathbf{C}_{Py}$-N), 123.76 ($\mathbf{C}_{Py}$-H), 120.14 (**C**F_3), 53.59 (O**C**H_3). ^{19}F-NMR ($CDCl_3$, 282 MHz, 298K) δ, ppm: - 64.70 (C$\mathbf{F}_3$). ESI-HR-MS (m/z): 263.90 $[M+H]^+$, 285.90 $[M+Na]^+$.

Synthesis of 4-trifluoromethyl-2,6-pyridinedicarboxylic acid (h). A solution of NaOH (0.41 g, 10.3 mmol) in water (47 mL) was added into a 100 mL round-bottomed flask containing product **c** (0.45 g, 1.7 mmol). The resulting mixture was stirred for 1 hour at 100 °C. Then, it was cooled to room temperature and acidified to pH = 1 by dropwise addition of HCl 37% at 0 °C under

stirring. Upon acidification, a white precipitate was obtained, which was filtered under vacuum, washed with water and dried. Meanwhile, the filtrate solution was extracted with dichloromethane (3 x 50 mL). The organic layer was dried with anhydrous magnesium sulfate, filtered and the solvent was removed to dryness affording a white solid. Both white solids corresponded to the pure product **h** (0.36 g, 1.5 mmol, 90%). ^{1}H-NMR (CD_3OD, 400 MHz, 298 K) δ, ppm: 8.57 (s, 2H). ^{13}C-NMR (CD_3OD, 75 MHz, 298 K) δ, ppm: 164.53 (**C**=O), 149.51 (**C**$_{Py}$-CF_3), 142.6 (**C**$_{Py}$-N), 130.92 (**C**$_{Py}$-H), 127.67 (**C**F_3). ^{19}F-NMR ($CDCl_3$, 282 MHz, 298 K) δ, ppm: - 67.26 (C**F$_3$**). ESI-HR-MS (m/z): 235.90 $[M+H]^+$, 257.90 $[M+Na]^+$.

Synthesis of 4-trifluoromethylpyridine-2,6-dicarbonyl dichloride (i). In a 50 mL round-bottomed flask containing product **h** (1.12 g, 4.8 mmol), dry CH_2Cl_2 (60 mL) and a drop of anhydrous DMF were added under a N_2 atmosphere and stirring. The solution was cooled to 0 °C in an ice bath and then oxalyl chloride (1.2 mL, 14.3 mmol) was carefully added. The mixture was stirred overnight at room temperature under a N_2 atmosphere. Afterwards, the solvent was evaporated under reduced pressure to dryness and the resulting solid was extracted with anhydrous toluene (3 x 10 mL). The liquid was decanted, and the solvent was removed under reduced pressure obtaining product **i** as a yellowish oil that slowly solidified (1.19 g, 4.4 mmol, 92 %). This compound was used directly in the next step without further purification. ^{1}H-NMR ($CDCl_3$, 400 MHz, 298 K) δ, ppm: 8.56 (s, 2H). ^{13}C-NMR ($CDCl_3$, 75 MHz, 298 K) δ, ppm: 168.55 (**C**=O), 159.09 (**C**$_{Py}$-N), 150.49 (**C**$_{Py}$-CF_3), 136.84 (**C**$_{Py}$-H), 124.47 (**C**F_3). ^{19}F-NMR ($CDCl_3$, 282 MHz, 298 K) δ, ppm: - 64.45 (C**F$_3$**).

Synthesis of $H_2{}^{CF3}L$. On the one hand, compound **i** (1.19 g, 4.37 mmol) was dissolved in toluene (100 mL). On the other hand, (2-aminoethyl)(3-aminopropyl)methylamine (0.65 g, 4.95 mol) was dissolved in toluene (174 mL) and dichloromethane (8 mL). Afterwards, the two solutions were transferred to two separate dropping funnels, which were connected to a three-necked round-bottom flask containing 25 mL of toluene. Then, both solutions were added dropwise into the flask over a period of 7 hours under a N_2 atmosphere. After the addition, the resulting yellow solution was stirred at room temperature overnight. The final mixture was evaporated under reduced pressure, and NaOH 2 M (35 mL) was added. Finally, the solution was extracted with dichloromethane (4 x 35 mL). The organic layers were combined and dried with $MgSO_4$ and filtered. The organic solvent was removed under reduced pressure to obtain a brown oil. The oil was purified by column chromatography over silica using a 90:10:1 dichloromethane/methanol/ammonia solution as eluent to obtain a white solid corresponding to **$H_2{}^{CF3}L$** (63.5 mg, 0.20 mmol, 5% yield). ^{1}H-NMR ($CDCl_3$, 400 MHz, 298 K) δ, ppm: 10.11 (br s, 1H, N**H**), 9.58 (br s, 1H, N**H**), 8.32 (s, 1H, **H**$_{Py}$), 8.27 (s, 1H, **H**$_{Py}$), 3.90 (m, 1H, C**H$_2$**NH), 3.61 (m, 1H, C**H$_2$**NH), 3.50 (m, 1H, C**H$_2$**NH), 3.33 (m, 1H, C**H$_2$**NH), 2.97 (m, 1H, C**H$_2$**Me), 2.83 (m, 2H, C**H$_2$**NMe), 2.51 (m, 1H, C**H$_2$**NMe), 2.21 (s, 3H, C**H$_3$**), 2.15 (m, 1H, CH_2C**H$_2$**CH_2), 1.87 (m, 1H, CH_2C**H$_2$**CH_2). ^{19}F-NMR ($CDCl_3$, 282 MHz, 298 K) δ, ppm: - 64.37 (C**F$_3$**). ESI-MS (m/z): 331.20 $[M+H]^+$, 331.20 $[M+Na]^+$.

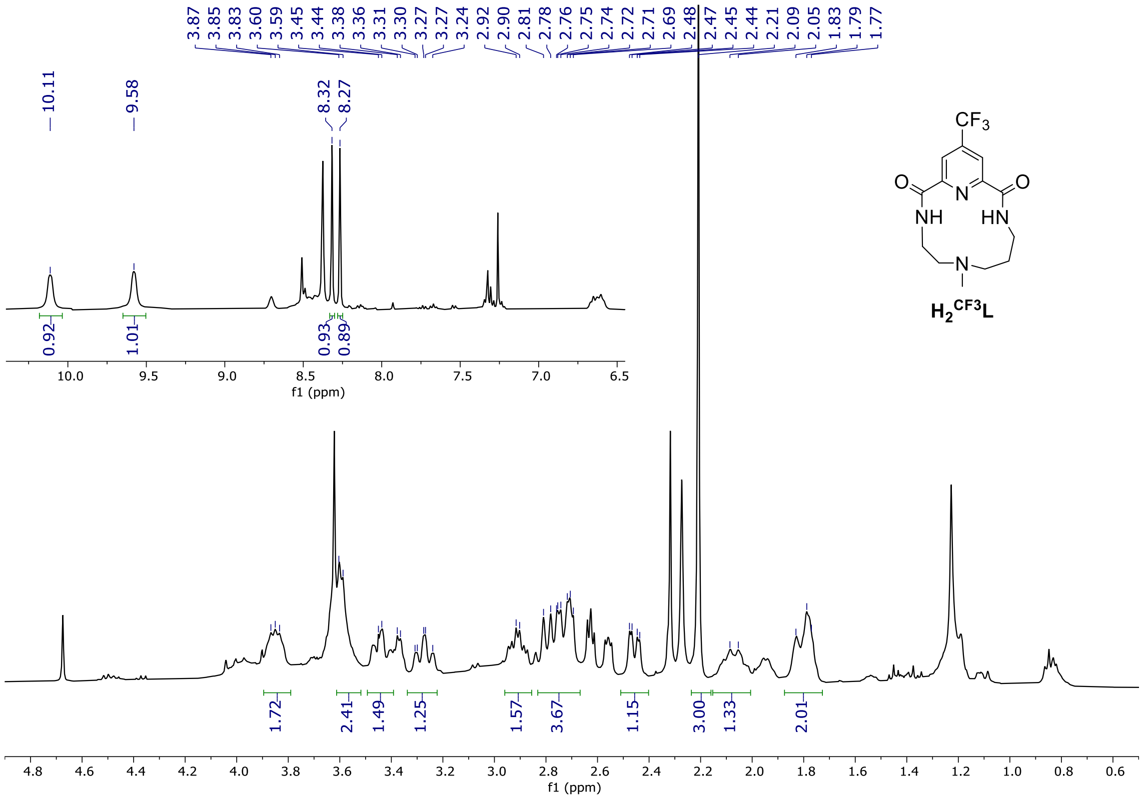

Figure S2. ^{1}H-NMR spectrum of **H_2^{CF3}L** in $CDCl_3$ at 298 K (400 MHz).

3. Synthesis and characterization of nickel(II) complexes

3.1. Synthesis of [Ni(^{OMe}L)] and [Ni(^{CF3}L)]

Synthesis of [Ni(^{OMe}L)] (^{OMe}1). In the glovebox, **H_2^{OMe}L** (45.7 mg, 0.16 mmol) was dissolved in anhydrous acetonitrile (0.5 mL). Afterwards, a solution of [$Ni^{II}(CF_3SO_3)_2(CH_3CN)_3$] (69.3 mg, 0.16 mmol) in anhydrous acetonitrile (1 mL) was added dropwise and the mixture was stirred vigorously. The addition of NaH (10.7 mg, 0.45 mmol) dissolved in anhydrous acetonitrile (1 mL) caused a color change, first obtaining a pale green solution that evolved to an orange solution. The reaction mixture was stirred overnight, and the solvent was removed. The resulting residue was dissolved with the minimal amount of methanol. Finally, the mixture was filtered through Celite® and concentrated. Slow diethyl ether diffusion over the resulting solution afforded in a few days yellow laminar crystals corresponding to **[Ni(^{OMe}L)]** (42.7 mg, 0.09 mmol, 56% yield). ^{1}H-NMR (CD_3OD, 400 MHz, 298 K) δ, ppm: 7.10 (d, J = 2.04 Hz, 1H, **H_B**), 7.05 (d, J = 2.04 Hz, 1H, **H_A**), 3.97 (s, 3H, OC**H_3**), 3.51-3.43 (m. 1H, **H_D**), 3.38-3.34 (m, 1H, **H_E**), 3.28-3.24 (m, 2H, **H_C**), 3.02-2.95 (m, 1H, **H_G**), 2.90-2.82 (m, 1H, **H_E**), 2,80-2.76 (m. 1H, **H_D**) 2.74 (s, 3H, C**H_3**), 2.55-2.50 (m, 1H, **H_G**), 1.94-1.79 (m, 2H, **H_F**). ^{13}C-NMR (CD_3OD, 100 MHz, 298 K) δ, ppm: 172.38 (**C_1**), 170.85 (**C_7**), 167.73 (**C_6**), 156.15 (**$C_{3/5}$**), 155.15 (**$C_{3/5}$**), 109.29 (**C_2**), 108.91 (**C_4**), 66.60 (**C_9**), 59.61 (**C_{12}**), 57.42 (O**C**H_3), 43.09 (**C_8**), 42.44 (**C_{10}**), 42.21 (**C**H_3), 27.55 (**C_{11}**). ESI-HR-MS (m/z): 371.0636 $[M+Na]^+$. UV-vis (CH_3CN, -30 ºC) λ_{max}, nm (ε, $M^{-1}cm^{-1}$): 340 (3000), 480 (481). Elemental analysis: calculated for **[Ni(^{OMe}L)]**·1.5$NaCF_3SO_3$·0.5CH_3CN·0.5Et_2O 33.43 %C, 3.72 %H, 9.48 %N; experimental 33.46 %C, 4.16 %H, 9.27 %N.

Synthesis of [Ni(^{CF3}L)] (^{CF3}1). In the glovebox, **H_2^{CF3}L** (63.5 mg, 0.19 mmol) was dissolved in anhydrous acetonitrile (0.5 mL). After that, a solution of [$Ni^{II}(CF_3SO_3)_2(CH_3CN)_3$] (84.4 mg, 0.19 mmol) in anhydrous acetonitrile (1 mL) was added dropwise and the mixture was stirred vigorously. After a few seconds, the pale white solution turns brown. The addition of NaH (11.5 mg, 0.48 mmol) caused a further color change to deep red. The reaction mixture was stirred overnight, and the solvent was removed. The resulting residue was dissolved with the minimal amount of methanol. Finally, the mixture was filtered through Celite® and concentrated. Slow diethyl ether diffusion over the resulting solution afforded in a few days dark red crystals corresponding to **[Ni(^{CF3}L)]** (13 mg, 0.03 mmol, 14% yield). ^{1}H-NMR (CD_3OD, 400 MHz, 298 K) δ, ppm: 7.83 (br s, 1H, **H_B**), 7.76 (br s, 1H, **H_A**), 3.55-3.47 (m, 1H, **H_D**), 3.44-3.42 (m, 1H, **H_E**), 3.40-3.38 (m, 2H, **H_C**), 3.03-2.97 (m, 1H, **H_G**), 2.90-2.88 (m, 1H, **H_E**), 2.86-2.83 (m. 1H, **H_D**), 2.77 (s, 3H, C**H_3**), 2.58-2.53 (m, 1H, **H_G**), 1.98-1.84 (m, 2H, **H_F**).^{13}C-NMR (CD_3OD, 100 MHz, 298 K) δ, ppm: 169.71 (**C_7**), 166.56 (**C_6**), 156.16 (**$C_{3/5}$**), 155.21 (**$C_{3/5}$**), 121.76 (q, J = 316,4 Hz, **C**F_3), 119.27 (**C_2**), 119.15 (**C_4**), 66.69 (**C_9**), 59.54 (**C_{12}**), 43.19 (**C_8**), 42.34 (**C_{10}**), 42.27 (**C**H_3), 27.49 (**C_{11}**). ^{19}F-NMR (CD_3CN, 377 MHz, 298 K) δ, ppm: -67.11 (**C**F_3). ESI-MS (m/z): 409.0393 $[M+Na]^+$, 427.0498 $[M+Na+H_2O]^+$, 795.0874 $[2M+Na]^+$. UV-vis (CH_3CN, -30 ºC) λ_{max}, nm (ε, $M^{-1}cm^{-1}$): 376 nm (8333), 522 nm (687). Elemental analysis: calculated for **[Ni(^{CF3}L)]**·0.75$NaCF_3SO_3$·0.5Et_2O 36.37 %C, 3.64 %H, 10.13 %N; experimental 36.25 %C, 3.29 %H, 10.24 %N.

3.2 ^{1}H-NMR and UV-vis spectra of [Ni(^{OMe}L)] and [Ni(^{CF3}L)]

The most relevant couplings for the assignment of the signals of ^{OMe}**1** have been highlighted in their corresponding spectra (Figures S3-S11). The observed couplings that were crucial for the assignment are:

- ^{1}H-^{13}C HMBC (Figure S10): coupling of methyl hydrogens (CH_3) with carbons 9/12.
- ^{1}H-^{1}H COSY (Figure S6): couplings of hydrogens G with F, D with C, F with G/E, C with D, and E with F. These assignments were also analyzed by ^{1}H-^{1}H TOCSY (Figure S8), confirming which carbons are part of the three-carbon aliphatic chain (E, F, and G) and which ones are part of the two-carbon aliphatic chain (C, and D).
- ^{1}H-^{13}C HMBC: couplings of hydrogen C with carbon 6, carbon 6 with hydrogen A, hydrogen E with carbon 7, and carbon 7 with hydrogen B (Figure S10). Thanks to the ligand asymmetry, the two carbonyl carbons and pyridine hydrogens A and B could be distinguished (Figure S11). However, four-bond couplings between hydrogen A and carbon 5, and hydrogen B and carbon 3 were not detected. Thus, quaternary carbons 3 and 5 are detected as separate signals but cannot be assigned.

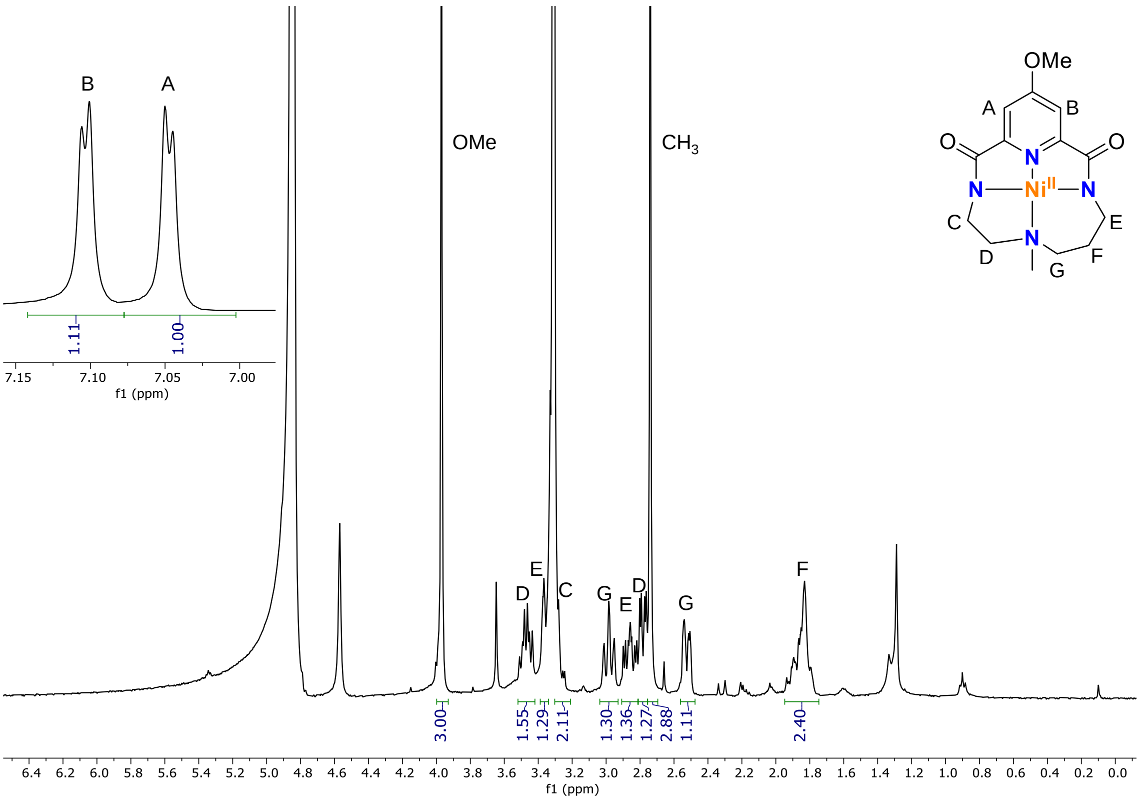

Figure S3. ^{1}H-NMR spectrum of ^{OMe}**1** in CD_3OD at 298 K (400 MHz).

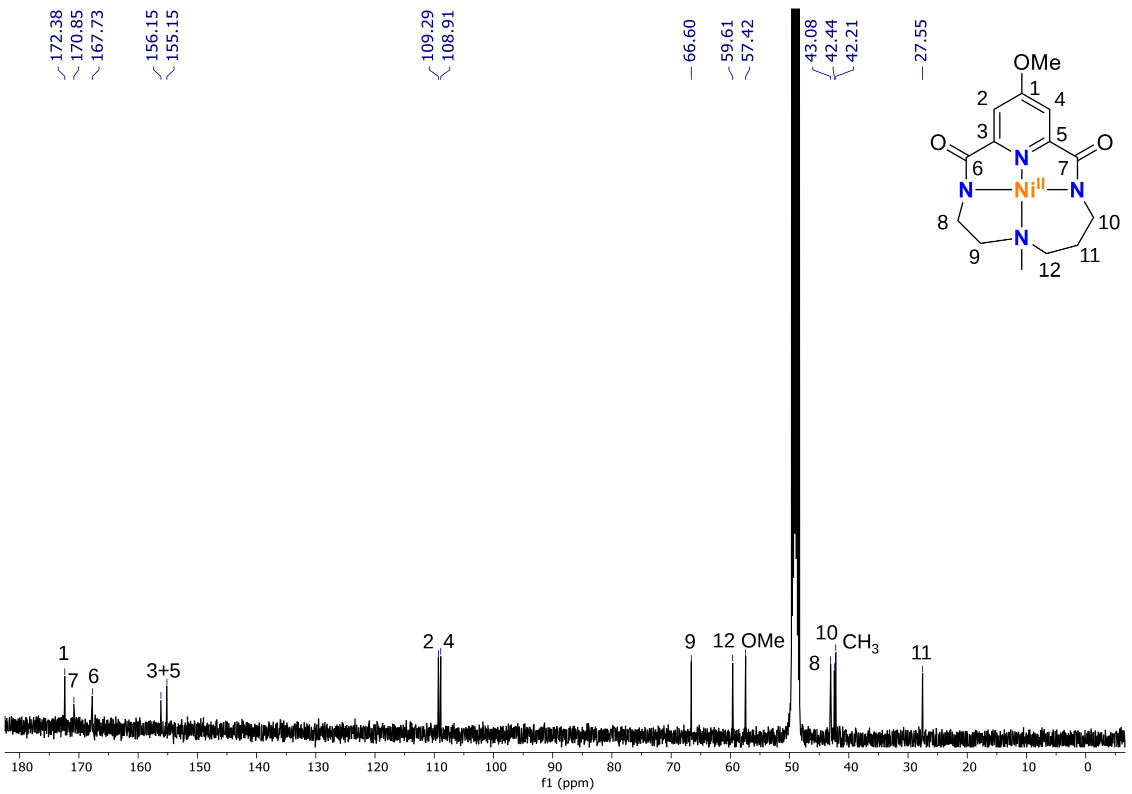

Figure S4. ^{13}C-NMR spectum of $^{\mathbf{OMe}}\mathbf{1}$ in CD_3OD at 298 K (100 MHz).

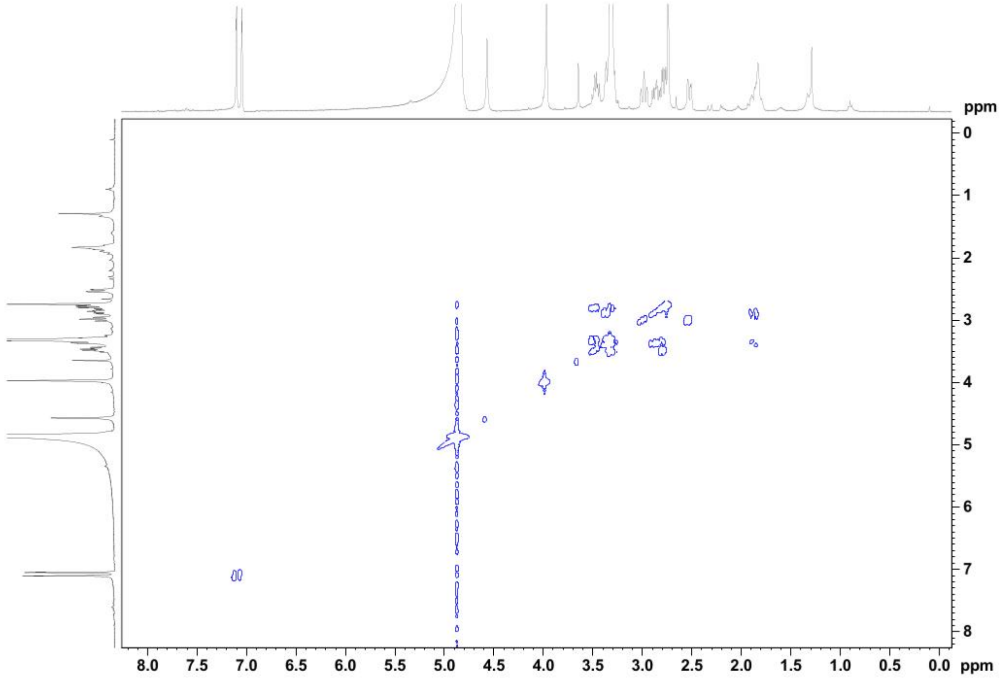

Figure S5. ^{1}H-^{1}H COSY NMR spectrum of $^{\mathbf{OMe}}\mathbf{1}$ in CD_3OD at 298 K (400 MHz).

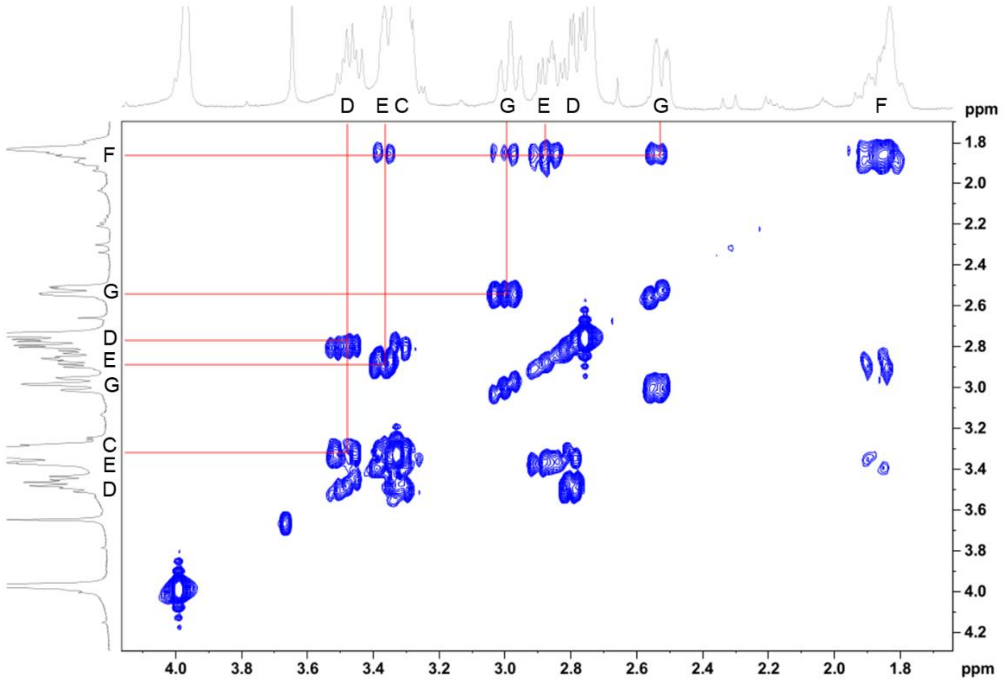

Figure S6. Aliphatic region of the ^1H-^1H COSY NMR spectrum of $^{\mathbf{OMe}}\mathbf{1}$ in CD_3OD at 298 K (400 MHz).

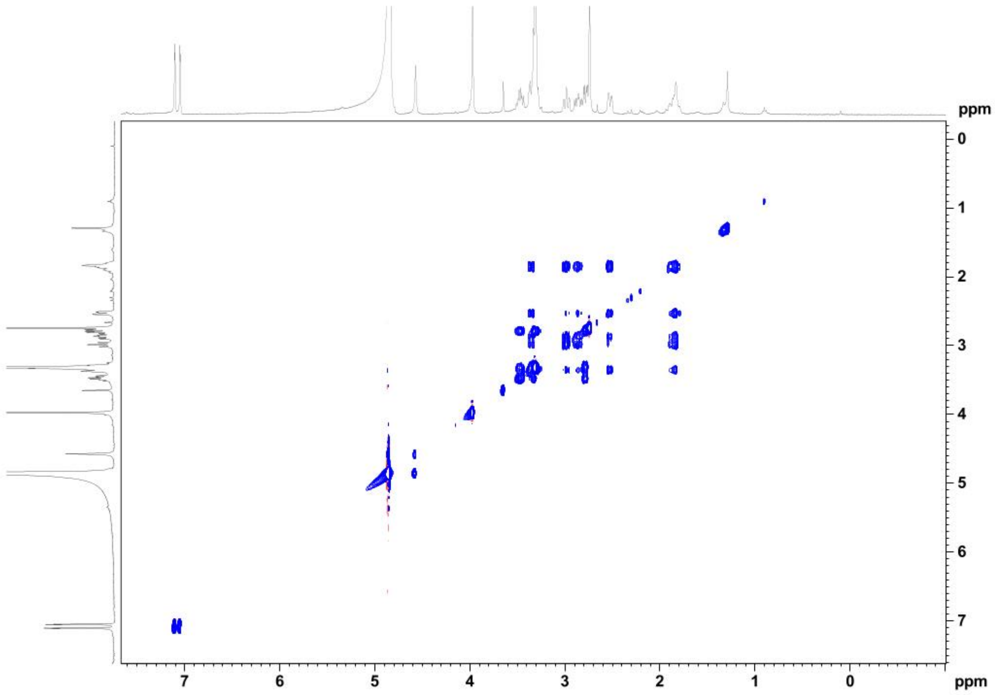

Figure S7. ^1H-^1H TOCSY NMR spectrum of $^{\mathbf{OMe}}\mathbf{1}$ in CD_3OD at 298 K (400 MHz).

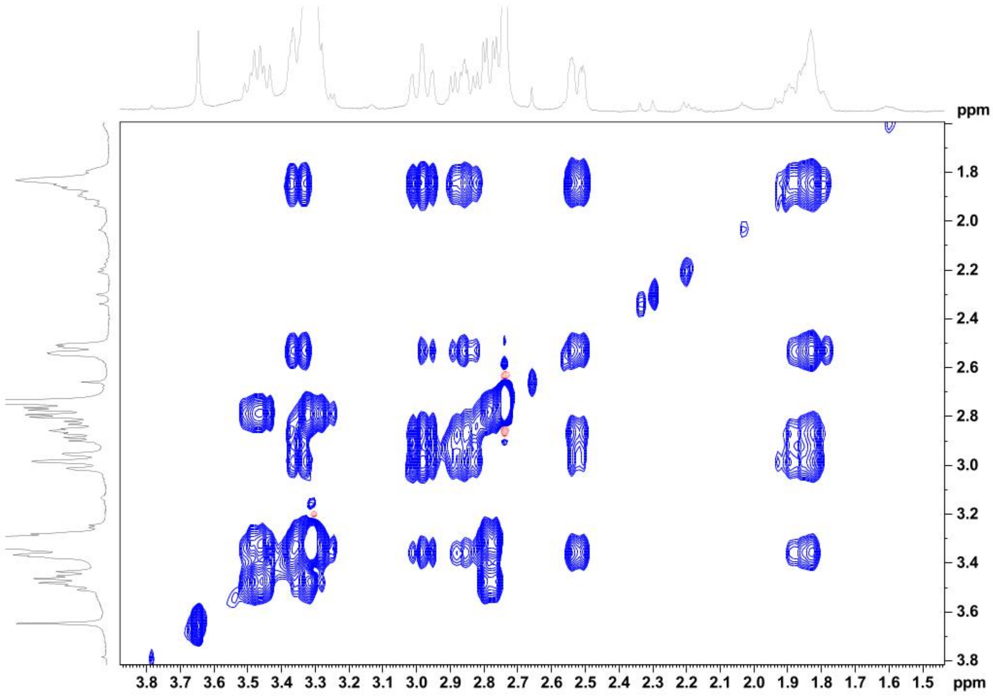

Figure S8. Aliphatic region of the ^{1}H-^{1}H TOCSY NMR spectrum of $^{\mathbf{OMe}}\mathbf{1}$ in CD_3OD at 298 K (400 MHz).

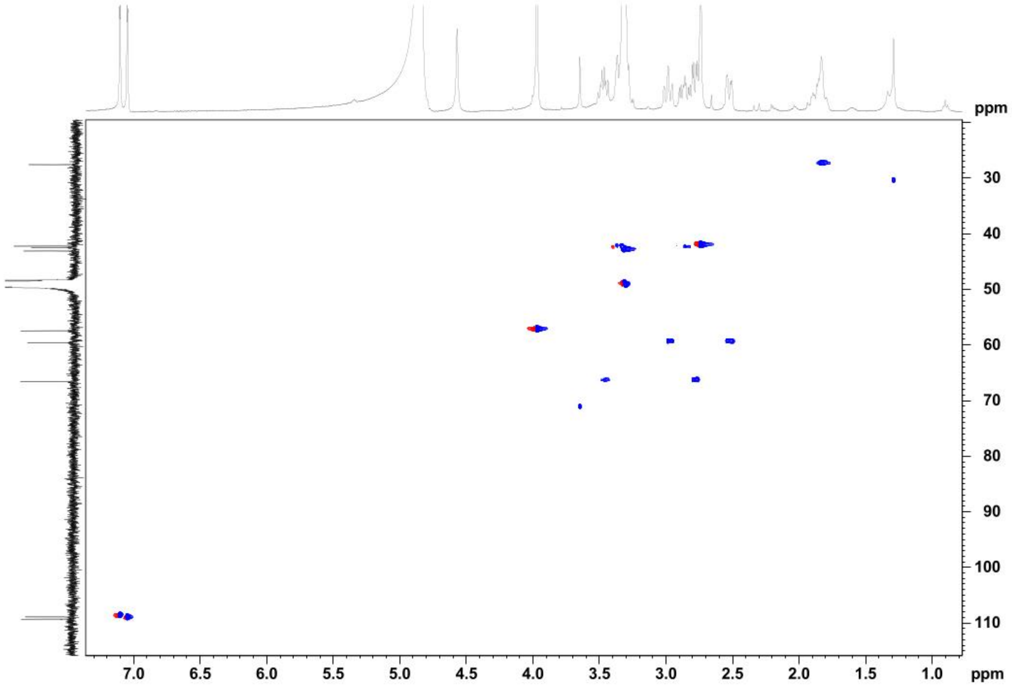

Figure S9. ^{1}H-^{13}C HSQC NMR spectrum of $^{\mathbf{OMe}}\mathbf{1}$ in CD_3OD at 298 K (400 MHz).

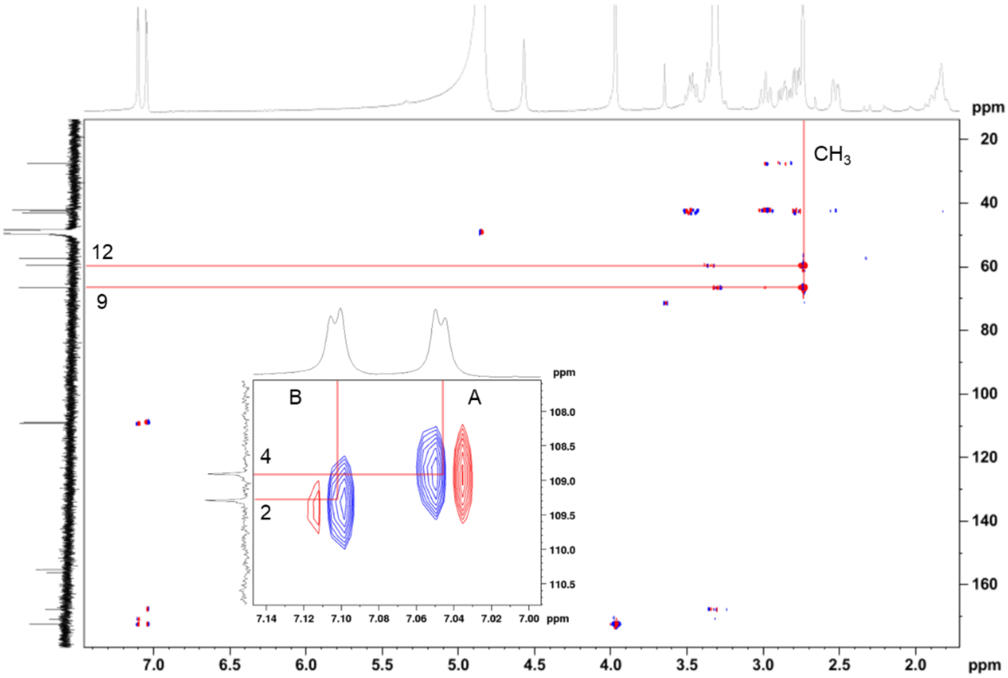

Figure S10. ^{1}H-^{13}C HMBC NMR spectrum of ^{OMe}**1** in CD_3OD at 298 K (400 MHz).

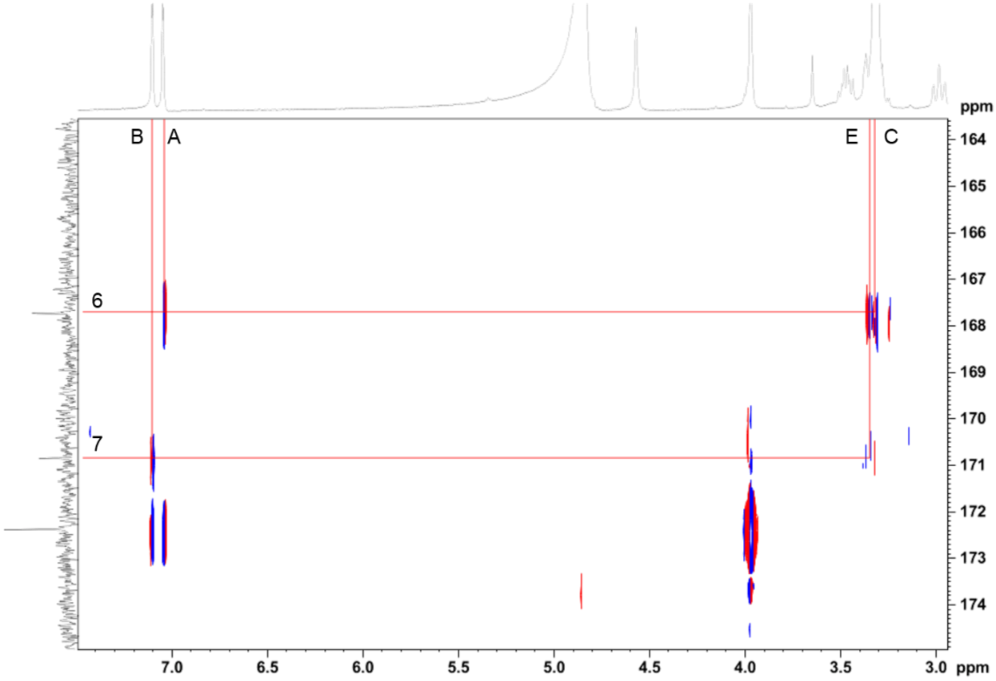

Figure S11. ^{1}H-^{13}C HMBC NMR spectrum of ^{OMe}**1** corresponding to the carbonyl signals in CD_3OD at 298 K (400 MHz).

The most relevant couplings for the assignment of the signals of $^{\mathrm{CF3}}$**1** have been highlighted in their corresponding spectra (Figures S12-S21). The observed couplings that were crucial for the assignment are:

- ^{1}H-^{13}C HMBC (Figure S20): coupling of methyl hydrogens (CH_3) with carbons 9/12.
- ^{1}H-^{1}H COSY (Figure S16): couplings of hydrogens G with F, D with C, F with G/E, C with D, and E with F. These assignments were also analyzed by ^{1}H-^{1}H TOCSY (Figure S18), confirming which carbons are part of the three-carbon aliphatic chain (E, F, and G) and which ones are part of the two-carbon aliphatic chain (C, and D).
- ^{1}H-^{13}C HMBC (Figure S21): couplings hydrogen C with carbon 6, carbon 6 with hydrogen A, hydrogen E with carbon 7, and carbon 7 with hydrogen B. Due to the ligand asymmetry, the two carbonyl carbons and hydrogens A and B could be distinguished. Four-bond couplings between hydrogen A and carbon 5, and hydrogen B and carbon 3 were detected. However, these couplings appear very close one to the other. Thus, carbons 3 and 5 cannot be unequivocally assigned.

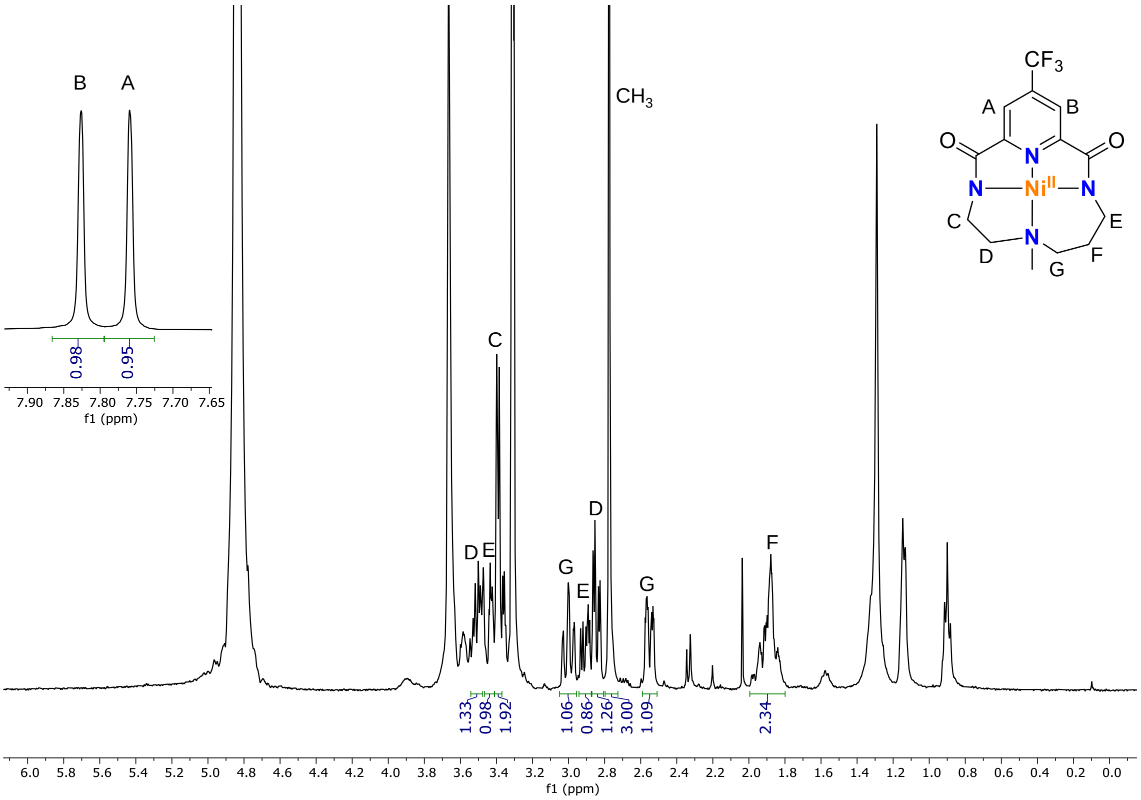

Figure S12. ^{1}H-NMR spectum of $^{\mathrm{CF3}}$**1** in CD_3OD at 298 K (400 MHz).

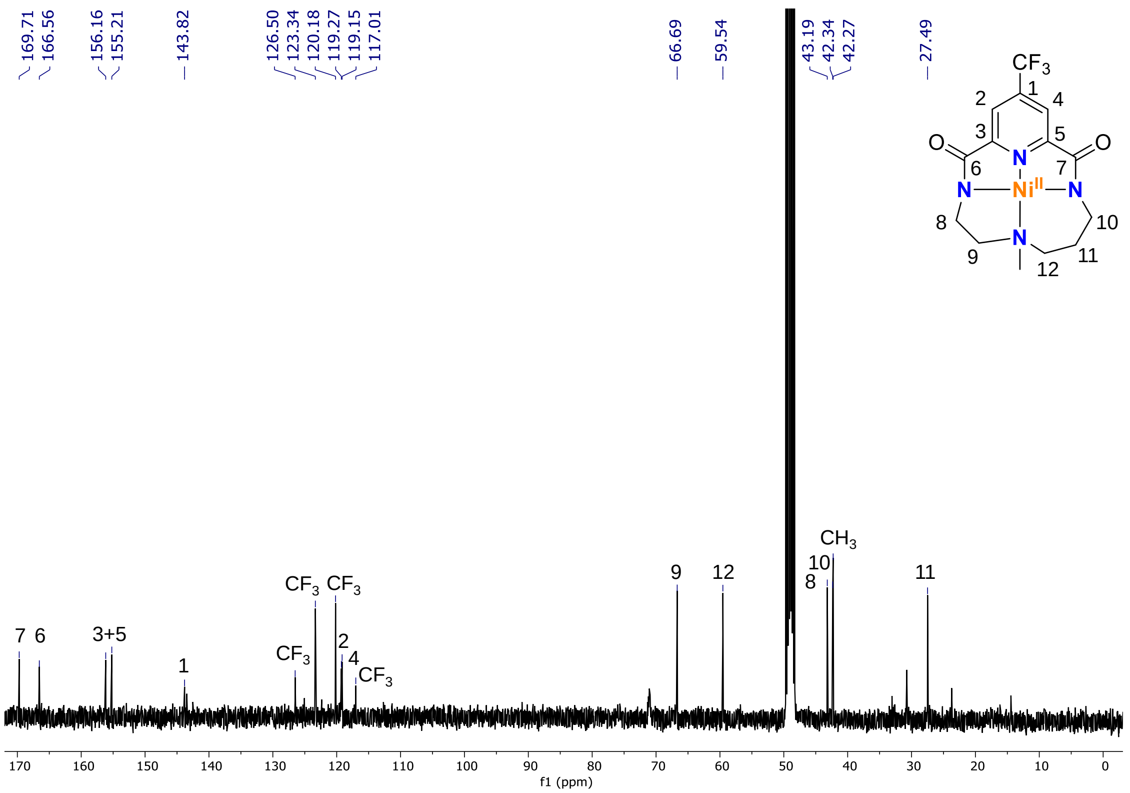

Figure S13. ^{13}C-NMR spectum of ^{CF3}**1** in CD_3OD at 298 K (100 MHz).

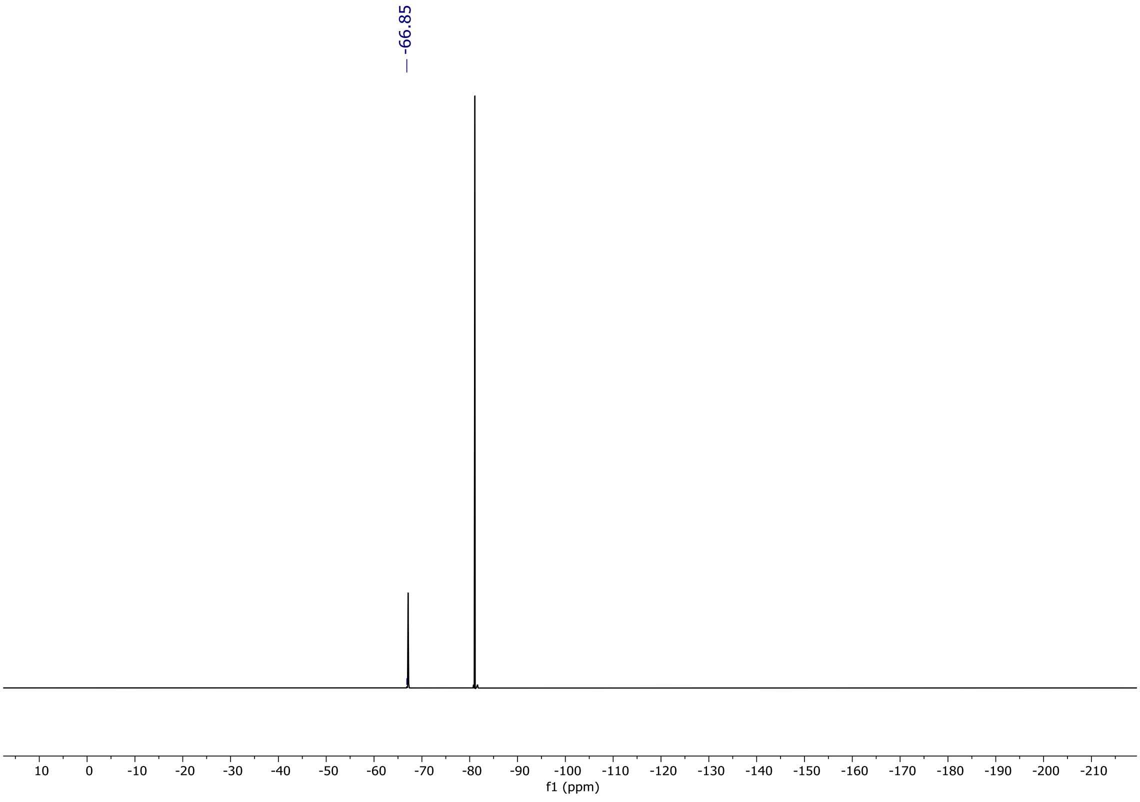

Figure S14. ^{19}F-NMR spectum of ^{CF3}**1** in CD_3OD at 298 K (377 MHz).

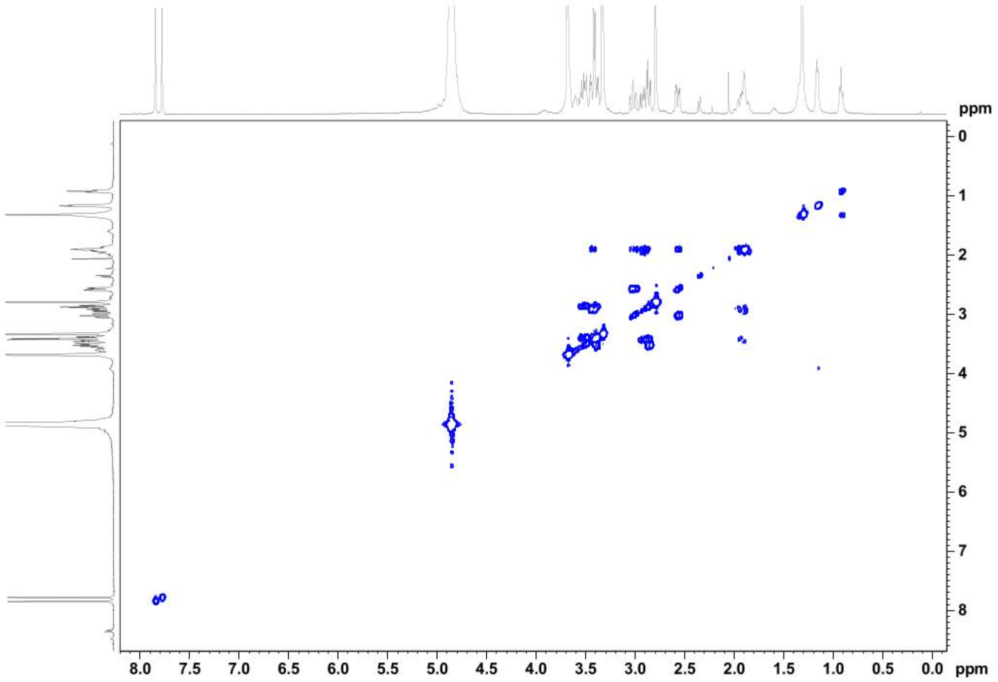

Figure S15. ^{1}H-^{1}H COSY NMR spectrum of $^{\mathbf{CF3}}$**1** in CD_3OD at 298 K (400 MHz).

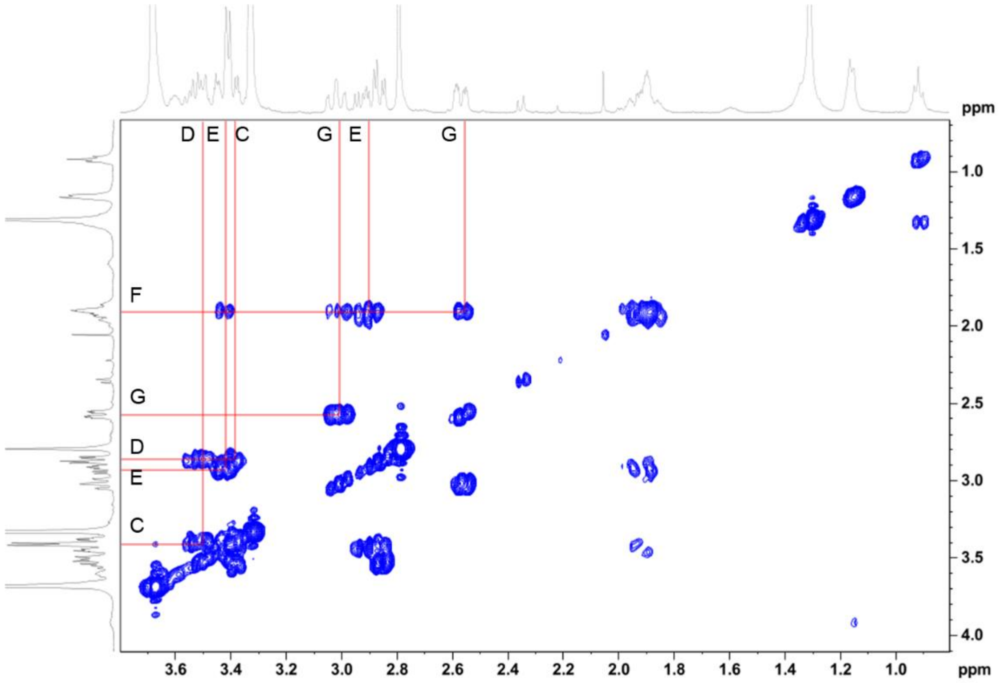

Figure S16. Aliphatic region of the ^{1}H-^{1}H COSY NMR spectrum of $^{\mathbf{CF3}}$**1** in CD_3OD at 298 K (400 MHz).

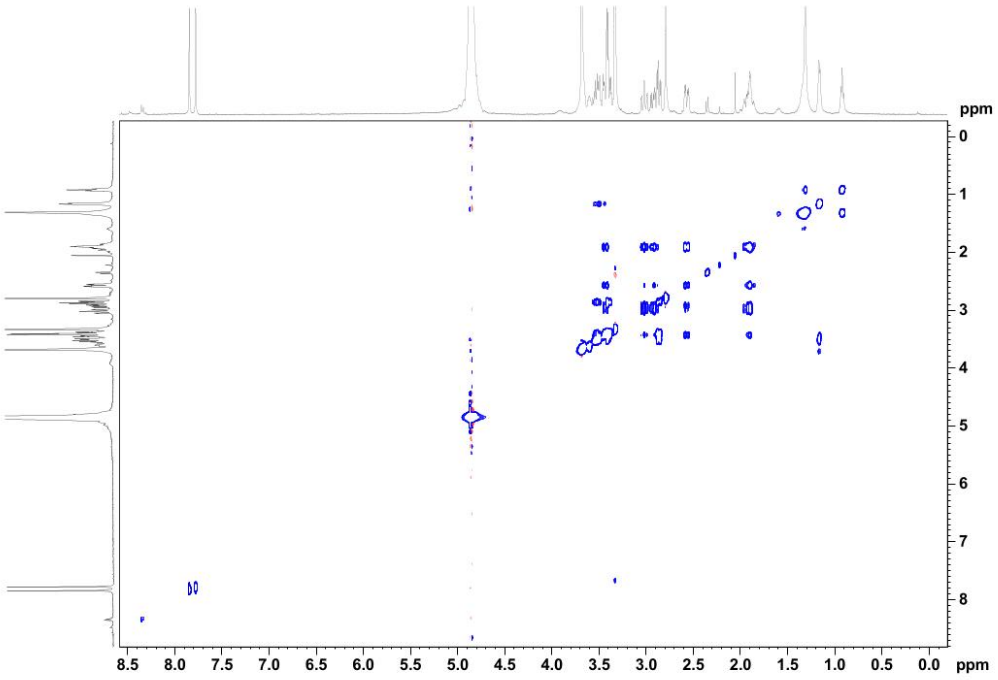

Figure S17. ^{1}H-^{1}H TOCSY NMR spectrum of ^{CF3}**1** in CD_3OD at 298 K (400 MHz).

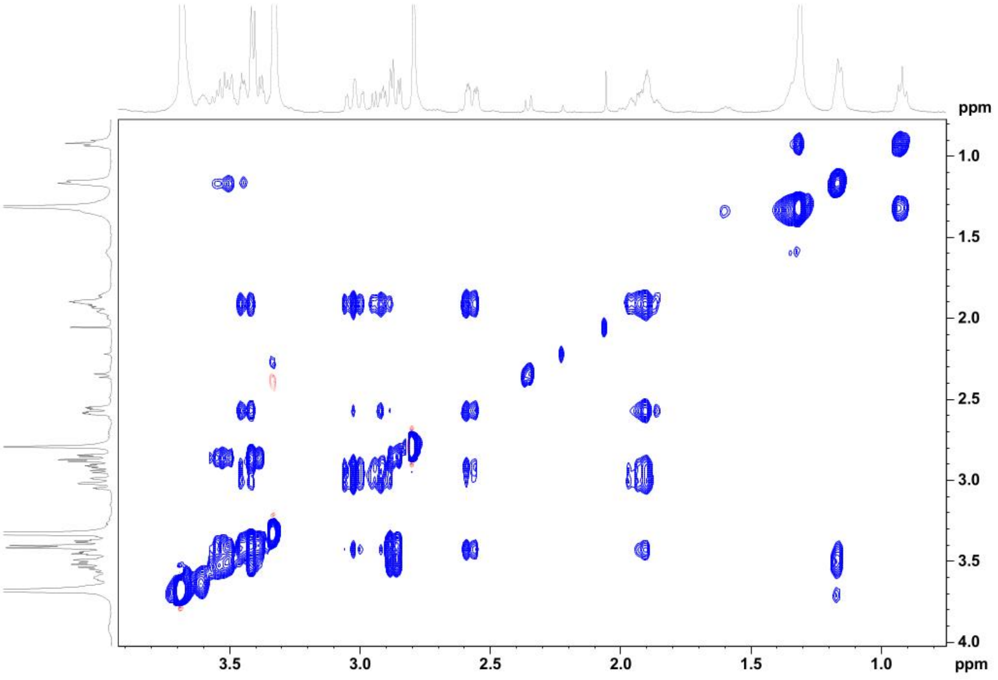

Figure S18. Aliphatic region of the ^{1}H-^{1}H TOCSY NMR spectrum of ^{CF3}**1** in CD_3OD at 298 K (400 MHz).

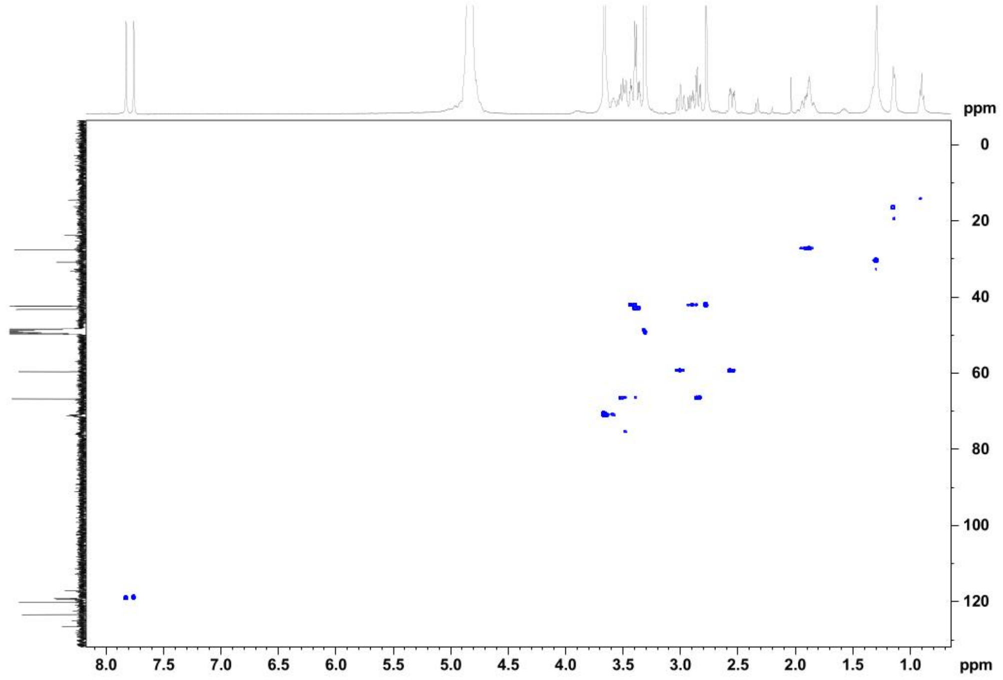

Figure S19. ^{1}H-^{13}C HSQC NMR spectrum of ^{CF3}**1** in CD_3OD at 298 K (400 MHz).

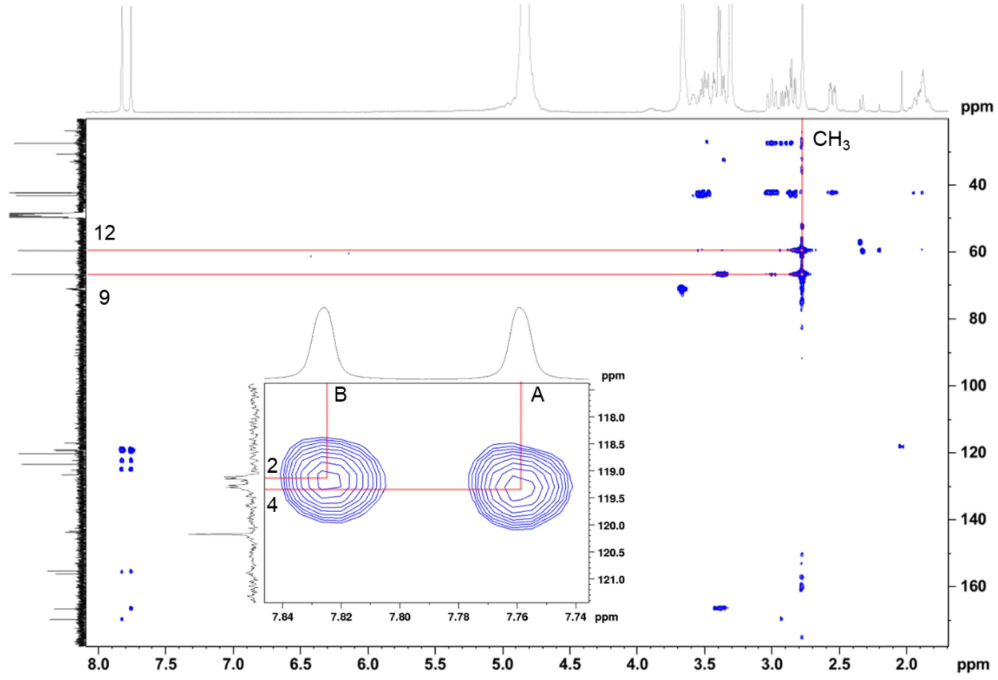

Figure S20. ^{1}H-^{13}C HMBC NMR spectrum of ^{CF3}**1** in CD_3OD at 298 K (400 MHz).

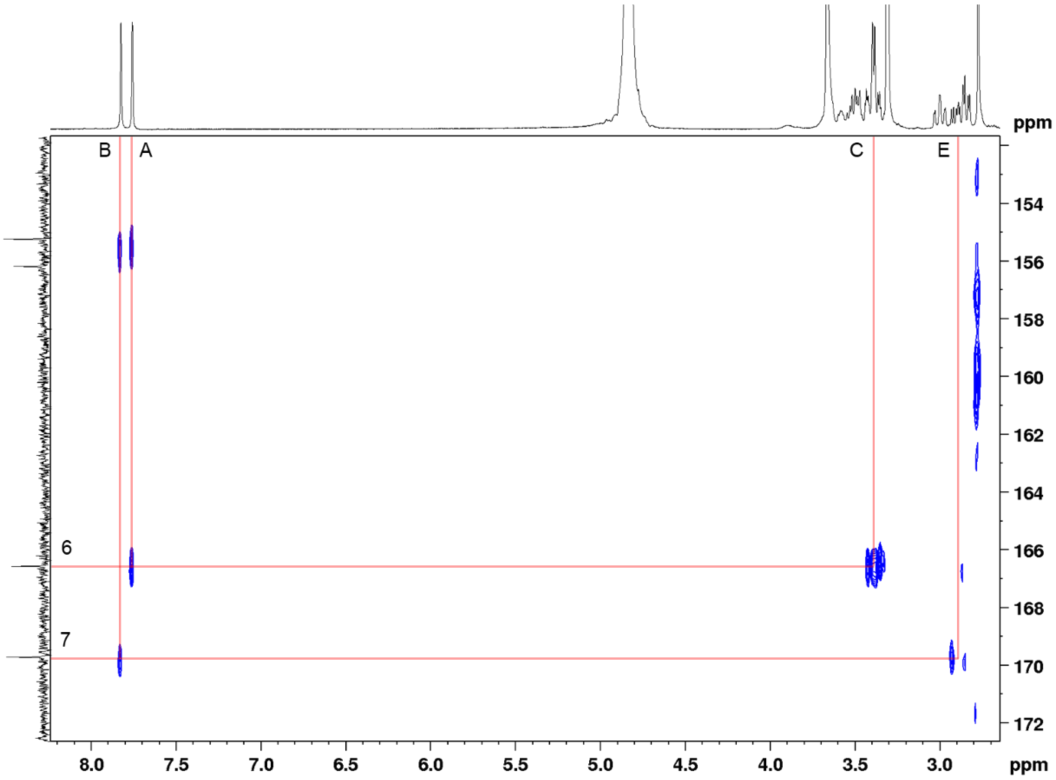

Figure S21. Zoom-in of the ^{1}H-^{13}C HMBC NMR spectrum of ^{CF3}**1** in CD_3OD at 298 K (400 MHz).

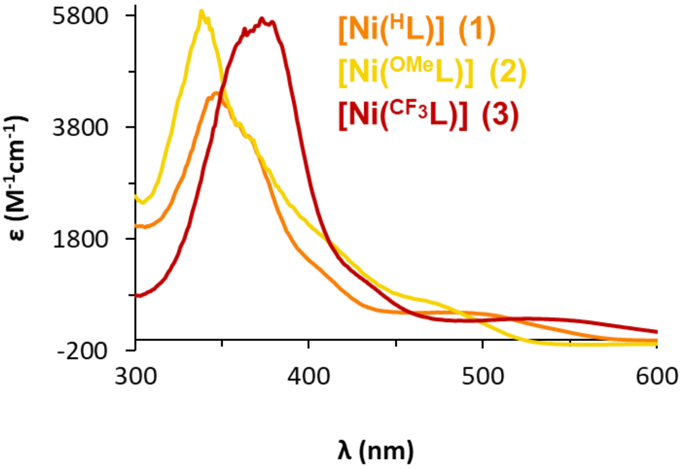

Figure S22. UV-vis spectra of ^{H}**1**, ^{OMe}**1** and ^{CF3}**1** in CH_3CN at room temperature.

3.3. Crystallographic data for [Ni(^{OMe}L)] and [Ni(^{CF3}L)]

Table S1. Crystallographic data for **[Ni(^{OMe}L)]** and **[Ni(^{CF3}L)]**.

	**[Ni(^{OMe}L)]**	**[Ni(^{CF3}L)]**
**Chemical formula**	$C_{62.60}H_{86.40}F_9N_{16}Na_3Ni_4O_{24.60}S_3$	$C_{29}H_{30}F_9N_8NaNi_2O_7S$
**Formula weight**	2027.66 g/mol	946.08 g/mol
**Temperature**	100(2) K	100(2) K
**Wavelength**	0.71076 Å	0.71076 Å
**Crystal size**	0.140 x 0.300 x 0.450 mm	0.040 x 0.100 x 0.300 mm
**Crystal habit**	orange-yellow plate	red plate
**Crystal system**	orthorhombic	orthorhombic
**Space group**	P b c n	P n a 21
**Unit cell dimensions**	a = 13.129(12) Å α = 90°<br>b = 21.38(2) Å β = 90°<br>c = 33.52(3) Å γ = 90°	a = 20.99(2) Å α = 90°<br>b = 9.448(11) Å β = 90°<br>c = 17.61(2) Å γ = 90°
**Volume**	9409.(15) Å^3	3492.(7) Å^3
**Z**	4	4
**Density (calculated)**	1.431 g/cm^3	1.799 g/cm^3
**Absorption coefficient**	0.960 mm^{-1}	1.259 mm^{-1}
**F(000)**	4179	1920
**Diffractometer**	Bruker D8 QUEST ECO three-circle diffractometer	Bruker D8 QUEST ECO three-circle diffractometer
**Radiation source**	Ceramic X-ray tube (Mo Kα,<br>λ = 0.71076 Å)	Ceramic X-ray tube (Mo Kα,<br>λ = 0.71076 Å)
**Theta range for data collection**	2.58 to 24.79°	2.63 to 28.48°
**Index ranges**	-15<=h<=15, -25<=k<=25, -39<=l<=39	-28<=h<=27, -12<=k<=12, -23<=l<=23
**Reflections collected**	263260	59640
**Independent reflections**	8054 [R(int) = 0.0438]	8768 [R(int) = 0.0553]
**Coverage of independent reflections**	99.4%	99.5%
**Absorption correction**	Multi-Scan	Multi-Scan
**Max. and min. transmission**	0.8770 and 0.6720	0.7457 and 0.6520
**Structure solution technique**	direct methods	direct methods
**Structure solution program**	SHELXT 2014/5 (Sheldrick, 2014)	SHELXT 2014/5 (Sheldrick, 2014)
**Refinement method**	Full-matrix least-squares on F^2	Full-matrix least-squares on F^2
**Refinement program**	SHELXL-2017/1 (Sheldrick, 2017)	SHELXL-2017/1 (Sheldrick, 2017)
**Function minimized**	Σ w(F$_o^2$ - F$_c^2$)^2	Σ w(F$_o^2$ - F$_c^2$)^2
**Data / restraints / parameters**	8054 / 1 / 591	8768 / 484 / 715
**Goodness-of-fit on F^2**	1.098	1.170
**Final R indices**	7285 data; I>2σ(I) R1 = 0.0918, wR2 = 0.2467<br>*all data* R1 = 0.0986, wR2 = 0.2518	7842 data; I>2σ(I) R1 = 0.0826, wR2 = 0.1881<br>*all data* R1 = 0.0919, wR2 = 0.1940
**Weighting scheme**	w=1/[σ^2(F$_o^2$)+(0.0943P)^2+104.4090P]<br>where P=(F$_o^2$+2F$_c^2$)/3	w=1/[σ^2(F$_o^2$)+(0.0943P)^2+104.4090P]<br>where P=(F$_o^2$+2F$_c^2$)/3
**Largest diff. peak and hole**	1.593 and -1.130 eÅ^{-3}	0.882 and -1.578 eÅ^{-3}
**R.M.S. deviation from mean**	0.143 eÅ^{-3}	0.144 $^{-3}$

4. Generation and reactivity of ^{X}2

4.1. Generation of ^{X}2

The experimental procedure was analogous to the one previously reported for the generation of ^{H}**2**.[1] In a typical experiment, 2.5 mL of a 0.15 mM soltuion of ^{X}**1** in CH_3CN were placed in a 1 cm path-length cuvette (0.38 μmols of ^{X}**1**). The quartz cell was placed in the Unisoku cryostat of the UV-vis absorption spectrophotometer and cooled down to -30 ºC. After reaching thermal equilibrium an UV-vis absorption spectrum of the starting complex was recorded. Then, 19 μL of a 0.1 M solution of acetic acid (5 equiv) in CH_3CN were added followed by 38 μL of a 0.05 M solution of NaOCl·5H_2O in CH_3CN:H_2O (4:1) (3 equiv). The formation of a band at λ_{max} = 470 nm (ε = 10400 $M^{-1}cm^{-1}$) for ^{H}**2**, 467 nm (ε = 9200 $M^{-1}cm^{-1}$) for ^{OMe}**2** and λ_{max} = 472 nm (ε = 8200 $M^{-1}cm^{-1}$) for ^{CF3}**2** was observed. The formation of ^{H}**2**, ^{OMe}**2** and ^{CF3}**2** reached their maximum in less than 2 min.

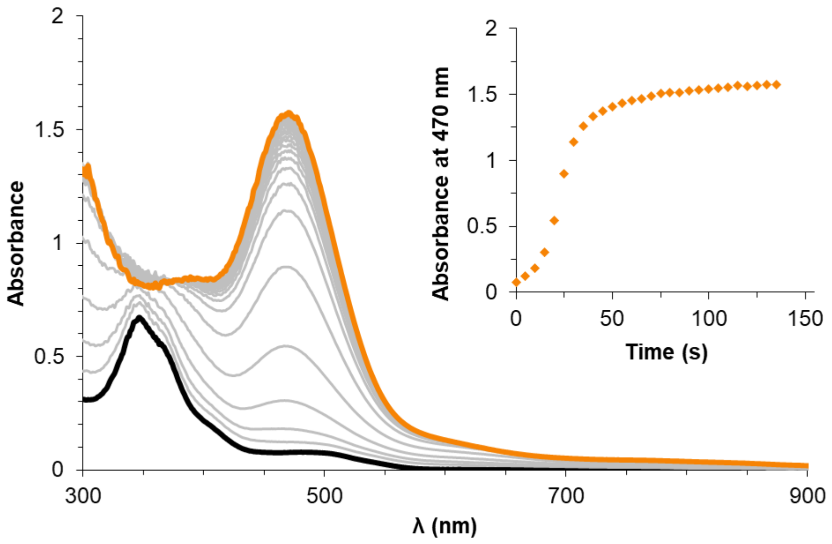

Figure S23. UV-vis spectral changes upon reaction of ^{H}**1** with 5 equiv NaOCl in the presence of acetic acid (5 equiv) in CH_3CN:H_2O 95:5 (v:v) at -30 ºC. Inset: kinetic trace at 470 nm.

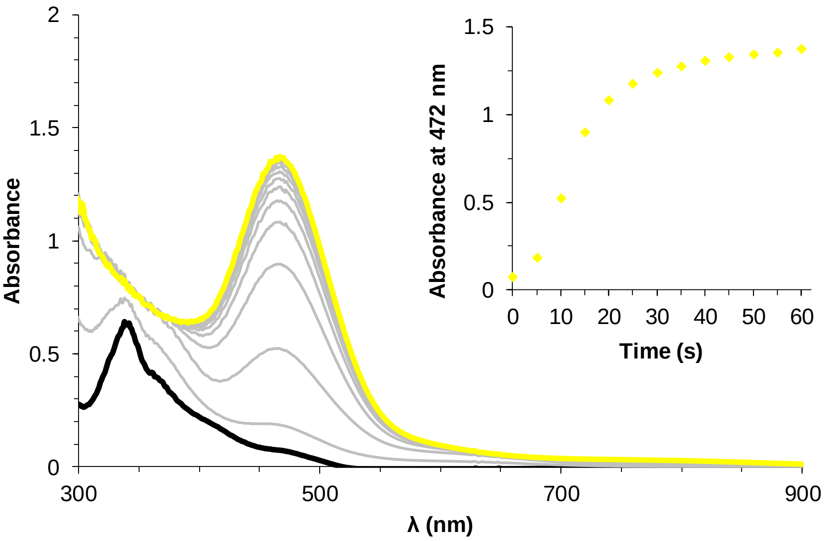

Figure S24. UV-vis spectral changes upon reaction of ^{OMe}**1** with 5 equiv NaOCl in the presence of acetic acid (5 equiv) in CH_3CN:H_2O 95:5 (v:v) at -30 ºC. Inset: kinetic trace at 467 nm.

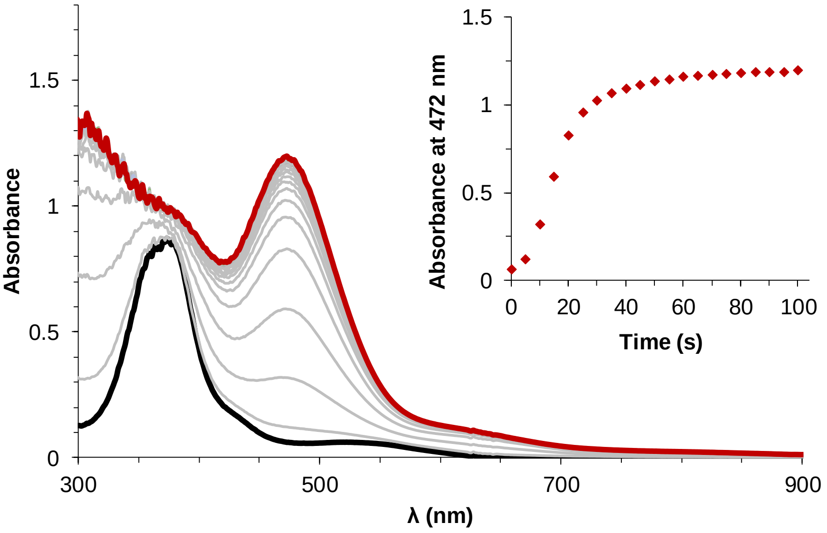

Figure S25. UV-vis spectral changes upon reaction of ^{CF3}**1** with 5 equiv NaOCl in the presence of acetic acid (5 equiv) in CH_3CN:H_2O 95:5 (v:v) at -30 ºC. Inset: kinetic trace at 472 nm.

4.2. Kinetic analyses of the reaction of ^{X}2 with organic substrates.

Once ^{X}**2** were fully formed (see above) the appropriate amount of substrate (1-octene or 1,4-cyclohexadiene) dissolved in 100 μL CH_3CN was directly added into the UV-vis cuvette. Substrate concentration was always in pseudo-first order excess with respect to ^{X}**2**. Reaction kinetics were monitored by following the decay of their absorption band at 470 nm for ^{H}**2**, 467 nm for ^{OMe}**2** and 472 nm for ^{CF3}**2**. In all cases, a satisfactory fit was obtained for the disappearance of ^{X}**2** using a

single exponential, from which observed rate constants (k_{obs}) were extracted. The linear variation of k_{obs} with substrate concentration enabled the calculation of the second-order rate constants (k).

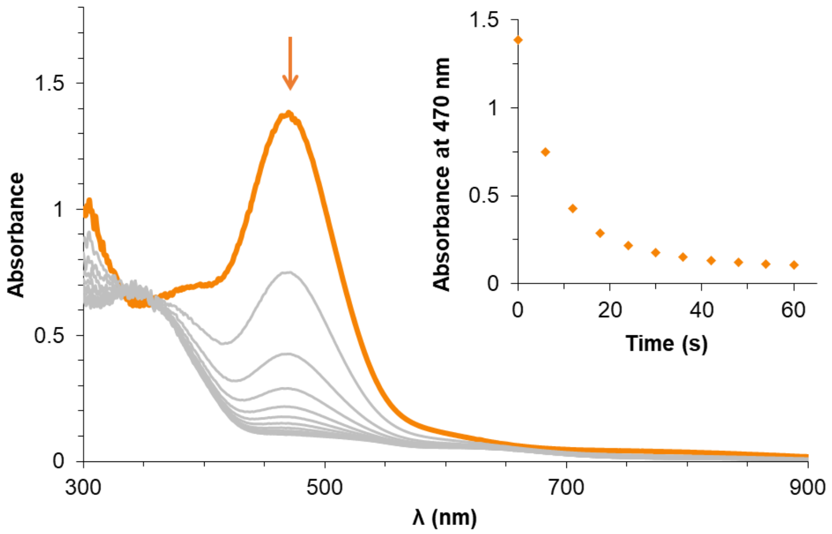

Figure S26. UV-vis spectral changes of a solution of ^{H}**2** (0.15 mM) upon addition of 157 equiv 1,4-cyclohexadiene in CH_3CN:H_2O 95:5 (v:v) at -30 ºC. Inset: Kinetic trace at 470 nm.

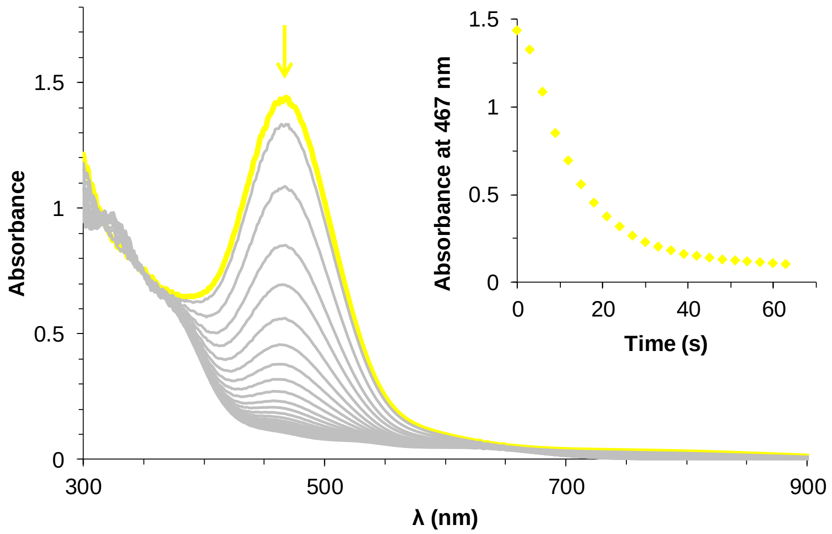

Figure S27. UV-vis spectral changes of a solution of ^{OMe}**2** (0.15 mM) upon addition of 157 equiv 1,4-cyclohexadiene in CH_3CN:H_2O 95:5 (v:v) at -30 ºC. Inset: Kinetic trace at 467 nm.

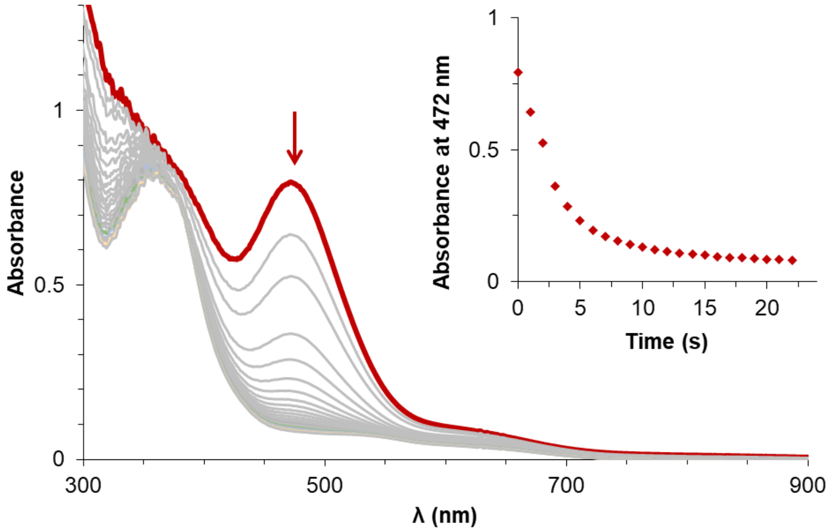

Figure S28. UV-vis spectral changes of a solution of ^{CF3}**2** (0.15 mM) upon addition of 157 equiv 1,4-cyclohexadiene in $CH_3CN:H_2O$ 95:5 (v:v) at -30 ºC. Inset: Kinetic trace at 472 nm.

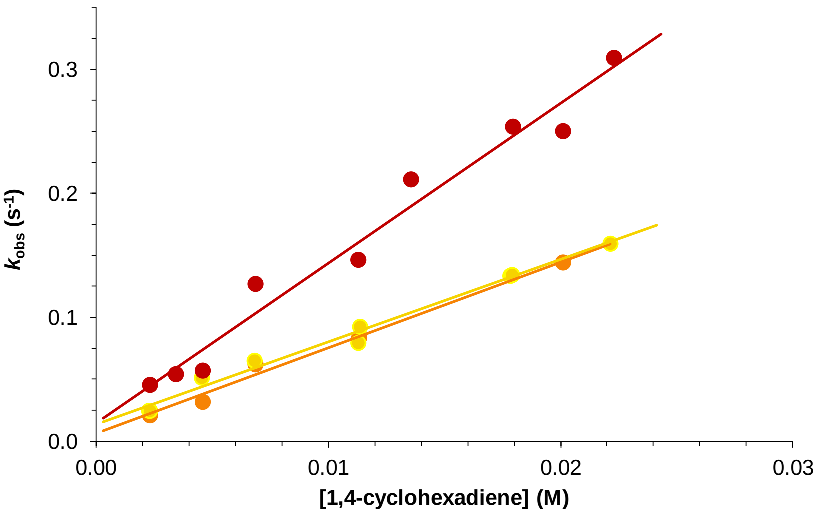

Figure S29. Plot of k_{obs} against substrate concentration for the reaction of ^{H}**2** (orange), ^{OMe}**2** (yellow) and ^{CF3}**2** (red) with 1,4-cyclohexadiene in $CH_3CN:H_2O$ 95:5 (v:v) at -30 ºC.

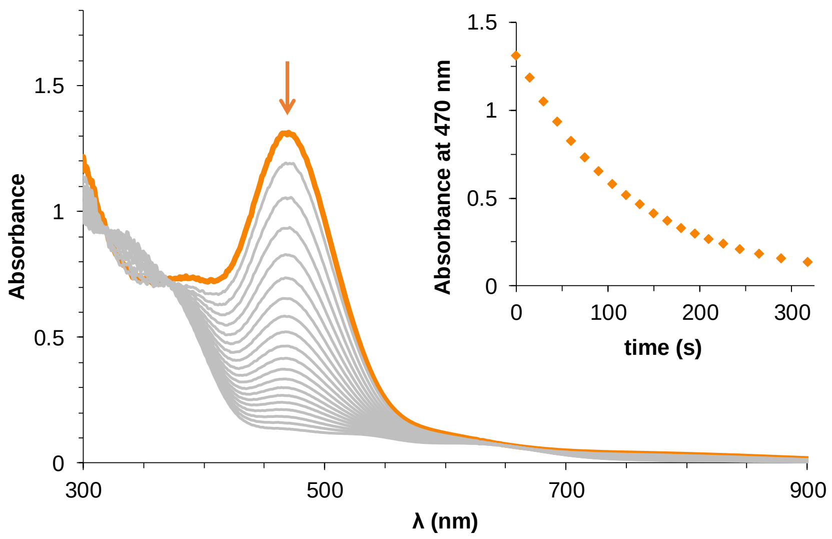

Figure S30. UV-vis spectral changes of a solution of ^{H}**2** (0.15 mM) upon addition of 165 equiv 1-octene in $CH_3CN:H_2O$ 95:5 (v:v) at -30 ºC. Inset: Kinetic trace at 470 nm.

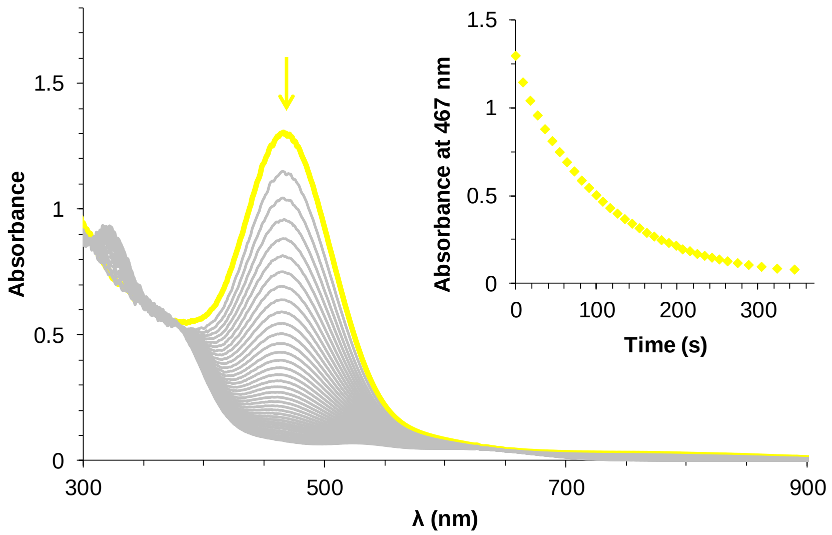

Figure S31. UV-vis spectral changes of a solution of ^{OMe}**2** (0.15 mM) upon addition of 165 equiv 1-octene in $CH_3CN:H_2O$ 95:5 (v:v) at -30 ºC. Inset: Kinetic trace at 467 nm.

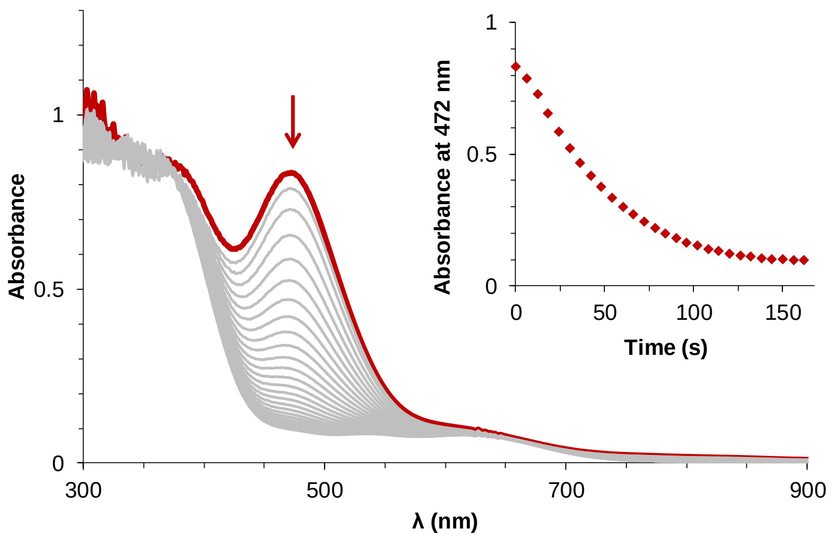

Figure S32. UV-vis spectral changes of a solution of ^{CF3}**2** (0.15 mM) upon addition of 165 equiv 1-octene in $CH_3CN:H_2O$ 95:5 (v:v) at -30 ºC. Inset: Kinetic trace at 472 nm.

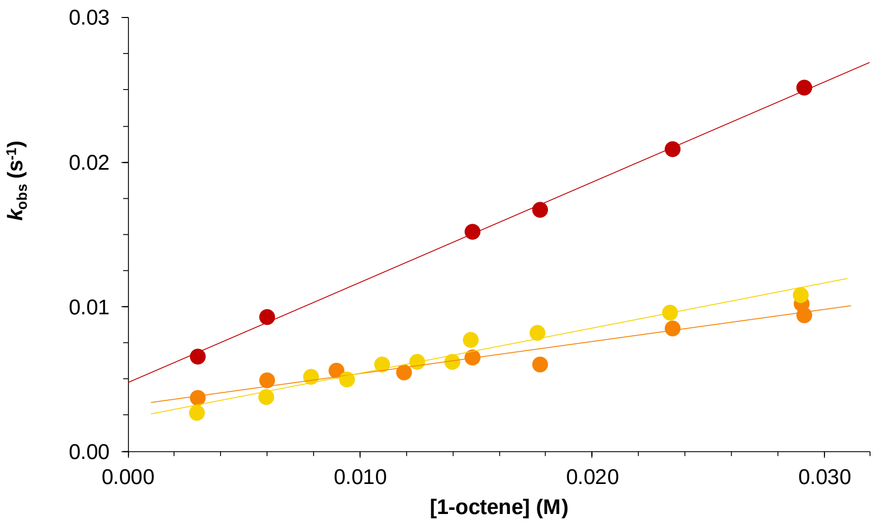

Figure S33. Plot of k_{obs} against substrate concentration for the reaction of ^{H}**2** (orange), ^{OMe}**2** (yellow) and ^{CF3}**2** (red) with 1-octene in $CH_3CN:H_2O$ 95:5 (v:v) at -30 ºC.

5. Theoretical calculations

5.1. Computational details

All geometry optimizations were performed using the B3LYP[10-11] density functional in combination with the def2-SVP basis set, including empirical dispersion correction of Grimme (D3)[12] with the Becke-Johnson (BJ)[13] damping function. The nature of the stationary points was confirmed by harmonic frequency calculations in all cases. Gibb's free energy corrections were obtained using the standard statistical-mechanics relationships for an ideal gas at 243.15 K. Single-point calculations using the def2-TZVP basis were performed at all stationary points to improve the electronic energy. Solvation effects were included for both the geometry optimizations and single-point calculations using the implicit SMD solvation model with standard parameters for acetonitrile.

Spin-resolved effective fragment orbitals (EFOs) and subsequent EOS analysis were performed with the APOST-3D program[14] using the Topological Fuzzy Voronoi Cells (TFVC)[15] real-space atomic definition.

5.2. Mechanism of the HAT

In the low spin (S=1/2) surface, the reaction proceeds via an asynchronous concerted mechanism, with no intermediate between the HAT and the subsequent rebound, as shown in the IRC path of Figure S34. The transfer of H takes place during the first seven steps of the IRC, going from an O-H distance of 1.53 Å down to 0.99 Å. The O-H distance then remains essentially unchanged while the Ni-OH bond gradually rotates to facilitate the rebound. We have followed the partial charge and condensed spin density of Ni, macrocyclic ligand, OH ligand, and the substrate along these first steps of the IRC. The results are depicted in Figure S35. Surprisingly, both the partial charge and spin of the C atom of the substrate are very close to zero during these steps. Indeed, it can be clearly seen that the LUMO corresponds to a lone pair sitting in the C atom. Still, the C atom remains neutral. If one instead considers the partial charge and spin of the whole substrate, not just the contact atom, the picture becomes more clear. The partial charge gradually increases as the H is transferred to the oxyl fragment and reaches a value of ca. +0.75, thus indicating a formal hydride transfer. The mechanism of this transfer is however difficult to discern because of the delocalized nature of the electronic state. One can see an early and sudden increase in the spin density of Ni in the very first steps, from -0.17 to 0.68. However, this does not translate into a change in its partial charge, which remains remarkably constant at ca +1.40. At the same time, the spin density on the OH group rapidly decreases from +0.85 to +0.19, while its partial charge remains again constant at ca. -0.50. This indicates that, on average, the alpha electron of the C-

H bond is transferred to the OH group, while the beta electron is transferred to the Ni. The excess charge that compensates for the cationic nature of the substrate is gained by the macrocyclic ligand. This significant electron flow between the fragments translates into huge changes in the projected dipole moment, as shown in Figure S36.

On the other hand, Figure S37 shows the evolution of the charge and spin densities of the fragments along the IRC of the quadruplet state. Contrary to the rather involved situation of the S=1/2 state, in the S=3/2 path, the substrate’s spin density gradually increases as the O-H distance decreases, forming a clear radical substrate. At the same time, the charge and spin of the Ni center remain remarkably constant, and only the spin density of the OH moiety decreases by the transfer of a beta electron together with the H nucleus. Thus, the reaction follows a clear HAT mechanism, also supported by the much smaller change in the projected dipole moment, as compared to the low spin case (see Figure S38).

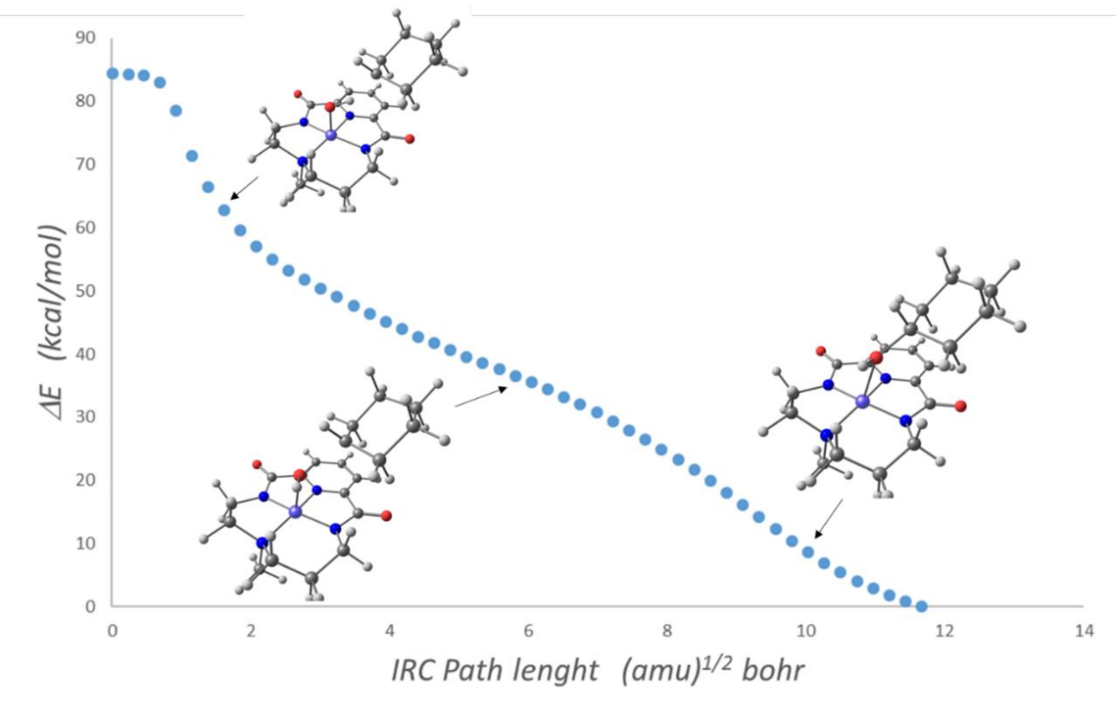

Figure S34. Intrinsic reaction path from **TS(II-III)** to **III** computed at the B3LYP-D3(BJ)/def2-svp level of theory for state *S*=1/2.

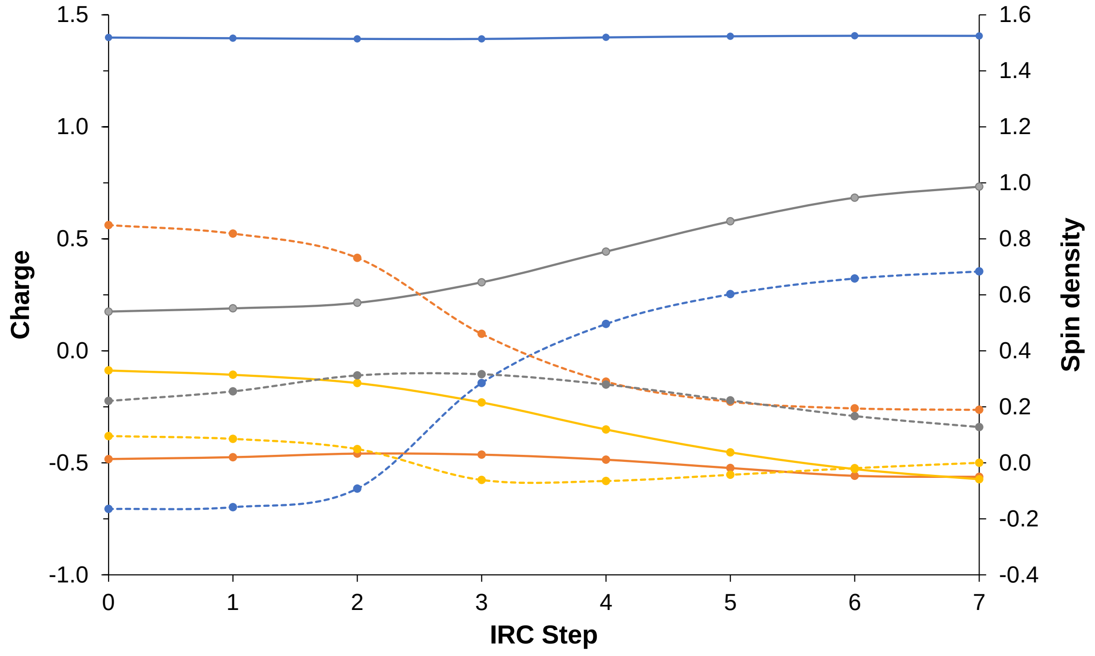

Figure S35. Evolution of the partial charges (solid lines) and condensed spin densities (dotted lines) of the Ni atom (blue), the OH fragment (orange), cyclohexane fragment (grey) and macrocyclic ligand (yellow) along the IRC path from **TS(II-III)$_{d}$** to **III$_{d}$** for $[Ni(O)(^{H}L)]^{+}$ species computed at the B3LYP-D3(BJ)/def2-svp level of theory for state *S*=1/2.

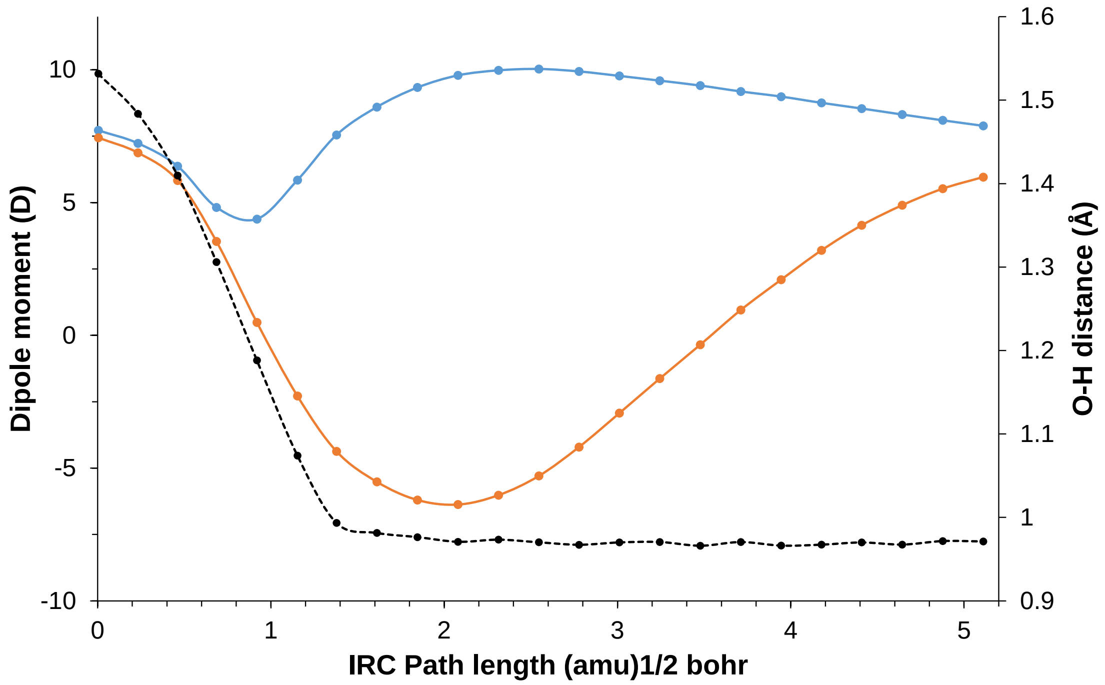

Figure S36. Evolution of the total dipole moment (blue line) and the projection of the dipole moment along the O-H vector (orange line), expressed in Debye, along the IRC path from **TS(II-III)$_{d}$** to **III$_{d}$** for $[Ni(O)(^{H}L)]^{+}$ species computed at the B3LYP-D3(BJ)/def2-svp level of theory for state *S*=1/2. O-H distance (dotted black line) in Å.

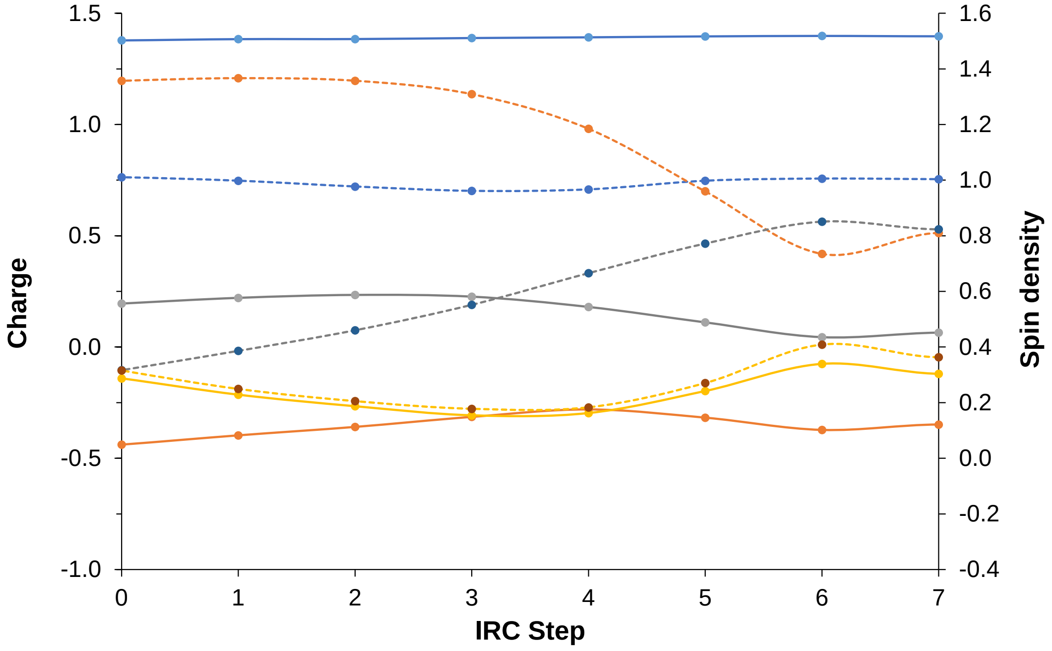

Figure S37. Evolution of the partial charges (solid lines) and condensed spin densities (dotted lines) of the Ni atom (blue), the OH fragment (orange), cyclohexane fragment (grey) and macrocyclic ligand (yellow) along the IRC path from **TS(II-III)$_\mathbf{q}$** to **III$^\mathbf{a}_\mathbf{q}$** for [Ni(O)(^{H}L)]$^{+}$ species computed at the B3LYP-D3(BJ)/def2-svp level of theory for state *S*=3/2.

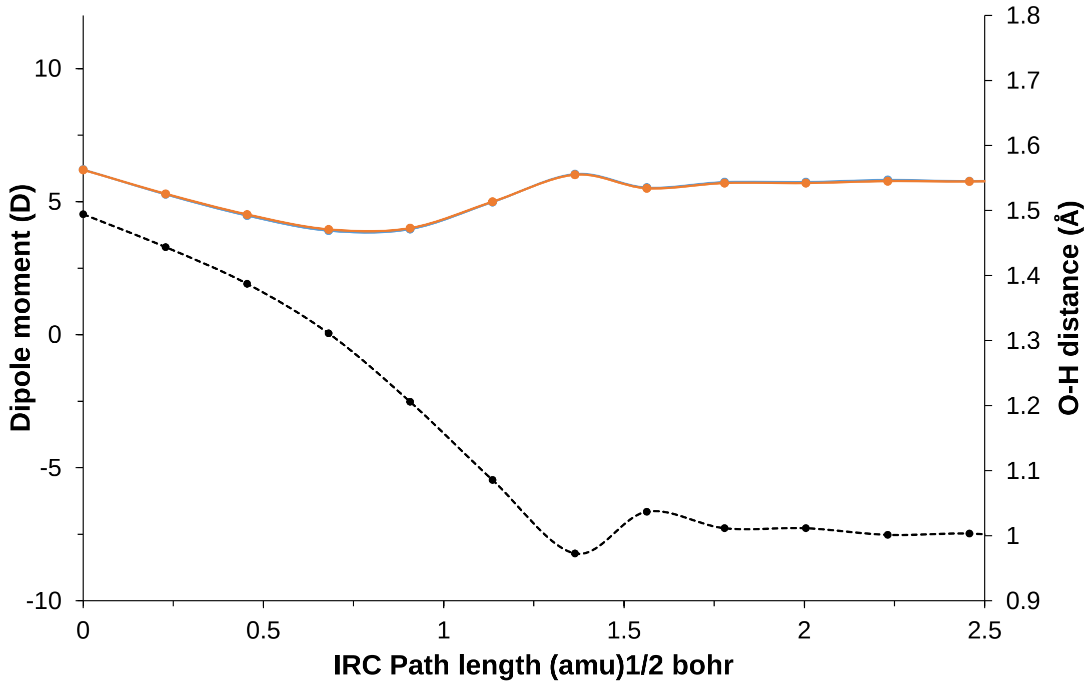

Figure S38 Evolution of the total dipole moment (blue line) and the projection of the dipole moment along the O-H vector (orange line), expressed in Debye along the IRC path from **TS(II-III)$_\mathbf{q}$** to **III$^\mathbf{a}_\mathbf{q}$** for [Ni(O)(^{H}L)]$^{+}$ species computed at the B3LYP-D3(BJ)/def2-svp level of theory for state *S*=3/2. O-H distance (dotted black line) in Å.

5.3. Additional figures for the mechanism of species ^{OMe}2 and ^{CF3}2

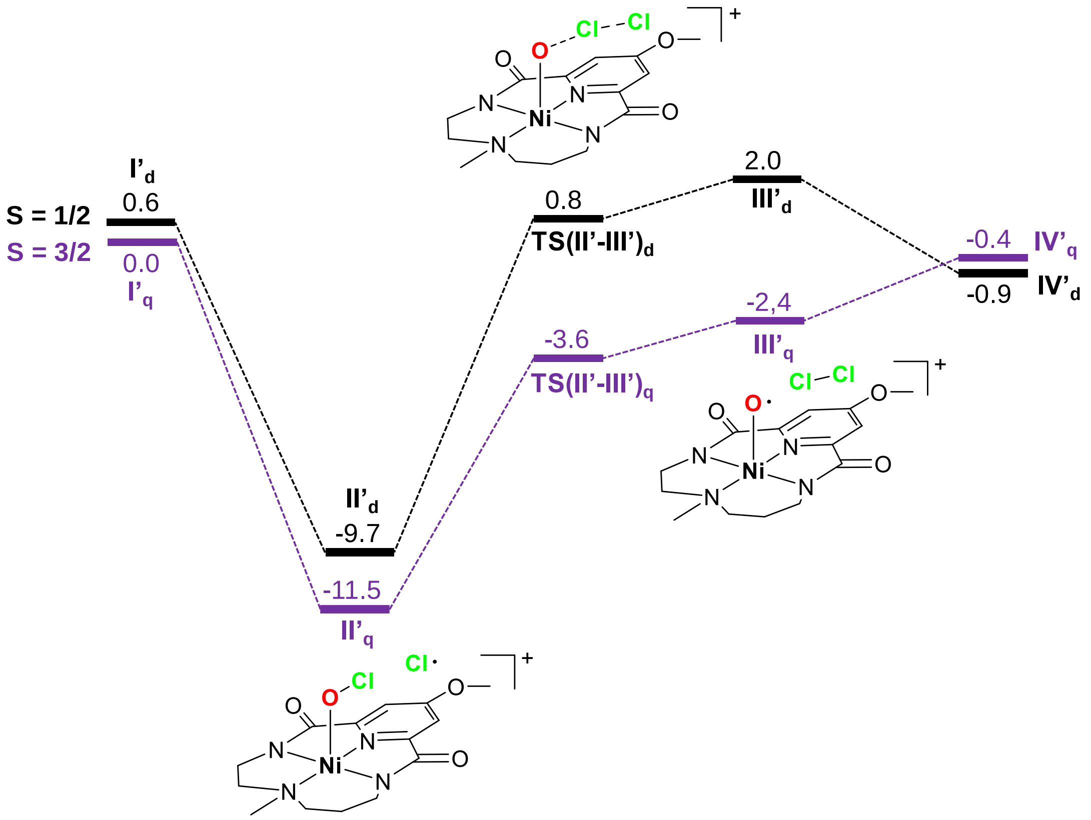

Figure S39. Reaction profile for the O–Cl cleavage in [Ni(OCl)(^{OMe}L)]$^+$ assisted by chlorine radical to afford [Ni(O)(^{OMe}L)]$^+$, computed at the B3LYP-D3(BJ)/def2-tzvp//B3LYP-D3(BJ)/def2-svp level of theory. Free energies are given in kcal·mol^{-1}. Subscripts d and q represent spin states *S* = 1/2 and *S* = 3/2, respectively.

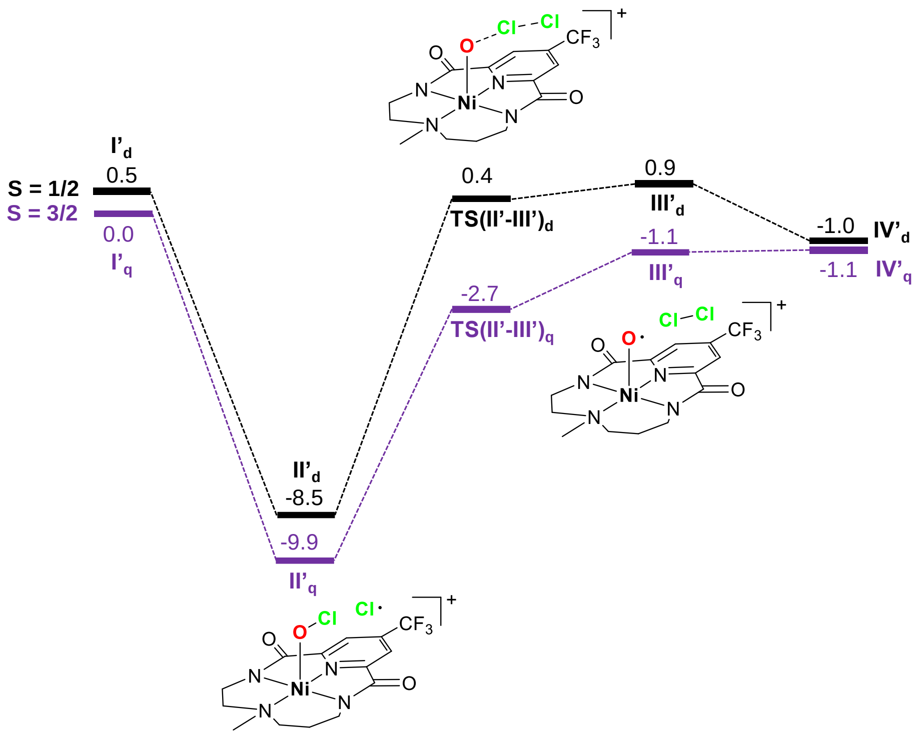

Figure S40. Reaction profile for the O–Cl cleavage in [Ni(OCl)(^{CF3}L)]$^+$ assisted by chlorine radical to afford [Ni(O)(^{CF3}L)]$^+$, computed at the B3LYP-D3(BJ)/def2-tzvp//B3LYP-D3(BJ)/def2-svp level of theory. Free energies are given in kcal·mol^{-1}. Subscripts d and q represent spin states S = 1/2 and S = 3/2, respectively.

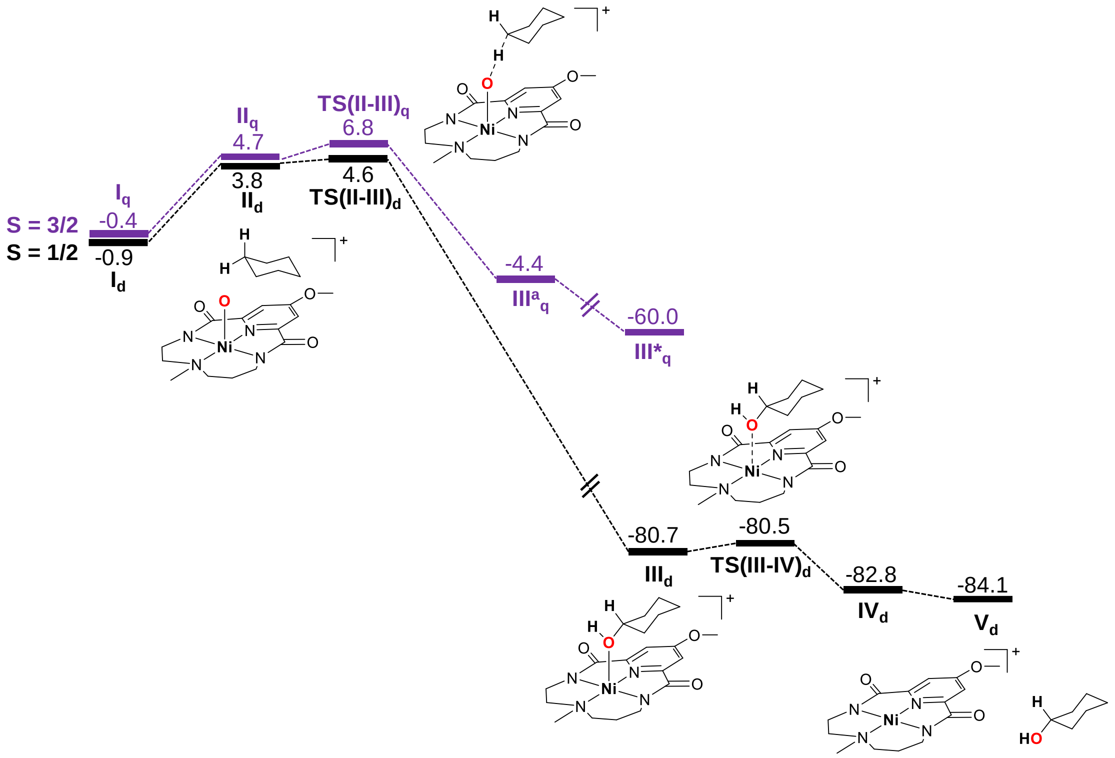

Figure S41. Reaction profile for the oxidation of cyclohexane carried out by the [Ni(O)(^{OMe}L)]$^+$ species computed at the B3LYP-D3(BJ)/def2-tzvp// B3LYP-D3(BJ)/def2-svp level of theory. Free energies are given in kcal·mol^{-1}. The drawn structures correspond to intermediates and transitions states of the *S* = 1/2 energy surface. Subscripts d and q represent spin states *S* = 1/2 and *S* = 3/2, respectively.

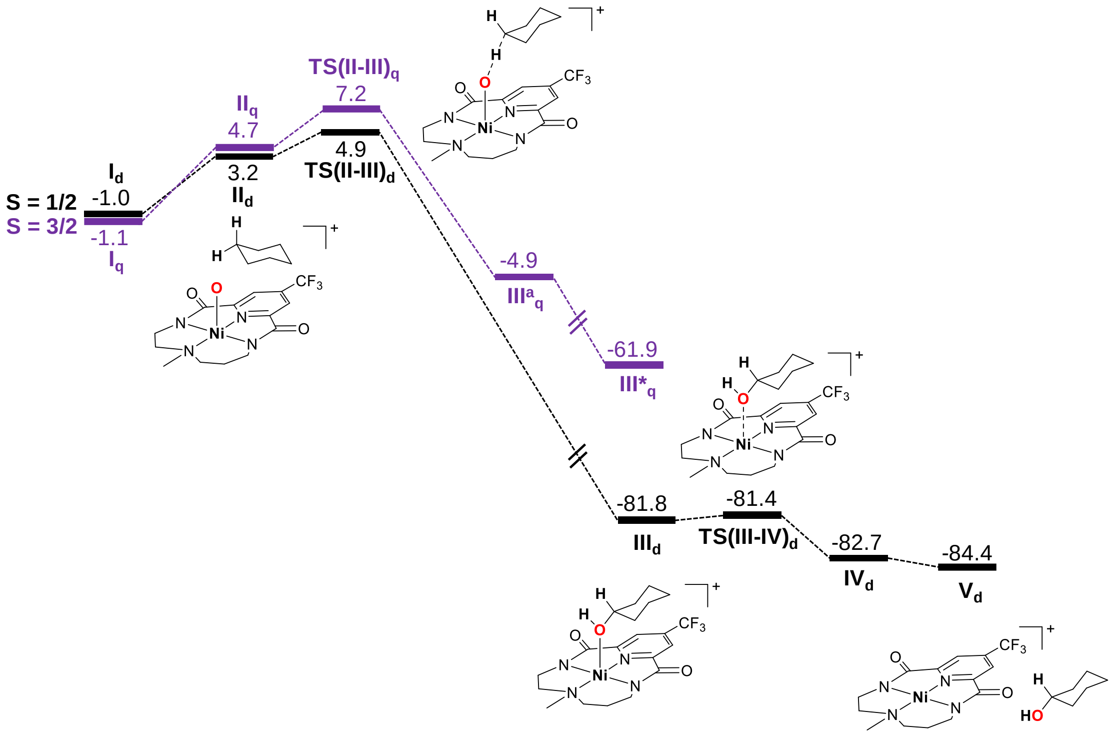

Figure S42. Reaction profile for the oxidation of cyclohexane carried out by the [Ni(O)(^{CF3}L)]$^+$ species computed at the B3LYP-D3(BJ)/def2-tzvp// B3LYP-D3(BJ)/def2-svp level of theory. Free energies are given in kcal·mol^{-1}. The drawn structures correspond to intermediates and transitions states of the *S* = 1/2 energy surface. Subscripts d and q represent spin states *S* = 1/2 and *S* = 3/2, respectively.

5.4. Additional Figures for the Effective fragment orbitals (EFOs)

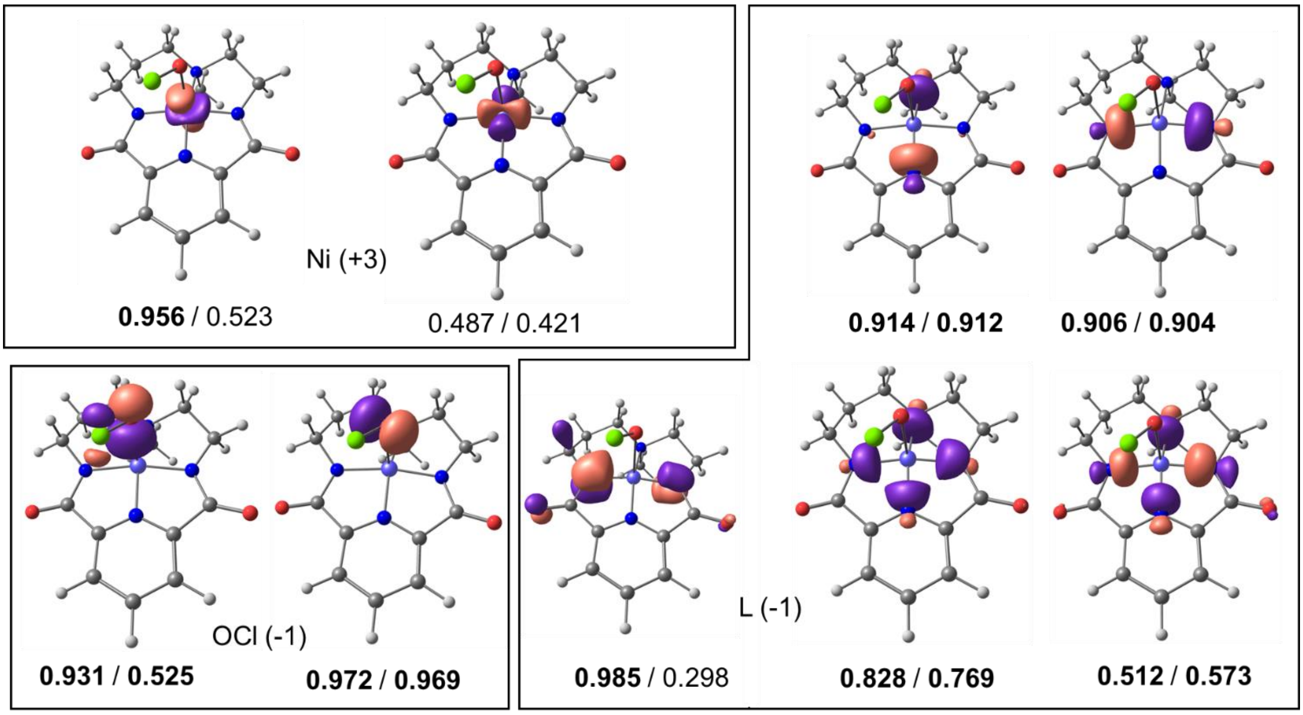

Figure S43. Relevant EFOs and their occupation numbers for the ^{H}**2** species in the triplet (S = 1) state. Occupations in bold indicate that the EFO is occupied in the EOS analysis.

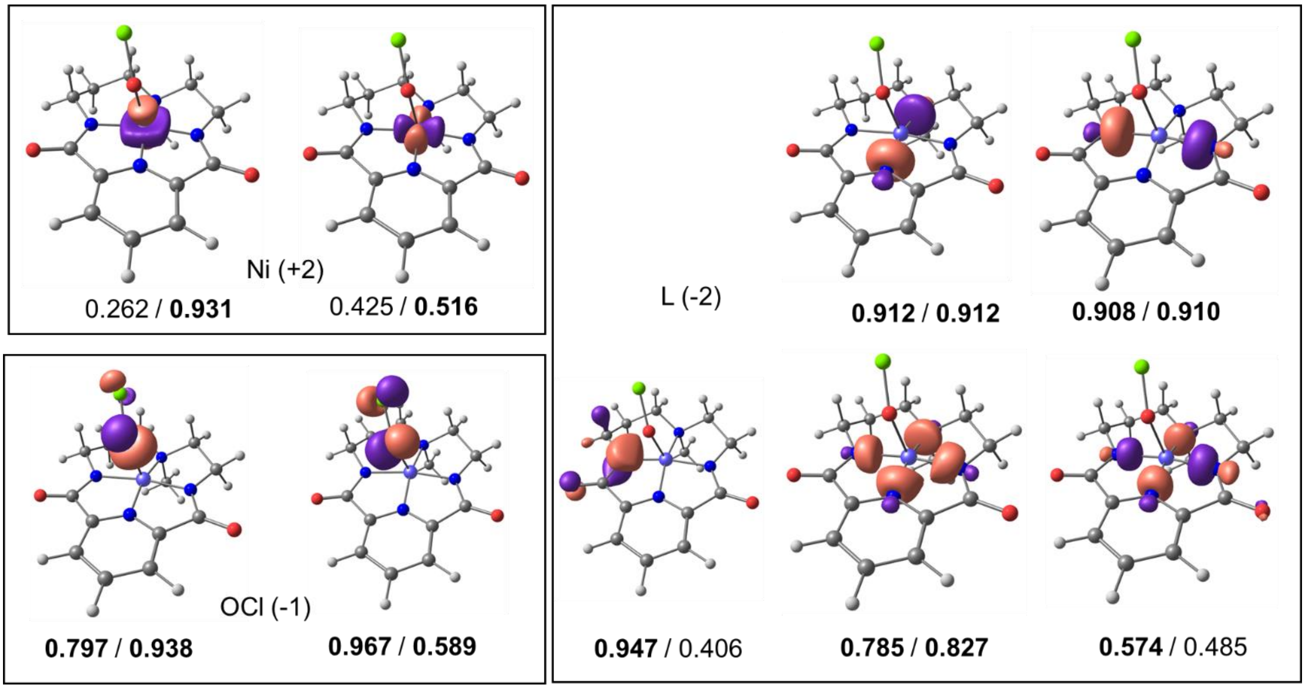

Figure S44. Relevant EFOs and their occupation numbers for the ^{H}**2** species in the open-shell singlet (S = 0) state. Occupations in bold indicate that the EFO is occupied in the EOS analysis.

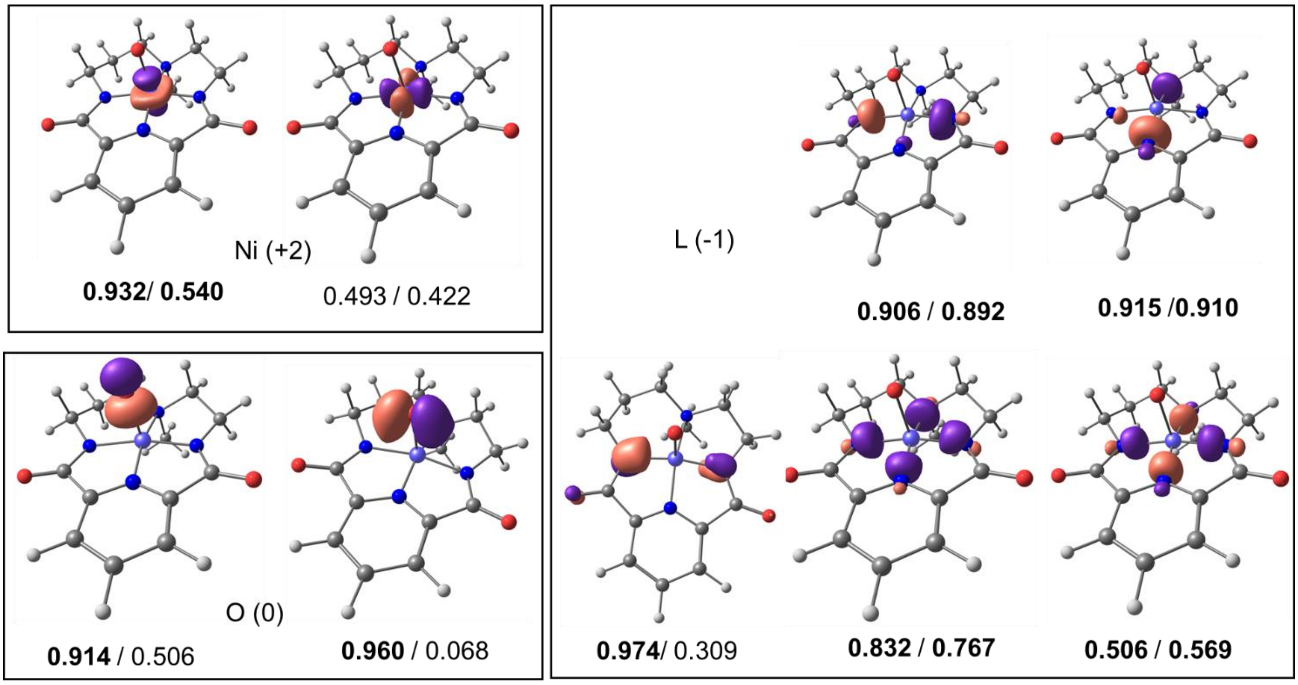

Figure S45. Relevant EFOs and their occupation numbers for the [Ni(O)(^{H}L)]$^{+}$ species in the *S*=3/2 state. Occupations in bold indicate that the EFO is occupied in the EOS analysis.

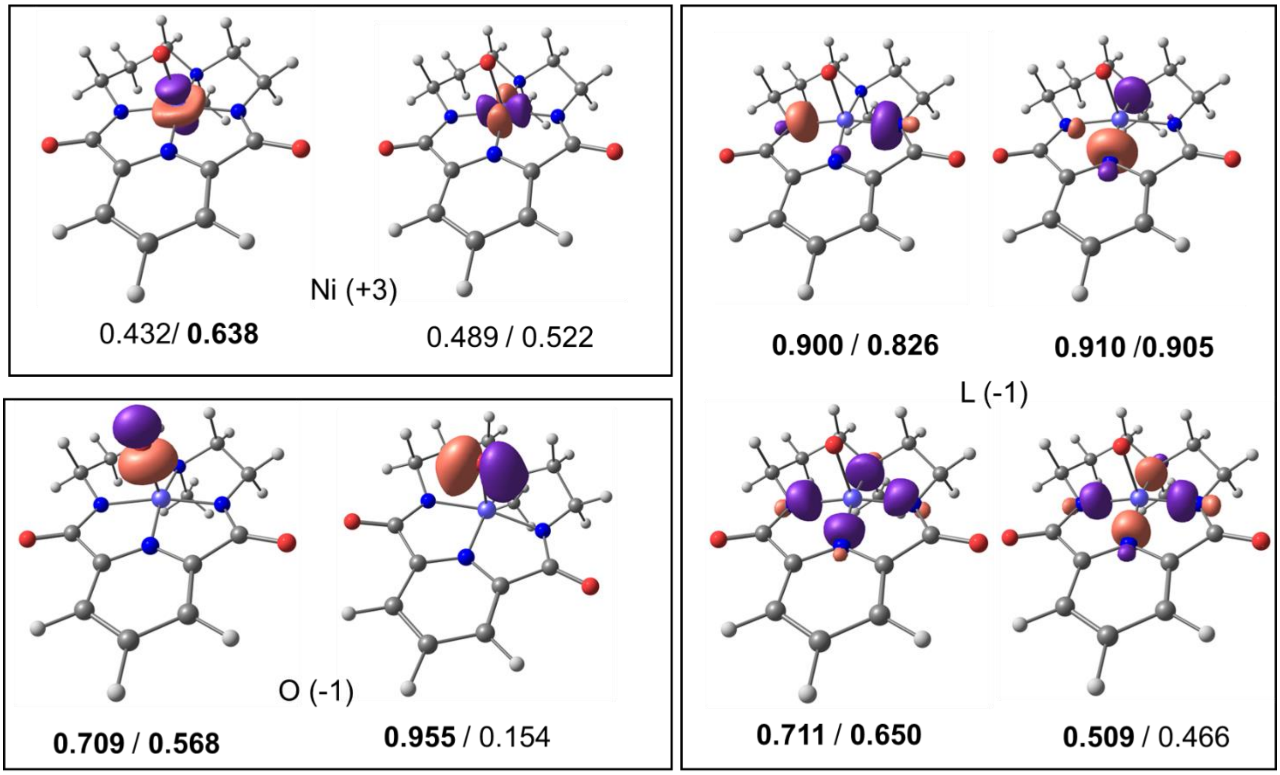

Figure S46. Relevant EFOs and their occupation numbers for the [Ni(O)(^{H}L)]$^{+}$ species in the *S*=1/2 state. Occupations in bold indicate that the EFO is occupied in the EOS analysis.

6. Bibliography

1. Corona, T.; Pfaff, F. F.; Acuña-Parés, F.; Draksharapu, A.; Whiteoak, C. J.; Martin-Diaconescu, V.; Lloret-Fillol, J.; Browne, W. R.; Ray, K.; Company, A., Reactivity of a Nickel(II) Bis(amidate) Complex with meta-Chloroperbenzoic Acid: Formation of a Potent Oxidizing Species. *Chem. Eur. J.* **2015,** *21*, 15029-15038.
2. Nisar Ahamed, B.; Duchêne, R.; Robeyns, K.; Fustin, C.-A., Catenane-based mechanically-linked block copolymers. *Chem. Commun.* **2016,** *52*, 2149-2152.
3. Fan, R.; Serrano-Plana, J.; Oloo, W. N.; Draksharapu, A.; Delgado-Pinar, E.; Company, A.; Martin-Diaconescu, V.; Borrell, M.; Lloret-Fillol, J.; García-España, E.; Guo, Y.; Bominaar, E. L.; Que, L.; Costas, M.; Münck, E., Spectroscopic and DFT Characterization of a Highly Reactive Nonheme Fe^{V}–Oxo Intermediate. *J. Am. Chem. Soc.* **2018,** *140*, 3916-3928.
4. Müller, S.; Sanders, D. A.; Di Antonio, M.; Matsis, S.; Riou, J.-F.; Rodriguez, R.; Balasubramanian, S., Pyridostatin analogues promote telomere dysfunction and long-term growth inhibition in human cancer cells. *Org. Biomol. Chem.* **2012,** *10*, 6537-6546.
5. Kupai, J.; Kisszékelyi, P.; Rojik, E.; Dargó, G.; Hegedűs, L.; Bezzegh, D.; Maszler, P.; Szabó, L.; Németh, T.; Tibor Balogh, G.; Huszthy, P., Synthesis and determination of pKa values of new enantiopure pyridino- and piperidino-18-crown-6 ethers. *ARKIVOC* **2016,** *IV*, 130-151.
6. Monteiro, J. H. S. K.; Machado, D.; de Hollanda, L. M.; Lancellotti, M.; Sigoli, F. A.; de Bettencourt-Dias, A., Selective cytotoxicity and luminescence imaging of cancer cells with a dipicolinato-based EuIII complex. *Chem. Commun.* **2017,** *53*, 11818-11821.
7. Chessa, G.; Canovese, L.; Visentin, F.; Santo, C.; Seraglia, R., Synthesis of poly(pyridylthioether) dendrimers incorporating a Fe2(CO)6 cluster core. *Tetrahedron* **2005,** *61*, 1755-1763.
8. Picot, A.; Feuvrie, C.; Barsu, C.; Malvolti, F.; Le Guennic, B.; Le Bozec, H.; Andraud, C.; Toupet, L.; Maury, O., Synthesis, structures, optical properties, and TD-DFT studies of donor-π-conjugated dipicolinic acid/ester/amide ligands. *Tetrahedron* **2008,** *64*, 399-411.
9. Parsons, A. T.; Johnson, J. S., Catalytic Enantioselective Synthesis of Tetrahydrofurans: A Dynamic Kinetic Asymmetric [3 + 2] Cycloaddition of Racemic Cyclopropanes and Aldehydes. *J. Am. Chem. Soc.* **2009,** *131*, 3122-3123.
10. Becke, A. D., Density-functional thermochemistry. III. The role of exact exchange. *The Journal of Chemical Physics* **1993,** *98*, 5648-5652.
11. Lee, C.; Yang, W.; Parr, R. G., Development of the Colle-Salvetti correlation-energy formula into a functional of the electron density. *Phys Rev B Condens Matter* **1988,** *37*, 785-789.
12. Grimme, S.; Antony, J.; Ehrlich, S.; Krieg, H., A consistent and accurate ab initio parametrization of density functional dispersion correction (DFT-D) for the 94 elements H-Pu. *J Chem Phys* **2010,** *132*, 154104.
13. Grimme, S.; Ehrlich, S.; Goerigk, L., Effect of the damping function in dispersion corrected density functional theory. *J Comput Chem* **2011,** *32*, 1456-65.
14. Salvador, P.; Ramos-Cordoba, E.; Gimferrer, M.; Montilla, M., APOST-3D Program. Universitat de Girona. 2020.
15. Salvador, P.; Ramos-Cordoba, E., Communication: An approximation to Bader's topological atom. *J Chem Phys* **2013,** *139*, 071103.
